# Biobank-scale inference of ancestral recombination graphs enables genealogical analysis of complex traits

**DOI:** 10.1038/s41588-023-01379-x

**Published:** 2023-05-01

**Authors:** Brian C. Zhang, Arjun Biddanda, Árni Freyr Gunnarsson, Fergus Cooper, Pier Francesco Palamara

**Affiliations:** 1 Department of Statistics, University of Oxford, Oxford, UK; 2 Wellcome Centre for Human Genetics, University of Oxford, Oxford, UK; 3 Department of Computer Science, University of Oxford, Oxford, UK

**Keywords:** Genome-wide association studies, Population genetics

## Abstract

Genome-wide genealogies compactly represent the evolutionary history of a set of genomes and inferring them from genetic data has the potential to facilitate a wide range of analyses. We introduce a method, ARG-Needle, for accurately inferring biobank-scale genealogies from sequencing or genotyping array data, as well as strategies to utilize genealogies to perform association and other complex trait analyses. We use these methods to build genome-wide genealogies using genotyping data for 337,464 UK Biobank individuals and test for association across seven complex traits. Genealogy-based association detects more rare and ultra-rare signals (*N* = 134, frequency range 0.0007−0.1%) than genotype imputation using ~65,000 sequenced haplotypes (*N* = 64). In a subset of 138,039 exome sequencing samples, these associations strongly tag (average *r* = 0.72) underlying sequencing variants enriched (4.8×) for loss-of-function variation. These results demonstrate that inferred genome-wide genealogies may be leveraged in the analysis of complex traits, complementing approaches that require the availability of large, population-specific sequencing panels.

## Abstract (web summary)

ARG-Needle is a method to infer genome-wide genealogies from large-scale genotyping data that can be used in association analyses. Applied to UK Biobank data, genealogy-based testing finds more trait associations than using imputed genotypes.

## Main

Modeling genealogical relationships between individuals plays a key role in a wide range of analyses, including the study of natural selection^1^ and demographic history^2^, genotype phasing^3^ and imputation^4^. Due to the very large number of genealogical relationships that may give rise to observed genomic variation, data-driven inference of these relationships is computationally difficult^5^. For this reason, available methods for the inference of genealogies rely on strategies that trade model simplification for computational scalability, such as the use of approximate probabilistic models^5–11^, scalable heuristics^12–16^ or combinations of the two^17,18^. Recent advances enabled efficient estimation of the genealogical distance between genomic regions from ascertained genotype data^11^, rapid genealogical approximations for hundreds of thousands of samples^15^ and improved scalability of probabilistic inference^17^. However, available methods do not simultaneously offer all these features, so that scalable and accurate genealogical inference in modern biobanks remains challenging. In addition, these datasets contain extensive phenotypic information, but applications of inferred genealogies have primarily focused on evolutionary analyses. Early work suggested that genealogical data may be used to improve association and fine-mapping^13,19^, but the connections between genealogical inference and modern methodology for complex trait analysis^20–22^ remain under-explored.

We introduce a new algorithm, ARG-Needle, to accurately infer the ancestral recombination graph^23^ (ARG) for large collections of genotyping or sequencing samples. We demonstrate that the ARG of a sample may be used within a linear mixed model (LMM) framework to increase association power, detect association to unobserved genomic variants, infer narrow sense heritability and perform polygenic prediction. Using ARG-Needle, we infer the ARG for 337,464 UK Biobank samples and perform a genealogy-wide association scan for seven complex traits. We show that despite being inferred using only array data, the ARG detects more independent associations to rare and ultra-rare variants (minor allele frequency (MAF) < 0.1%) than imputation based on a reference panel of ~65,000 sequenced haplotypes of matched ancestry. We use 138,039 exome sequencing samples to confirm that these signals correspond to unobserved sequencing variants, which are strongly enriched for loss-of-function and other protein-altering variation and overlap with likely causal associations detected using within-cohort exome sequencing imputation. Using the ARG, we detect associations to variants as rare as MAF ≈ 4 × 10^−6^ and independent higher frequency variation that is not captured using imputation.

## Results

### Overview of the ARG-Needle method

**Fig. 1:**
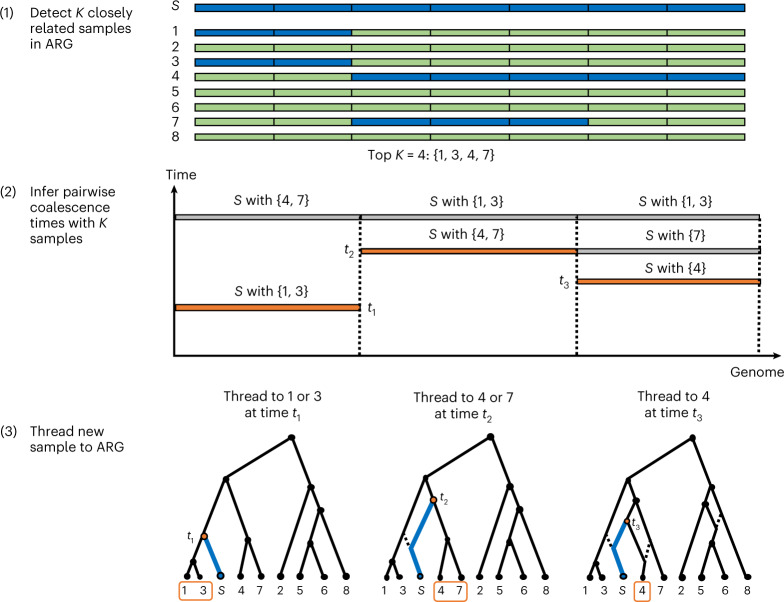
Overview of the ARG-Needle algorithm. ARG-Needle adds one haploid sample at a time to an existing ARG, each time performing three steps: (1) shortlisting a subset of most related samples already in the ARG through genotype hashing, (2) obtaining pairwise coalescence time estimates with these samples using ASMC^11^ and (3) using the ASMC output to ‘thread’^9^ the new sample to the ARG. We depict an example of adding sample *S* to an ARG, focusing on one genomic region. Step 1 divides the genome into ‘words’ and checks for identical matches with sample *S*. Based on these matches (shown in blue), samples 1, 3, 4 and 7 are output as the *K* = 4 candidate most related samples already in the ARG. Step 2 computes pairwise coalescence time estimates between sample *S* and each of the samples 1, 3, 4 and 7. The minimum time for each position is highlighted. Step 3 uses these minimum times and samples to define a ‘threading instruction’ that is performed to add sample *S* to the ARG. Threading connects the new sample to the ancestral lineage of each chosen sample at the chosen time. Dotted lines indicate previous ARG edges that are inactive due to recombination. When all samples have been threaded, ARG-Needle performs a final postprocessing step called ARG normalization (Methods).

The ARG is a graph in which nodes represent the genomes of individuals or their ancestors and edges represent genealogical connections (see Supplementary Note 1 for additional details). ARG-Needle infers the ARG for large genotyping array or sequencing datasets by iteratively ‘threading’^9^ one haploid sample at a time, as depicted in Fig. 1. Given an existing ARG, initialized to contain a single sample, we randomly select the next sample to be added (or threaded). We then compute a ‘threading instruction’, which at each genomic position provides the index of a sample in the ARG that is most closely related to the target sample, as well as their time to most recent common ancestor (TMRCA). We use this instruction to thread the target sample to the current ARG, and iterate until all samples have been included.

To compute the threading instruction of a sample, ARG-Needle first performs genotype hashing^24,25^ to rapidly detect a subset of candidate closest relatives within the ARG. It then uses the Ascertained Sequentially Markovian Coalescent (ASMC) algorithm^11^ to estimate the TMRCA between the new sample and each of these individuals at each site, threading to the closest individual. When all samples have been included, ARG-Needle uses a fast postprocessing step, which we call ARG normalization, to refine the estimated node times. ARG-Needle builds the ARG in time approximately linear in sample size (see below).

We also introduce a simple extension of ASMC^11^, called ASMC-clust, that builds genome-wide genealogies by forming a tree at each site using hierarchical clustering on pairwise TMRCAs output by ASMC. This approach scales quadratically with sample size but yields improved accuracy compared with ARG-Needle in certain simulated scenarios (see below). ARG-Needle and ASMC-clust efficiently represent and store ARGs using a graph data structure, which is an adaptation of the representation used within the ARGON simulator^26^. Additional details, theoretical guarantees and properties for the ARG-Needle and ASMC-clust algorithms are described in the Methods and Supplementary Note 1.

### Accuracy of ARG reconstruction in simulated data

**Extended Data Fig. 1:**
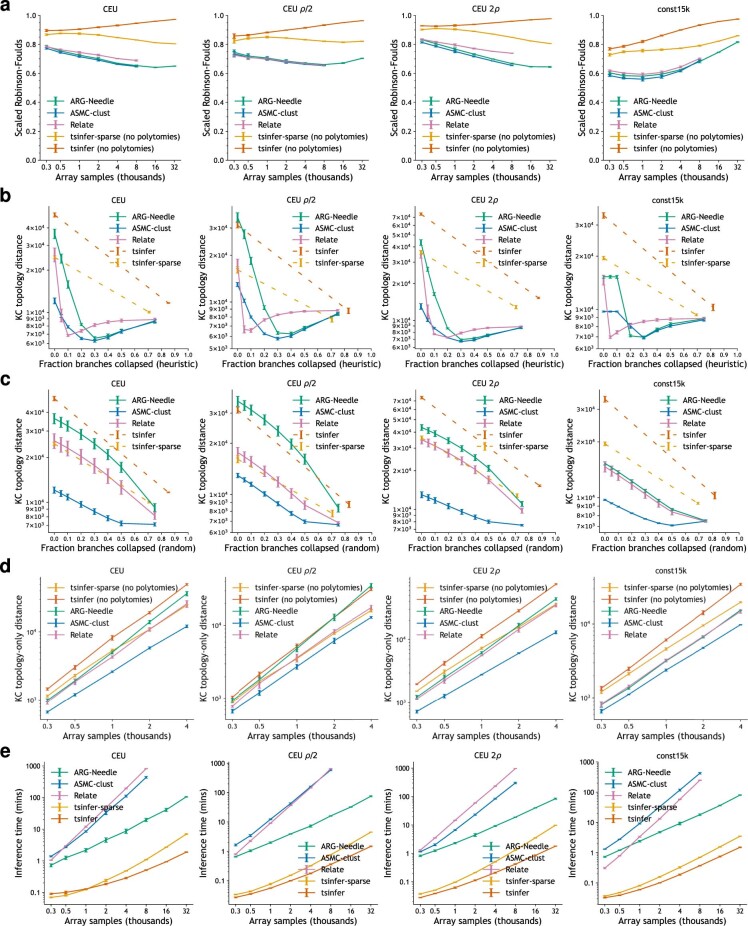
Additional comparison of ARG inference methods with array data and topology-only metrics. We compare methods on runtime and topology-only metrics, as in Fig. 2 but with additional simulation conditions. All columns are for 5 Mb of CEU demography array data, and individual columns represent standard parameters (see Methods), a factor of 2 smaller recombination rate (*ρ* = 6 × 10^−9^), a factor of 2 larger recombination rate (*ρ* = 2.4 × 10^−8^), and a constant population size demography of 15,000 individuals. **a**. Robinson-Foulds distance as a function of the number of samples *N*, where values are scaled to lie between 0 and 1 (polytomies are randomly resolved). **b**. KC topology-only distance for *N* = 4,000 samples, showing performance as branches in marginal inferred trees are collapsed to form polytomies, using a heuristic to preferentially collapse branches that are least certain (see Methods). For tsinfer and tsinfer-sparse, we instead rely on the default amount of polytomies in the output, additionally showcasing when polytomies are randomly resolved (dashed lines indicate a linear trend may not hold). **c**. The same as **b**, except branches are randomly collapsed to form polytomies. **d**. KC topology-only distance as a function of *N*, with polytomies randomly resolved. **e**. Inference time as a function of *N*. All panels use 5 random seeds. Data are presented as means ± 2 s.e.m.

We used extensive simulations to compare the accuracy and scalability of ARG-Needle, ASMC-clust, Relate^17^, tsinfer and a variant of tsinfer designed for sparse datasets we refer to as ‘tsinfer-sparse’^15^. We considered several metrics to compare ARGs, including: the Robinson–Foulds distance^27^, which reflects dissimilarities between the mutations that may be generated by two ARGs; the root mean squared error (RMSE) between true and inferred pairwise TMRCAs, which captures the accuracy in predicting allele sharing between individuals; and the Kendall–Colijn (KC) topology-only distance^28^. We found that the KC distance is systematically lower for trees containing polytomies (that is, nodes with more than two children), which are not output by Relate, ASMC-clust or ARG-Needle (Extended Data Fig. 1b,c). We therefore applied a heuristic to allow these methods to output polytomies (see the Methods and Supplementary Note 2 for additional discussion). Although these three metrics capture similarity between marginal trees, they are not specifically developed for comparing ARGs. We therefore developed an additional metric, called the ARG total variation distance, which generalizes the Robinson–Foulds distance to better capture the ability of a reconstructed ARG to predict mutation patterns that may be generated by the true underlying ARG (see the Methods and Supplementary Note 2 for further details).

**Fig. 2:**
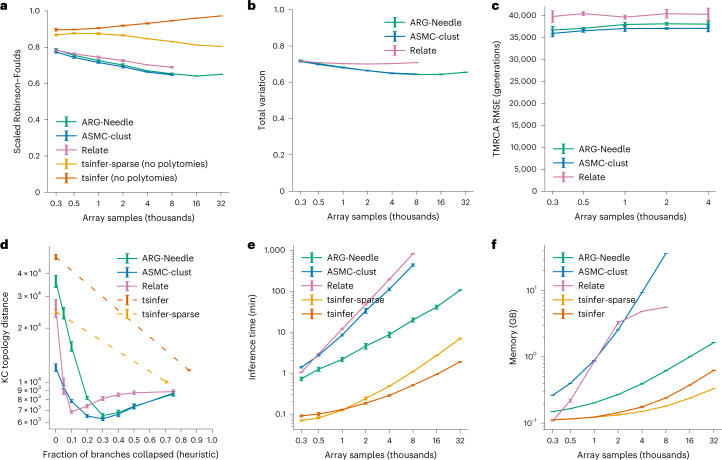
Comparison of ARG inference algorithms in simulation. **a**–**f**, We benchmark ARG inference performance for ARG-Needle, ASMC-clust, Relate, tsinfer and a variation of tsinfer for sparse data (‘tsinfer-sparse’) in realistic CEU demography array data simulations across a variety of metrics related to accuracy and computational resources (lower values indicate better performance for all metrics). **a**, The Robinson–Foulds distance (polytomies are randomly resolved). **b**, The ARG total variation distance (Methods). **c**, Pairwise TMRCA RMSE. **d**, The KC topology-only metric. **e**, Runtime. **f**, Peak memory. In **c**, we only run up to *N* = 4,000 haploid samples. In **d**, we fix *N* = 4,000 haploid samples and vary the fraction of branches per marginal tree that are collapsed to form polytomies, using a heuristic that preferentially collapses branches that are less confidently inferred (Methods). For tsinfer and tsinfer-sparse, we instead rely on the default amount of polytomies in the output, additionally showcasing when polytomies are randomly resolved (dashed lines indicate a linear trend that may not hold). All panels use five random seeds, with ASMC-clust and Relate capped at *N* = 8,000 haploid samples due to runtime or memory constraints. Data are presented as mean values ± 2 s.e.m. Relate’s default settings cap the memory for intermediate computations at 5 GB (see **f**). ARG-Needle and ASMC-clust include ARG normalization by default (Methods), while Relate does not. For additional simulations, see Extended Data Figs. 1–4 and Supplementary Figs. 1–6.

**Extended Data Fig. 2:**
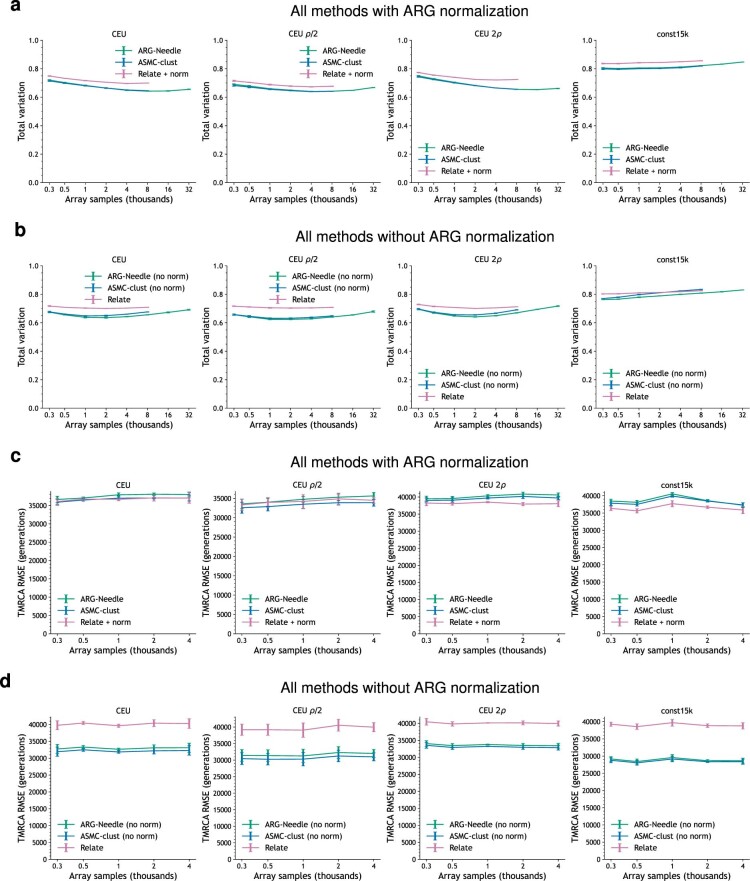
Additional comparison of ARG inference methods with array data and two branch length-aware metrics. We compare methods as in Fig. 2b, c, but with additional simulation conditions. All columns are for 5 Mb of CEU demography array data, and individual columns represent standard parameters (see Methods), a factor of 2 smaller recombination rate (*ρ* = 6 × 10^−9^), a factor of 2 larger recombination rate (*ρ* = 2.4 × 10^−8^), and a constant population size demography of 15,000 individuals. We show results for the ARG total variation distance (**a-b**) and pairwise TMRCA RMSE (**c-d**), with (**a,c**) and without (**b,d**) ARG normalization, as these metrics are sensitive to branch length. All panels use 5 random seeds. Data are presented as means ± 2 s.e.m.

**Extended Data Fig. 3:**
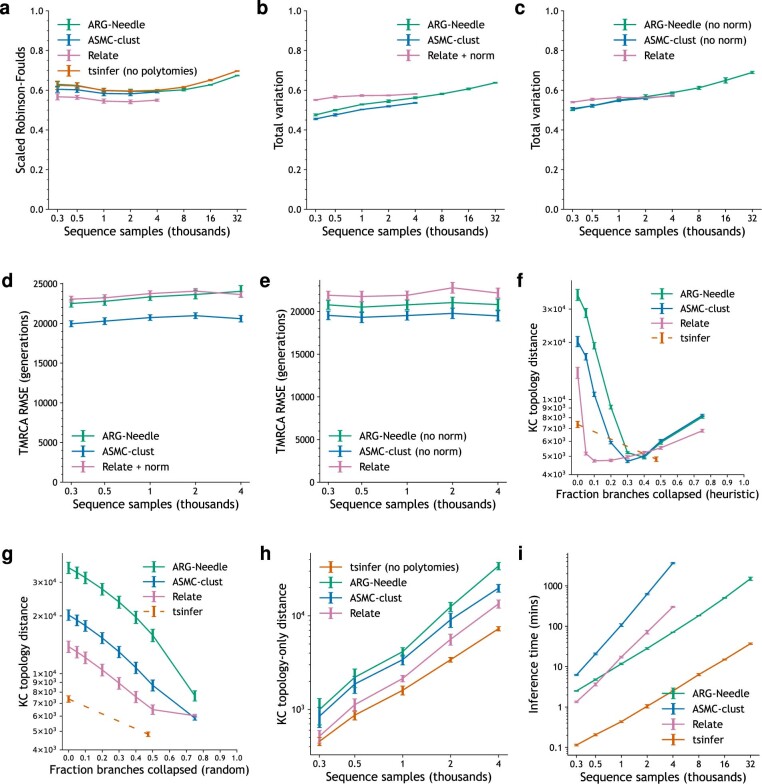
Comparison of ARG inference methods with sequencing data. Simulations use 1 Mb of CEU sequencing data and otherwise standard parameters (see Methods). Individual panels correspond to rows of Extended Data Figs. 1a, 2a–d, and 1b–e, in that order, with the same metrics used, namely **a**. scaled Robinson-Foulds distance (polytomies are randomly resolved), **b-c**. ARG total variation distance with (**b**) and without (**c**) ARG normalization, **d-e**. pairwise TMRCA RMSE with (**d**) and without (**e**) ARG normalization, **f-g**. KC topology-only distance for *N* = 4,000 samples with heuristic (**f**) and random (**g**) collapsing of branches, **h**. KC topology-only distance with polytomies randomly resolved, and **i**. inference time. **d,e** use 25 random seeds, whereas all other panels use 5 random seeds. Data are presented as means ± 2 s.e.m.

We measured ARG reconstruction accuracy in synthetic array datasets of up to 32,000 haploid samples (Fig. 2 and Methods). We also tested a variety of additional conditions, including different demographic histories, varying recombination rates and genotyping error. We also examined the effects of ARG normalization, of variations of the KC distance that account for branch lengths and of stratifying the total variation distance by allele frequency (Extended Data Figs. 1 and 2 and Supplementary Figs. 1–4). ARG-Needle tended to achieve best performance across all accuracy metrics in array data, sometimes tied or in close performance with ASMC-clust or Relate. In simulations of sequencing data, ASMC-clust performed best on the ARG total variation and TMRCA RMSE metrics, with ARG-Needle and Relate close in performance, while Relate performed best on the Robinson–Foulds metric (Extended Data Fig. 3). We next measured the speed and memory footprint of these methods. ARG-Needle requires lower computation and memory than Relate and ASMC-clust, which both scale quadratically with sample size (Fig. 2e,f and Extended Data Fig. 1e). It runs slower than tsinfer and tsinfer-sparse but with a similar (approximately linear) scaling (also see the Methods and Supplementary Note 1).

**Extended Data Fig. 4:**
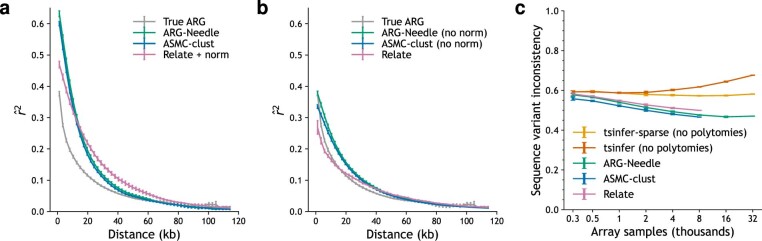
Consistency of inferred ARGs with underlying linkage patterns and sequence-level variation. **a**,**b**. Linkage disequilibrium (LD) decay up to 120 kilobases for ground truth ARGs as well as ARGs inferred by ARG-Needle, ASMC-clust, and Relate. LD was evaluated by placing mutations with a mutation rate of 5 × 10^−8^ per base pair per generation and filtering to variants with MAF > 5%. Lines show mean *r*^2^ as a function of distance between variants, averaging across 10 independent simulations. Simulations were of 5 Mb of CEU demography array data with standard simulation parameters (see Methods). Methods including ARG normalization are shown in **a**, and methods without ARG normalization are shown in **b**, as branch lengths affect the probability for mutations to be sampled. **c**. We compute the fraction of underlying sequencing sites, of which the array variants are a subset, that cannot be mapped to branches of inferred ARGs (lower is better). Inference is on 5 Mb of CEU demography array data simulated with standard parameters (see Methods), averaging over 5 random seeds. no polytomies refers to randomly resolving polytomies of tsinfer and tsinfer-sparse (see Methods). Data are presented as means ± 2 s.e.m.

We next examined additional properties of the ARG-Needle and ASMC-clust algorithms. We found that the order used to thread samples into the ARG does not substantially affect accuracy (Supplementary Fig. 5a–d), but that averaging estimates obtained using different random threading orders may produce improved estimates of genealogical relationships and higher similarity to ARGs inferred using ASMC-clust (Supplementary Fig. 5c–f). We observed that inferred genealogies contain realistic linkage disequilibrium (LD) patterns (Extended Data Fig. 4a,b). ARG-Needle, ASMC-clust and Relate do not guarantee that the variants used to infer genealogies may be mapped to inferred marginal trees, but performed well when we considered the fraction of unobserved variants that could be mapped back to inferred genealogies (Extended Data Fig. 4c; also see ref. ^17^). Finally, we assessed the similarity of ARGs inferred using different algorithms, observing highest similarities between ASMC-clust and ARG-Needle, as well as between these methods and, in decreasing order, Relate, tsinfer-sparse and tsinfer (Supplementary Fig. 6a,b).

### A genealogical approach to LMM analysis

**Fig. 3:**
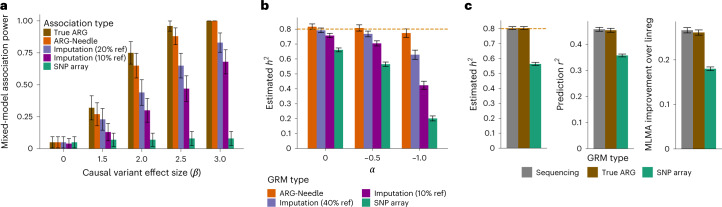
ARG-based analysis of simulated complex traits. **a**, Power to detect a rare causal variant (MAF = 0.025%) in simulations of a polygenic phenotype. We compare ARG-MLMA of ground-truth ARGs and ARG-Needle-inferred ARGs with MLMA of imputed and SNP array variants as we vary the effect size *β* (100 independent simulations of *h*^2^ = 0.8, *α* = −0.25, *N* = 20,000 haploid samples and 22 chromosomes of 5 Mb each; Methods). **b**, Heritability estimation using ARG-GRMs from ARG-Needle inference on SNP array data, compared with using imputed or array SNPs (5 simulations of 25 Mb, *N* = 5,000 haploid samples, *h*^2^ = 0.8 and varying *α*). **c**, ARG-GRMs computed using ground-truth ARGs perform equivalently to GRMs computed using sequencing data in heritability estimation, polygenic prediction and mixed-model association (*N* = 10,000 haploid samples, *h*^2^ = 0.8 and *α* = −0.5). Heritability and prediction involve 5 simulations of 50 Mb, and association involves 50 simulations of 22 chromosomes of 2.5 Mb each, for a total of 55 Mb. For association, we show the relative improvement in mean −log_10_(*P*) of MLMA compared with linear regression (Methods). ‘% ref’ indicates the size of the reference panel used for imputation as a percentage of the number of haploid samples (*N* = 20,000 in **a**, *N* = 5,000 in **b**). Data are presented as estimates ± 2 s.e.m., where the estimates are from meta-analysis in the case of heritability estimation, represent fractions in **a** and represent means otherwise. Additional results are shown in Extended Data Figs. 6–8 and Supplementary Fig. 8. linreg, linear regression.

**Extended Data Fig. 5:**
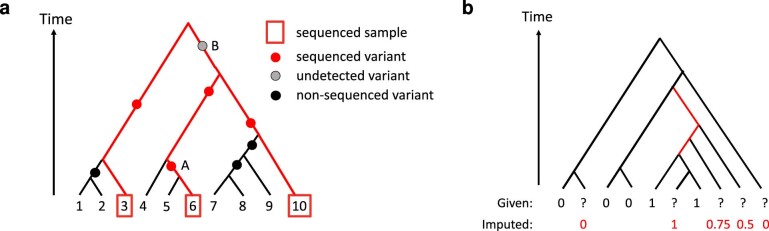
A genealogical view of genotype imputation and an algorithm for ARG-based imputation. **a**. The marginal tree represents the relationships of 10 haploid samples and variant ages at a locus. 3 of the 10 samples are sequenced and used as a reference panel to impute sequenced variants into the remaining samples. An imputation algorithm may recognize sample 6 as the closest relative in the reference panel for samples 4 and 5, but if TMRCAs and variant times are not modeled, it may incorrectly impute variant ‘A’ into sample 4. Variant ‘B’ may represent a high frequency variant that is not present in the sequencing panel (for example, an undetected indel or structural variant). Non-sequenced variants cannot be imputed. All variants may be tested for association using branches of an accurately inferred genealogy. **b**. Schematic of an ARG-based imputation algorithm (see Supplementary Fig. 12 for exploratory results). Given a polymorphic sequenced site containing sequenced samples, unobserved genotypes for array samples, and a marginal tree relating all samples, we perform genotype imputation as follows. We first identify all branches in the tree for which a mutation on that branch best explains the observed sequencing data in terms of Hamming distance (red branches in the example). Each branch implies genotypes of 0 or 1 for the array samples, and we weight by branch length to produce a weighted predicted dosage for each array sample. In this example, the three branches have lengths in ratio 1:1:2, resulting in the predicted dosages shown in red.

**Extended Data Fig. 6:**
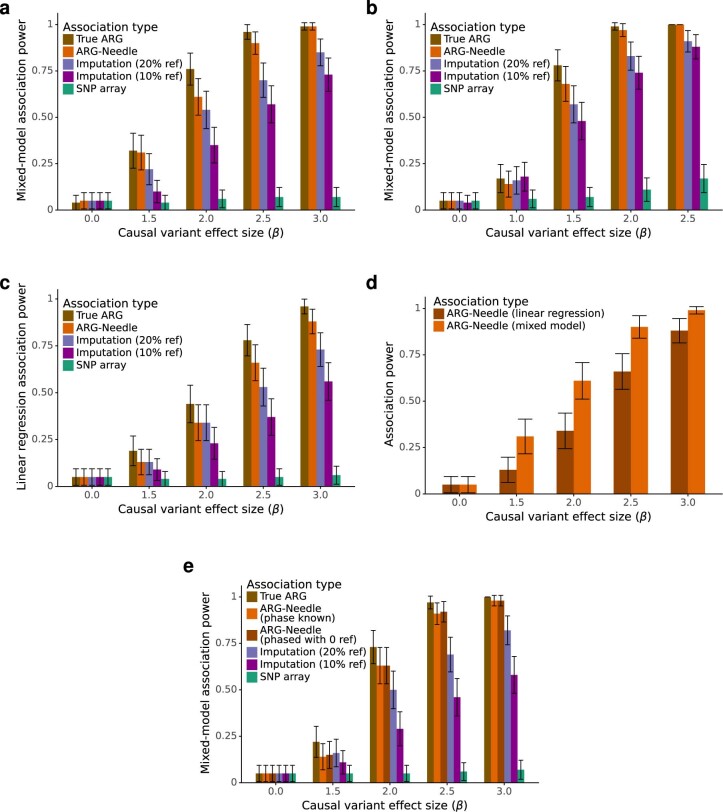
Additional simulations of ARG-MLMA genealogy-wide association power. **a**. Similar to Fig. 3a, except with a low-frequency causal variant (MAF = 0.05%) and a smaller simulation with *N* = 10,000 haploid samples and 22 chromosomes of 2.5 Mb each. **b**. Similar to **a**, except with the causal variant MAF chosen to be 0.1%. **c**. Similar to **a**, except using linear regression instead of the linear mixed model to test for association. **d**. We combine the association power results of ARG-Needle association from **a** and **c**, highlighting the improvement of ARG-MLMA compared to directly testing ARG clades using linear regression. **e**. As in **a**, but with *N* = 10,000 diploid instead of *N* = 10,000 haploid individuals. ARG-Needle is run with the true phase known and with reference-free phasing. *% ref* indicates the size of the reference panel used for imputation as a percentage of the number of haploid samples (*N* = 10,000 in **a-c**, 2 *N* = 20,000 in **e**). All panels use 100 independent simulations to measure power. Data are presented as fractions ± 2 s.e.m.

LMMs enable state-of-the-art analysis of polygenic traits^20,29,30,31^. We developed an approach that uses the ARG of a set of genomes to perform mixed linear model association (MLMA^29^; Methods). More in detail, we use an ARG built from genotyping array data to infer the presence of unobserved variants and perform MLMA testing of these variants. This increases association power in two ways: the ARG is used to uncover putatively associated variants, while the LMM utilizes estimates of genomic similarity to model polygenicity, relatedness and population stratification^29^. We refer to association analyses that test variants in the ARG as ‘genealogy-wide association’ scans and, more specifically, to analyses that incorporate mixed linear model testing as ARG-MLMA. Genealogy-wide association complements genotype imputation based on a sequenced reference panel, as it enables capturing rare variants in the sample that may be absent from the panel or cannot be accurately imputed (Extended Data Fig. 5a). It also generalizes rare variant association strategies based on haplotype sharing^13,19,25,32–35^, as detailed in Supplementary Fig. 7. In simulations, we observed that for low-frequency variants genealogy-wide association may achieve higher association power than testing of variants imputed from a sequenced reference panel (Fig. 3a and Extended Data Fig. 6).

**Extended Data Fig. 7:**
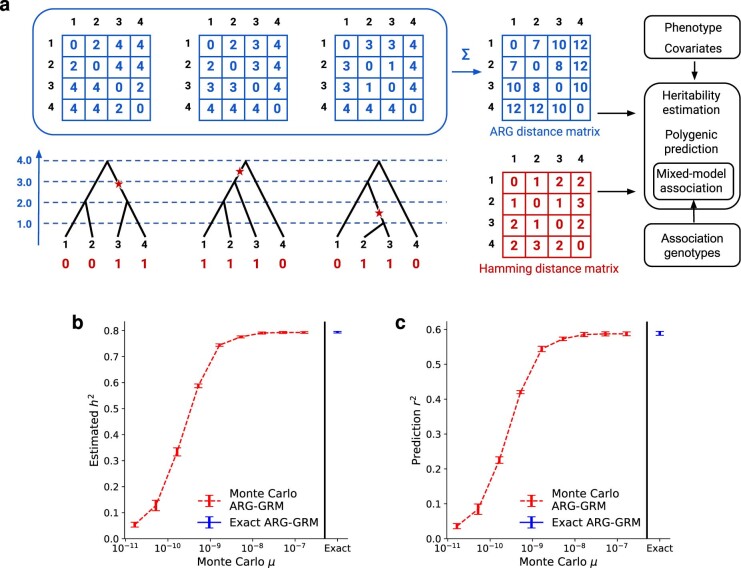
Overview of ARG-GRM definition and Monte Carlo estimator. **a**. Schematic of ARG-GRMs. Given an ARG between samples, we can compute the TMRCA matrix at each site and sum this over the genome to obtain the *α* = 0 ARG distance matrix (top, in blue). This equals a scaled version of the expected Hamming distance matrix (bottom, in red), which is formed by counting the number of differences between the genotypes of samples. By applying a series of simple matrix transformations to the ARG distance matrix (see Supplementary Note 3), we obtain the ARG-GRM, which can subsequently be used in complex trait analysis just like genotype-based GRMs. **b,c**. We compare the use of an exact *α* = 0 ARG-GRM to Monte Carlo *α* = 0 ARG-GRMs for heritability estimation (**b**) and polygenic prediction (**c**). As we increase the mutation rate for the Monte Carlo ARG-GRMs (rightmost value of *μ* = 1.65 × 10^−7^), we approach results from using the exact ARG-GRM. Shown are 5 independent simulations of *N* = 2,000 haploid samples, *h*^2^ = 0.8, *α* = 0, 10 Mb. Data are presented as estimates ± 2 s.e.m., where the estimates are from meta-analysis in the case of heritability estimation and represent means otherwise.

**Extended Data Fig. 8:**
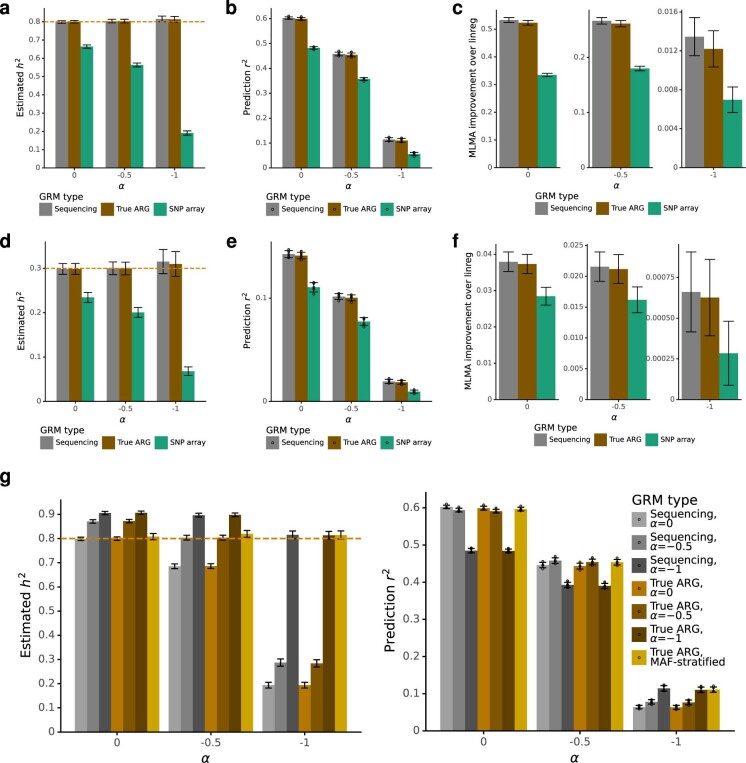
Additional simulations for ground-truth ARG-GRMs. **a-f**. As in Fig. 3c, with *N* = 10,000 haploid samples, except we vary *h*^2^ ∈ {0.8, 0.3} and *α* ∈ {0, −0.5, −1}. **a, d**. Heritability estimation for a 50 Mb region for *h*^2^ = 0.8 (**a**) and *h*^2^ = 0.3 (**d**). **b, e**. Polygenic prediction for a 50 Mb region for *h*^2^ = 0.8 (**b**) and *h*^2^ = 0.3 (**e**). **c, f**. Mixed-model association for 22 chromosomes of 2.5 Mb each for *h*^2^ = 0.8 (**c**) and *h*^2^ = 0.3 (**f**). **g**. Panels **a-f** assumed it is possible to infer *α* and used the true *α* when building genotype-based or ARG-GRMs. If this value of *α* is misspecified, heritability estimation is biased and prediction *r*^2^ is hampered. This is true both for ARG-GRMs and sequencing GRMs. However, using MAF-stratified ARG-GRMs provides a robust way to estimate the true heritability when *α* is unknown, and achieves prediction *r*^2^ comparable to using the true *α* (*N* = 10,000 haploid samples, 50 Mb, *h*^2^ = 0.8). For all panels, heritability and prediction experiments involve 5 simulations per bar, and most association experiments involve 50 simulations per bar, except for the *h*^2^ = 0.3, *α* = −1 condition in **f**, which involved 500 simulations. Data are presented as estimates ± 2 s.e.m., where the estimates are from meta-analysis in the case of heritability estimation and represent means otherwise. Prediction *r*^2^ for individual simulations is shown in **b** and **e**.

In addition, we developed strategies to leverage the ARG to obtain estimates of genomic similarity across individuals, which are aggregated in a genomic relatedness matrix (GRM; Methods) and are a key element of several mixed-model analyses of complex traits. We refer to GRMs built using this approach as ARG-GRMs and provide details of their construction and properties in Supplementary Note 3. We used ARG-GRMs to measure the amount of phenotypic variance captured by inferred ARGs (Extended Data Fig. 7a). In simulations, ARG-GRMs built using ARGs inferred by ARG-Needle in array data captured more narrow sense heritability than GRMs built using array data^30,36,37^ (Methods, Fig. 3b and Supplementary Fig. 8). We also performed additional simulations to test whether the modeling of unobserved genomic variation using ARG-GRMs may be leveraged to obtain performance gains in other LMM analyses. Indeed, ARG-GRMs built using true ARGs performed as well as GRMs computed using sequencing data in LMM-based heritability estimation, polygenic prediction and association (Methods, Fig. 3c and Extended Data Fig. 8). Applying these strategies to large-scale inferred ARGs, however, will require improved accuracy and scalability (Discussion).

Overall, these experiments suggest that accurate genealogical inference combined with LMMs improves association power, by testing variants that are not well tagged using available markers while modeling polygenicity. The ARG may also be potentially utilized to obtain improved estimates of genomic similarity and perform additional LMM-based complex trait analyses.

### Genealogy-wide association scan of rare and ultra-rare variants in the UK Biobank

We applied ARG inference methods in a subset of the genome using UK Biobank data and observed results consistent with our simulations (Supplementary Fig. 6c,d). We then used ARG-Needle to build the genome-wide ARG from SNP array data for 337,464 individuals in the white British ancestry subset defined by ref. ^38^ (Methods). We performed ARG-MLMA for height and six molecular traits, comprising alkaline phosphatase, aspartate aminotransferase, low-density lipoprotein (LDL) / high-density lipoprotein (HDL) cholesterol, mean platelet volume and total bilirubin. To achieve the required scalability, we built on a recent MLMA method^22,39^, implicitly relying on an array-based GRM (Methods and Discussion). We compared ARG-MLMA with standard MLMA testing of variants imputed using the Haplotype Reference Consortium (HRC) and UK10K reference panels^38,40,41^ (hereafter, HRC + UK10K), comprising ~65,000 haplotypes. We focused on rare (0.01% ≤ MAF < 0.1%) and ultra-rare (MAF < 0.01%) genomic variants. We used resampling-based testing^42^ to establish genome-wide significance thresholds of *P* < 4.8 × 10^−11^ for ARG variants (sampled with mutation rate *μ* = 10^−5^) and *P* < 1.06 × 10^−9^ for imputed variants (Supplementary Table 1). For each analysis, we performed LD-based filtering to extract a stringent set of approximately independent associations (hereafter, ‘independent associations’; Methods). We leveraged a subset of 138,039 individuals with whole-exome sequencing (WES) data (hereafter, WES-138K) to validate these independent associations. For each detected independent variant, we selected the WES variant with the largest correlation, which we call its ‘WES partner’.

**Fig. 4:**
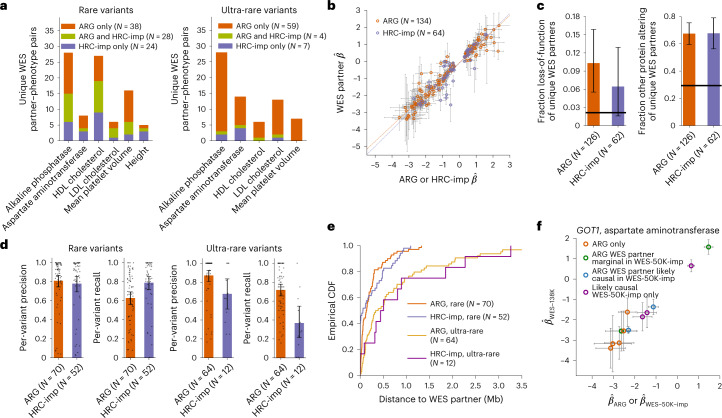
Association of ARG-derived and imputed rare and ultra-rare variants with seven quantitative traits in UK Biobank. **a**, Counts of unique WES partners for ARG and HRC + UK10K-imputed (‘HRC-imp’) independent associations, partitioned by traits and frequency and showing overlap. Total bilirubin was not associated at these frequencies and height was not associated for ultra-rare variants. **b**, Scatter plot of $\hat \beta$ (estimated effect) for independent variants (estimated within 337,464 samples) against $\hat \beta$ for their WES partners (estimated within 138,039 samples), with linear model fit. **c**, Fraction of loss-of-function and other protein-altering variants for the unique WES partners of independent variants (125 WES partners for ARG and 62 for imputed variants). Horizontal black lines represent averages across exome sequencing variants. **d**, Average per-variant precision and recall of predicting WES carrier status, partitioned by frequency and showing individual value as jittered points (71 rare ARG, 53 rare imputed, 62 ultra-rare ARG and 12 ultra-rare imputed variants). **e**, Cumulative distribution function (CDF) for the distance between independent variants and their WES partners. **f**, Scatter plot of $\hat \beta$ for ARG-derived independent variants associated with aspartate aminotransferase in the *GOT1* gene (estimated within 337,464 samples) against $\hat \beta$ for their WES partners (estimated within 138,039 samples). We color points based on whether the WES partner is likely causal in WES-50K-imp (imputation from WES-50K into ~459,000 samples^43^), not likely causal but marginally significant in WES-50K-imp or not marginally significant in WES-50K-imp (‘ARG only’). We also plot the $\hat \beta$ for the additional likely causal variants in WES-50K-imp against the $\hat \beta$ in WES-138K. Bars represent fractions in **c** and means in **d**. Error bars represent 1.96 s.e.m. in **b** and **f** and represent bootstrap 95% CIs in **c** and **d**. Additional results are shown in Extended Data Fig. 9. HDL, high-density lipoprotein; LDL, low-density lipoprotein.

**Extended Data Fig. 9:**
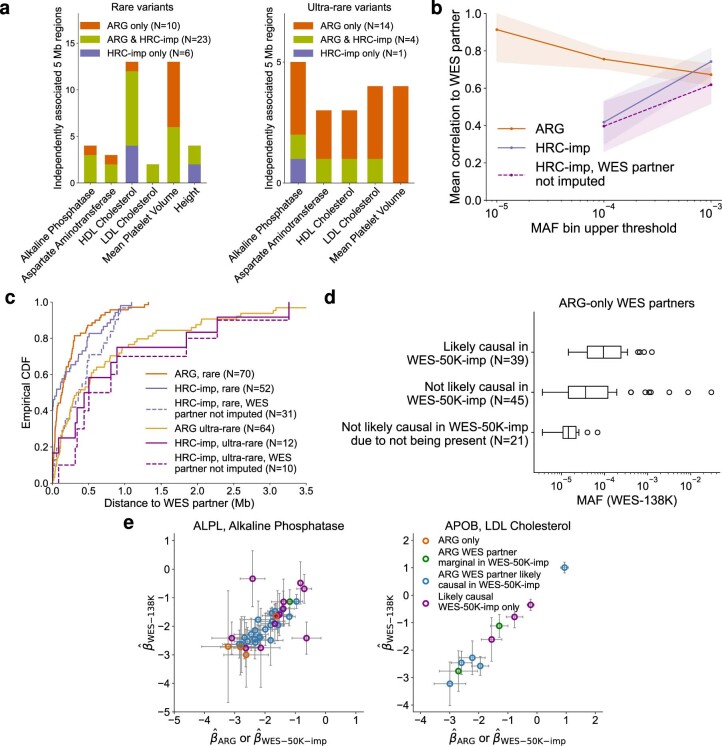
Further results for rare and ultra-rare variant associations. **a**. Counts of implicated 5 Mb regions containing ARG and HRC + UK10K imputation (‘HRC-imp’) independent associations, partitioned by traits and frequency and showing overlap. Total bilirubin was not associated at these frequencies. **b**. Average Pearson correlation between independent variants and their WES partners as a function of frequency, for ARG-derived variants, HRC + UK10K imputed variants, and HRC + UK10K imputed variants for which the WES partner was not the imputed variant. Dots represent the upper end of a frequency range. Central lines represent means and shaded areas represent 95% bootstrap confidence intervals. **c**. Cumulative distribution function for the distance between independent variants and their WES partners, partitioned by frequency. As in Fig. 4b, but also showing HRC + UK10K imputed variants for which the WES partner was not the imputed variant. **d**. Box plots of MAF for WES partners found by ARG-derived but not HRC + UK10K imputed independent variants (center line, median; box limits, upper and lower quartiles, whiskers, 1.5× interquartile range; points, outliers), stratifying by status in WES-50K-imp (imputation from WES-50K). **e**. Scatter plot of $\hat \beta$ (estimated effect) for ARG-derived independent variants (estimated within 337,464 samples) against $\hat \beta$ for their WES partners (estimated within 138,039 samples), as in Fig. 4f but for associations with alkaline phosphatase in the *ALPL* gene and with LDL cholesterol in the *APOB* gene. We color points based on whether the WES partner is likely causal in WES-50K-imp, not likely causal but marginally significant in WES-50K-imp, or not marginally significant in WES-50K-imp (‘ARG only’ in figure). We also plot the $\hat \beta$ for the additional likely causal variants in WES-50K-imp against the $\hat \beta$ in WES-138K. Error bars represent 1.96 s.e.m.

Applying this approach, we detected 134 independent signals using the ARG and 64 using imputation, jointly implicating 152 unique WES partners (Supplementary Tables 2 and 3). Of these WES variants, 36 were implicated using both approaches (Fig. 4a, and see Extended Data Fig. 9a for region-level results). The fraction of WES partners uniquely identified using the ARG was larger among ultra-rare variants (84%) compared with rare variants (42%), reflecting a scarcity of ultra-rare variants in the HRC + UK10K imputation panel. The phenotypic effects estimated in the 337,464 individuals using ARG-derived or imputed associations were strongly correlated to those directly estimated for the WES partners in the WES-138K dataset (Fig. 4b), with stronger average correlation (bootstrap *P* = 0.003) for ARG-derived variants ($r_{\mathrm{ARG}}^2$ = 0.93) compared with imputed variants ($r_{\mathrm{imp}}^2$ = 0.80). Only 74% of the WES partners for ARG-derived rare variant associations were significantly associated (*P* < 5 × 10^−8^) in the smaller WES-138K dataset, a proportion that dropped to 59% for ultra-rare variants. Variants detected using genealogy-wide association had a larger average phenotypic effect than those detected via imputation (bootstrap *P* < 0.0001; average $\left| {\hat \beta _{\mathrm{ARG}}} \right|$ = 1.46; average $\left| {\hat \beta _{\mathrm{imp}}} \right|$ = 0.90), reflecting lower average frequencies. In addition, WES partners of ARG-derived variants were ~4.8× enriched for loss-of-function variation (bootstrap *P* < 0.001; Fig. 4c), and WES partners implicated by either ARG or imputation were ~2.3× enriched for other protein-altering variation (Methods), supporting their likely causal role.

We also used variant-level precision and recall statistics (Methods) to measure the extent to which carrying an associated ARG-derived or imputed variant is predictive of carrying sequence-level WES partner variants (Fig. 4d). ARG-derived and imputed rare variants had similar levels of variant-level precision, while imputation had higher recall (bootstrap *P* = 0.0005). For ultra-rare variants, ARG-derived signals performed better than imputed variants for both precision (bootstrap *P* = 0.01) and recall (bootstrap *P* = 0.002). Similarly, ARG-derived and imputed rare variants provided comparable tagging for their WES partners (Extended Data Fig. 9b), while ARG-derived ultra-rare variants provided stronger tagging compared with imputed ultra-rare variants (average *r*_ARG_ = 0.77, *r*_imp_ = 0.42, bootstrap *P* < 0.001). Compared with ARG-derived variants, genotype imputation has the advantage that associated variants that are sequenced in the reference panel may be directly localized in the genome. We found that for 21 of 52 rare and 2 of 12 ultra-rare independent imputation signals the WES partner had been imputed, while the remaining signals likely provide indirect tagging for underlying variants. ARG-derived and imputed variants, however, had similar distributions for the distance to their WES partners (Fig. 4e and Extended Data Fig. 9c). This suggests that genealogy-wide associations have the same spatial resolution as associations obtained using genotype imputation in cases where the variant driving the signal cannot be directly imputed.

We compared our results with those of a recent study that leveraged exome sequencing data from a subset of ~50,000 participants (hereafter, WES-50K) to perform genotype imputation for ~459,000 samples^43^. We found that, among the WES partners implicated using the ARG but not using HRC + UK10K imputation, 14 of 30 partners of rare and 26 of 55 partners of ultra-rare ARG variants were also flagged as likely causal associations (*P* < 5 × 10^−8^) in ref. ^43^ (Supplementary Table 2). The remaining 45 WES partners detected using the ARG but not reported in ref. ^43^ are often very rare variants (median MAF = 3.6 × 10^−5^; Extended Data Fig. 9d) of large effect (median |$\hat \beta$| = 1.14), which are difficult to impute; 21 of 45 such variants were absent or singletons in the WES-50K reference panel or had poor imputation quality score. Associations uniquely detected using the ARG often extended allelic series at known genes. For instance, restricting to loss-of-function or other protein-altering WES partners for independent ARG signals not present or marginally significant in ref. ^43^, five novel associations with aspartate aminotransferase are mapped to the *GOT1* gene (Fig. 4f) and four with alkaline phosphatase are mapped to *ALPL* (Extended Data Fig. 9e). A subset of strong independent associations uniquely detected by the ARG had weak correlation with their WES partners, possibly due to tagging of structural or regulatory variation absent from the WES-138K dataset (for example, a signal for aspartate aminotransferase with *P* = 7.4 × 10^−39^, MAF_ARG_ = 0.0005, WES partner *r* = 0.21, minor allele count (MAC)_WES-138K_ = 6, MAC_WES-50K_ = 1).

In summary, genealogy-wide association using an ARG inferred from common SNPs revealed more rare and ultra-rare signals than genotype imputation based on ~65,000 reference haplotypes, and detected ultra-rare variants that were not associated using within-cohort imputation based on ~50,000 exome-sequenced participants. ARG-derived associations accurately predicted effect sizes for underlying sequencing variants, as well as the subset of carrier individuals.

### Genealogy-wide association for low- and high-frequency variants

**Extended Data Fig. 10:**
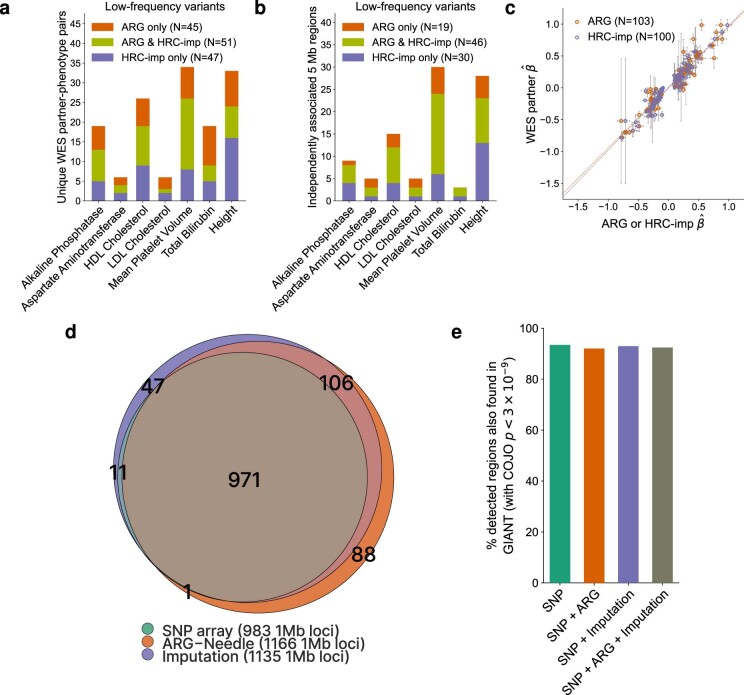
Additional results for low (0.1% ≤ MAF < 1%) and high frequency (MAF ≥ 1%) variant associations. **a-c**. Association of ARG-derived and imputed low-frequency variants with 7 quantitative traits. **a**. Counts of unique WES partners for ARG and HRC + UK10K imputed (‘HRC-imp’) independent associations, partitioned by traits and showing overlap. **b**. Counts of implicated 5 Mb regions containing ARG and HRC + UK10K imputation independent associations, partitioned by traits and showing overlap. **c**. Scatter plot of estimated effect ($\hat \beta$) for independent variants (estimated within 337,464 samples) against $\hat \beta$ for their WES partners (estimated within 138,039 samples), with linear model fit. Error bars represent 1.96 s.e.m. **d**, **e**. Association of higher frequency variants with height. **d**. Venn diagram of number of 1 Mb regions containing a significant hit at *P* < 3 × 10^−9^ for ARG-Needle (MAF ≥ 1%, *μ* = 10^−5^), HRC + UK10K imputed (MAF ≥ 0.1%, info score > 0.3) and SNP array association. ARG-Needle association detected 971 out of 982 (98.9%) 1 Mb regions found by both imputation and array, 108 out of 153 (71%) 1 Mb regions found by imputation but not array and an additional 92 (8% increase upon 1140) 1 Mb regions to those already found by imputation and array. **e**. Percent of 1 Mb regions containing independent associations (defined as having COJO *P* < 3 × 10^−9^, see Methods) in association scans of 337,464 UK Biobank individuals that were also present in a GIANT consortium meta-analysis of ∼700,000 samples.

Lastly, we performed genealogy-wide association for low- (0.1% ≤ MAF < 1%) and high- (MAF ≥ 1%) frequency variants, which are more easily imputed using reference panels that are not necessarily large and population-specific. Consistent with this, extending our previous analysis to low-frequency variants yielded a similar number of independent associations for ARG-derived and HRC + UK10K-imputed variants (*N*_ARG_ = 103, *N*_imp_ = 100; Supplementary Tables 4 and 5 and Extended Data Fig. 10a–c). Associations detected using the ARG had slightly larger effects compared with those found using imputation (bootstrap *P* = 0.026; average |*β*_ARG_| = 0.32, |*β*_imp_| = 0.27) but provided lower tagging to WES partners (bootstrap *P* < 0.001; average *r*_ARG_ = 0.57, *r*_imp_ = 0.73), reflecting the large fraction (42 of 100) of imputation WES partners that were directly imputed.

We hypothesized that, although imputation of higher frequency variants is generally more accurate, branches in the marginal trees of the ARG may in some cases complement available markers by providing improved tagging of underlying variation. This may be the case, for instance, for short insertions/deletions or structural variants^44^, which are often underrepresented in reference panels^41^, or for variants of moderately high frequency, which may be difficult to impute^45^ (Extended Data Fig. 5a). To test this, we performed MLMA for height using HRC + UK10K-imputed variants, filtered as in ref. ^38^ (MAF > 0.1%, info score > 0.3; Methods), for which we established a resampling-based genome-wide significance threshold of 4.5 × 10^−9^ (95% confidence interval (95% CI): 2.2 × 10^−9^, 9.6 × 10^−9^). To facilitate direct comparison, we selected ARG-MLMA parameters (MAF > 1%, *μ* = 10^−5^; Methods) corresponding to a higher MAF cutoff but a comparable genome-wide significance threshold of 3.4 × 10^−9^ (95% CI: 2.4 × 10^−9^, 5 × 10^−9^) and adopted a threshold of 3 × 10^−9^ for all downstream analyses.

**Fig. 5:**
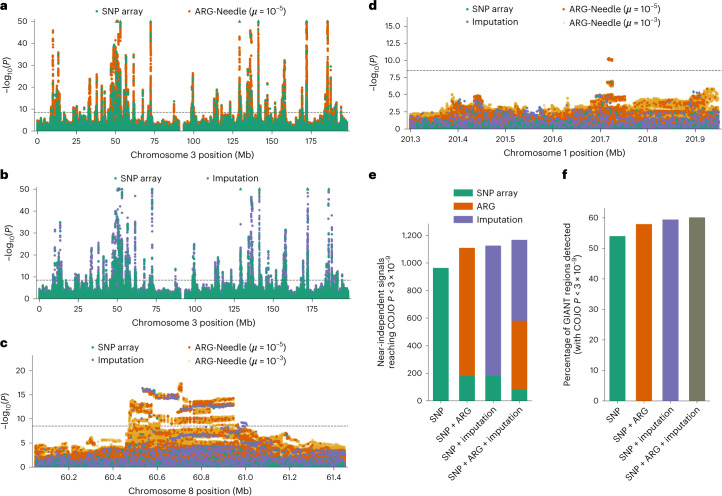
Genealogy-wide association of higher frequency variants with height in UK Biobank. **a**,**b**, Chromosome 3 Manhattan plots showing MLMA of ARG-Needle on SNP array data versus array SNPs (**a**) and HRC + UK10K-imputed variants versus array SNPs (**b**). **c**,**d**, Manhattan plots of two loci. **c**, ARG-MLMA detects haplotype structure that is found using imputation, with a different association peak. **d**, An association peak found by ARG-MLMA that was significant (*P* < 3 × 10^−9^) in a GIANT consortium meta-analysis of ~700,000 samples. **e**,**f**, Approximately independent associations (defined as having COJO *P* < 3 × 10^−9^; Methods) when considering array SNPs alone, array SNPs and ARG-Needle variants, array SNPs and imputed variants, and all three types of variants. **e**, Total number of independent variants found and attribution based on data type. **f**, Percentage of 1-Mb regions containing COJO associations in the GIANT meta-analysis that are detected using each method. For the Manhattan plots, the order of plotting is ARG-Needle with *μ* = 10^−3^ (used for follow-up), then ARG-Needle with *μ* = 10^−5^ (used for discovery), then imputation, then SNP array variants on top. Dotted lines correspond to *P* = 3 × 10^−9^ (Methods) and triangles indicate associations with *P* < 10^−50^. See also Supplementary Figs. 9 and 10.

We first assessed the number of 1-megabase (Mb) regions that contain an association (*P* < 3 × 10^−9^) for genotype array, imputed or ARG-derived variants. We found that ARG-MLMA detected 98.9% of regions found by both SNP array and imputation, as well as 71% of regions found by imputation but not array data and an additional 8% of regions not found using either imputation or array data (Extended Data Fig. 10d). A significant fraction (54 of 92, permutation *P* < 0.0001) of regions identified using the ARG but not imputation contained associations (*P* < 3 × 10^−9^) in a larger meta-analysis by the Genetic Investigation of ANthropometric Traits (GIANT) consortium^46^ (*N* ≈ 700,000) comprising the UK Biobank and additional cohorts. Inspecting associated loci, we observed that ARG-MLMA captures association peaks and haplotype structure found using imputation but not array data (Fig. 5a–c and Supplementary Figs. 9 and 10a–e) as well as association peaks uniquely identified using ARG-MLMA (Fig. 5d and Supplementary Fig. 10f–h).

We sought to further assess the degree of overlap and complementarity of associations detected using SNP array data, imputation and the ARG, by performing LD-based filtering and conditional and joint (COJO^47^) association analyses (Fig. 5e and Methods). When we jointly considered either or both ARG-derived and imputed variants in addition to array markers, we observed an increase in the number of approximately independent COJO associations (*P* < 3 × 10^−9^; *N*_SNP_ = 964, *N*_SNP+ARG_ = 1,110, *N*_SNP+imp_ = 1,126, *N*_SNP+ARG+imp_ = 1,161). The fraction of COJO-associated array markers was reduced by the inclusion of ARG-derived or imputed variants, which resulted in comparable proportions of associations when jointly analyzed (Fig. 5e), suggesting that both ARG and imputation provide good tagging of underlying signal. By considering the set of 1-Mb regions harboring significant COJO associations, we verified that the additional COJO signals detected when including ARG-derived or imputed variants concentrated within regions that also harbor significant (*P* < 3 × 10^−9^) COJO signals in the GIANT meta-analysis^46^ (Fig. 5f and Extended Data Fig. 10e).

In summary, genealogy-wide association using the ARG inferred by ARG-Needle from SNP array data was less effective for the analysis of higher frequency variants because these variants could be more accurately imputed compared to rare and ultra-rare variants. However, ARG-derived variants revealed associated peaks and haplotypes that were not found through association of array data alone and in some cases complemented genotype imputation in detecting approximately independent associations. We note that the choices of filtering criteria, such as MAF threshold, imputation info score and ARG mutation rate, all affect the sensitivity and specificity of these analyses. Results for an analysis restricting to association of variants with MAF > 10% are shown in Supplementary Fig. 11.

## Discussion

We developed ARG-Needle, a method for accurately inferring genome-wide genealogies from genomic data that scales to large biobank datasets. We performed extensive simulations, showing that ARG-Needle is both accurate and scalable when applied to genotyping array and sequencing data. We also developed a framework that combines inferred genealogies with LMMs to increase association power, and showed that this strategy may be further leveraged in analyses of heritability and polygenic prediction. We built genome-wide ARGs from genotyping array data for 337,464 UK Biobank individuals and performed a genealogy-wide association scan for seven quantitative phenotypes. Using the inferred ARG, we detected more large-effect associations to rare and ultra-rare variants than using genotype imputation from ~65,000 sequenced haplotypes, down to an allele frequency of ~4 × 10^−6^. We validated these signals using exome sequencing, showing that they tag underlying variants enriched for loss-of-function and other protein-altering variation. Associations detected using the ARG overlap with and extend fine-mapped associations detected using within-cohort genotype imputation. Applied to the analysis of higher frequency variants, the ARG revealed haplotype structure and independent signals complementary to those obtained using imputation.

These results highlight the importance of genealogical modeling in the analysis of complex traits. Genome-wide association analyses rely on the correlation between available markers and underlying variation^48^ and the MLMA framework accounts for polygenicity, relatedness and population structure^29^. In genealogy-wide association, the signal of LD is amplified by further modeling of past recombination events to infer the presence of hidden genomic variation. Through ARG-GRMs, inferred genealogies may facilitate better modeling of genomic similarity and polygenic effects, leading to improved robustness and increased statistical power.

These analyses also demonstrate that genealogical inference provides a complementary strategy to genotype imputation approaches, which rely on haplotype sharing between the analyzed samples and a sequenced reference panel to extend the set of available markers. Imputation has been successfully applied in several complex trait analyses^4,36^, but its efficacy for the study of rare variants hinges on the availability of large, population-specific sequencing panels, which are not widely available for all populations. Genealogy-wide association may therefore offer new avenues to better utilize genomic resources for underrepresented groups^49^.

We highlight several limitations and directions of future development. First, although genealogy-wide association enables detecting individuals carrying associated variants, it may implicate large genomic regions, whereas genotype imputation may associate individual variants if they are sequenced in the reference panel. When sequencing data are available, however, they may be utilized to further localize ARG-derived signals, for instance using WES partners. Second, although we have shown in simulation that ARG-GRMs built from true ARGs may be used to estimate heritability, perform prediction and increase association power, real data applications of this approach will require methodological improvements to increase LMM scalability^50,51^. Third, although our study was restricted to unrelated samples of homogeneous ancestry, we expect genealogy-wide association to be as susceptible as standard association to issues such as relatedness and population stratification^29,52,53^, requiring adequate control for these confounders. Fourth, although we have focused on leveraging an ARG inferred from array data alone, ARG-Needle enables building an ARG using a mixture of sequencing and array data. This approach may be used to perform additional analyses such as ARG-based genotype imputation, which is likely to improve upon approaches that do not model the TMRCA between target and reference samples^54^. In simulations we performed, this ARG-based imputation strategy obtained promising results (Supplementary Note 4, Extended Data Fig. 5 and Supplementary Fig. 12). Fifth, our analyses were limited to quantitative traits; support for MLMA of rare case/control traits will require methodological extensions. Sixth, we adopted a computationally intensive resampling-based approach^42^ to establish significance thresholds across filtering parameters; future work may lead to improved strategies to address multiple testing. Seventh, although we relied on several existing and novel metrics to analyze properties of the reconstructed ARGs, further research should develop additional metrics and explore their properties and relationships to downstream analyses. These metrics should be applicable for benchmarking methods that only infer the topology of an ARG as well as methods that focus on estimating branch lengths^55^. Eighth, reconstructing biobank-scale ARGs will likely aid the study of additional evolutionary properties of disease-associated variants, including analyses of natural selection acting on complex traits^11,56,57^ which we have not explored in this work. Finally, our analysis focused on the UK Biobank dataset, which provides an excellent testbed due to the large volumes of high-quality data of different types available for validation. Future applications of our methods will involve analysis of cohorts that are less strongly represented in current sequencing studies. Nevertheless, we believe that the results described in this work represent an advance in large-scale genealogical inference and provide new tools for the analysis of complex traits.

## Methods

### ARG-Needle and ASMC-clust algorithms

We introduce two algorithms to construct the ARG of a set of samples, called ARG-Needle and ASMC-clust. Both approaches leverage output from the ASMC algorithm^11^, which takes as input a pair of genotyping array or sequencing samples and outputs a posterior distribution of the TMRCA across the genome. ARG-Needle and ASMC-clust use this pairwise genealogical information to assemble the ARG for all individuals.

ASMC-clust runs ASMC on all pairs of samples and performs hierarchical clustering of TMRCA matrices to obtain an ARG. At every site, we apply the unweighted pair group method with arithmetic mean (UPGMA) clustering algorithm^58^ on the *N* × *N* posterior mean TMRCA matrix to yield a marginal tree. We combine these marginal trees into an ARG, using the midpoints between sites’ physical positions to decide when one tree ends and the next begins. Using an *O(N*^2^*)* implementation of UPGMA^59,60^, we achieve a runtime and memory complexity of *O(N*^2^*M)*. We also implement an extension that achieves *O(NM)* memory but increased runtime (Supplementary Note 1).

ARG-Needle starts with an empty ARG and repeats three steps to add additional samples to the ARG: (1) detecting a set of closest genetic relatives via hashing, (2) running ASMC and (3) ‘threading’ the new sample into the ARG (Fig. 1). Given a new sample, step 1 performs a series of hash table queries to determine the candidate closest samples already in the ARG^24^. We divide up the sites present in the genetic data into nonoverlapping ‘words’ of *S* sites and store hash tables mapping from the possible values of the *i*th word to the samples that carry that word. We use this approach to rapidly detect samples already in the ARG that share words with the target sample and return the top *K* samples with the most consecutive matches. A tolerance parameter *T* controls the number of mismatches allowed in an otherwise consecutive stretch. We also allow the top *K* samples to vary across the genome due to recombination events, by partitioning the genome into regions of genetic distance *L*. Assuming this results in *R* regions, the hashing step outputs a matrix of *R* × *K* sample identities (IDs) containing the predicted top *K* related samples for each region. We note, however, that the hashing step can look arbitrarily far beyond the boundaries of each region to select the *K* samples.

The sample IDs output by step 1 inform step 2, in which ASMC is run over pairs of samples. In each of the *R* regions, ASMC computes the posterior mean and maximum a posteriori TMRCA between the sample being threaded and each of the *K* candidate most related samples. We add burn-in on either side of the region to provide additional context for the ASMC model, 2.0 centimorgans (cM) for all simulation experiments unless otherwise stated and 1.0 cM in real data inference for greater efficiency.

In step 3, ARG-Needle finds the minimum posterior mean TMRCA among the *K* candidates at each site of the genome. Note that both the use of a posterior mean estimator with a pairwise demographic prior and the selection of a minimum among *K* estimated values lead to bias in the final TMRCA estimates (Supplementary Fig. 3h), which we later address using a postprocessing normalization step (see below). The corresponding IDs determine which sample in the ARG to thread to at each site. Because the posterior mean assumes continuous values and changes at each site, we average the posterior mean over neighboring sites where the ID to thread to and the associated maximum a posteriori remain constant. This produces piecewise constant values which determine how high above the sample to thread, with changes corresponding to inferred recombination events. The sample is then efficiently threaded into the existing ARG, utilizing custom data structures and algorithms.

Throughout our analyses we adopted *K* = 64, *T* = 1, *L* = 0.5 cM for array data and *L* = 0.1 cM for sequencing data. We used *S* = 16 in simulations, and in real data analyses we increased *S* as threading proceeded to reduce computation without a major loss in accuracy. For additional details on all three steps in the ARG-Needle algorithm and our parameter choices, see Supplementary Note 1.

### ARG normalization

ARG normalization applies a monotonically increasing mapping from existing node times to transformed node times (similar to quantile normalization), further utilizing the demographic prior provided in input. We compute quantile distributions of node times in the inferred ARG as well as in 1,000 independent trees simulated using the demographic model provided in input under the single-locus coalescent. We match the two quantile distributions and use this to rewrite all nodes in the inferred ARG to new corresponding times (Supplementary Note 1). ARG normalization preserves the time-based ordering of nodes and therefore does not alter the topology of an ARG. It is applied by default to our inferred ARGs and optionally to ARGs inferred by Relate (Extended Data Figs. 2–4 and Supplementary Figs. 1, 3 and 4).

### Simulated genetic data

We used the msprime coalescent simulator^61^ to benchmark ARG inference algorithms. For each run, we first simulated sequence data with given physical length *L* for *N* haploid individuals, with *L* = 1 Mb for sequencing and *L* = 5 Mb for array data experiments. Our primary simulations used a mutation rate of *μ* = 1.65 × 10^−8^ per base pair per generation, a constant recombination rate of *ρ* = 1.2 × 10^−8^ per base pair per generation and a demographic model inferred using SMC++ on CEU (Utah residents with ancestry from Northern and Western Europe) 1,000 Genomes samples^10^. These simulations also output the simulated genealogies, which we refer to as ‘ground-truth ARGs’ or ‘true ARGs’. To obtain realistic SNP data, we subsampled the simulated sequence sites to match the genotype density and allele frequency spectrum of UK Biobank SNP array markers (chromosome 2, with density defined using 50 evenly spaced MAF bins). When running ASMC, we used decoding quantities precomputed for version 1.1, which were obtained using a European demographic model and UK Biobank SNP array allele frequencies, setting two haploid individuals for pairwise TMRCA inference as ‘distinguished’ and sampling 298 haploid individuals as ‘undistinguished’^11^. ASMC and the hashing step of ARG-Needle also require a genetic map, which we computed based on the recombination rate used in simulations.

In addition to our primary simulations, we included various additional simulation conditions where we modified one parameter while keeping all others fixed. First, we varied the recombination rate to *ρ* ∈ {6 × 10^−9^, 2.4 × 10^−8^} per base pair per generation. Second, we used a constant demographic model of 15,000 diploid individuals, for which we generated new decoding quantities to run ASMC. Third, we inferred ARGs using sequencing data, running ASMC in sequencing mode. Fourth, we introduced genotyping errors into the array data. After sampling the array SNPs, we flipped each haploid genotype per SNP and individual with probability *p* (Supplementary Fig. 4).

### Comparisons of ARG inference methods

We compared ASMC-clust and ARG-Needle with the Relate^17^ and tsinfer^15^ algorithms. Relate runs a modified Li-and-Stephens algorithm^62^ for each haplotype, using all other haplotypes as reference panel. It then performs hierarchical clustering on the output to estimate the topology of marginal trees at each site. Finally, it estimates the marginal tree branch lengths using a Markov chain Monte Carlo algorithm with a coalescent prior. tsinfer uses a heuristic approach to find a set of haplotypes that will act as ancestors for other haplotypes and to rank them based on their estimated time of origin. It then runs a variation of the Li-and-Stephens algorithm to connect older ancestral haplotypes to their descendants, forming the topology of the ARG. To improve the performance of tsinfer in the analysis of UK Biobank array data, the authors developed an alternative approach where subsets of the analyzed individuals are added as potential ancestors^15^. This approach was motived by the sparsity of the variant sites, so we refer to it as ‘tsinfer-sparse’, obtaining its code from ref. ^63^.

We ran Relate with the mutation rate, recombination rate and demographic model used in simulations. We kept Relate’s default option which limits the memory used for storing pairwise matrices to 5 GB. Because the branch lengths output by tsinfer and tsinfer-sparse are not calibrated, we omitted these methods in comparisons for metrics involving branch lengths. For each choice of sample size, we generated genetic data using five random seeds (25 random seeds in Extended Data Fig. 3d,e) and applied ARG-Needle, ASMC-clust, Relate, tsinfer and (when dealing with array data) tsinfer-sparse to infer ARGs. Due to scalability differences, we ran ASMC-clust and Relate in up to *N* = 8,000 haploid samples (*N* = 4,000 for sequencing) and ARG-Needle, tsinfer and tsinfer-sparse in up to *N* = 32,000 haploid samples. All analyses used Intel Skylake 2.6 GHz nodes on the Oxford Biomedical Research Computing cluster.

The Robinson–Foulds metric^27^ counts the number of unique mutations that can be generated by one tree but not the other. Because polytomies can skew this metric, we randomly break polytomies as done in ref. ^15^. We report a genome-wide average, rescaled between 0 and 1.

We generalized the Robinson–Foulds metric to better capture the accuracy in predicting unobserved variants by incorporating ARG branch lengths. To this end, we consider the probability distribution of mutations that arise from uniform sampling on an ARG, and compare the resulting distributions for the true and inferred ARG using the total variation distance, a metric for comparing probability measures. Polytomies do not need to be broken using this metric, as they simply concentrate the probability mass on fewer predicted mutations. We refer to this metric as the ARG total variation distance, and note that it bears similarities to previous extensions of the Robinson–Foulds metric^64,65^ (see Supplementary Note 2 for further details, including an extension that stratifies by allele frequencies).

We also used the KC topology-only distance averaged over all positions to compare ARG topologies. We observed that for methods that output binary trees (Relate, ASMC-clust and ARG-Needle), performance substantially improved when we selected inferred branches at random and collapsed them to create polytomies (solid lines in Extended Data Figs. 1c and 3g), suggesting that the KC topology-only distance rewards inferred ARGs with polytomies. We further quantified the amount of polytomies output by tsinfer and tsinfer-sparse as the mean fraction of nonleaf branches collapsed per marginal tree, observing that when polytomies were randomly broken^15^, performance on the KC topology-only distance deteriorated (dashed lines in Fig. 2d and Extended Data Figs. 1b,c and 3f,g). To account for these observations, we compared all methods both with the restriction of no polytomies and with allowing all methods to output polytomies (Fig. 2d and Extended Data Figs. 1b,d and 3f,h). In the latter case, we formed polytomies in ARGs inferred by Relate, ASMC-clust and ARG-Needle using a heuristic to select and collapse branches that are less confidently inferred. For each marginal binary tree, we ordered the *N* − 2 nonleaf branches by computing the branch length divided by the height of the parent node, and collapsed a fraction *f* of branches for which this ratio is smallest (for additional details, see Supplementary Note 2).

We used the pairwise TMRCA RMSE metric to measure accuracy of inferred branch lengths. The KC distance may also consider branch lengths^28^, and we performed evaluations using the branch-length-aware versions of the KC distance with parameter *λ* = 1, which compares lengths between pairwise MRCA events and the root, and *λ* = 0.02, which combines branch length and topology estimation (Supplementary Fig. 1).

Supplementary Note 2 provides further details on the computation of these metrics and their interpretation in the context of ARG inference and downstream analyses.

### ARG-MLMA

We developed an approach to perform MLMA of variants extracted from the ARG, which we refer to as ARG-MLMA. We sampled mutations from a given ARG using a specified rate *μ* and applied a mixed model association test to these variants. Note that each mutation occurs on a single branch of marginal trees, so that recurrent mutations are not modeled.

For simulation experiments (Fig. 3a and Extended Data Fig. 6) we tested all possible mutations on a true or inferred ARG, which is equivalent to adopting a large value of *μ*. We used sequencing variants from chromosomes 2–22 to form a polygenic background and added a single causal sequencing variant on chromosome 1 with effect size *β*. We varied the value of *β* and measured power as the fraction of runs (out of 100), detecting a significant association on the ARG for chromosome 1. For ARG-MLMA UK Biobank analyses we adopted *μ* = 10^−5^, also adding variants sampled with *μ* = 10^−3^ to locus-specific Manhattan plots to gain further insights. For additional details on our ARG-MLMA methods, including the determination of significance thresholds, see Supplementary Note 4.

### Construction of ARG-GRMs

Consider *N* haploid individuals, *M* sites and genotypes *x*_*ik*_ for individual *i* at site *k*, where variant *k* has mean *p*_*k*_. Under an infinitesimal genetic architecture, the parameter *α* captures the strength of negative selection^30,66^, with a trait’s genetic component given by *g*_*i*_ = ∑_*k*_ *β*_*k*_*x*_*ik*_ where Var(*β*_*k*_) = (*p*_*k*_(1 − *p*_*k*_))^*α*^. Using available markers, a common estimator for the *ij*-th entry of the *N* × *N* GRM^21^ is

1 $$K_\alpha \left( {i,j} \right) = \frac{1}{M}\mathop {\sum }\limits_{k = 1}^M \frac{{\left( {x_{ik} - p_k} \right)\left( {x_{jk} - p_k} \right)}}{{\left[ {p_k\left( {1 - p_k} \right)} \right]^{ - \alpha }}}.$$

Given an ARG, we compute the ARG-GRM as the expectation of the marker-based GRM that would be obtained using sequencing data, assuming that mutations are sampled uniformly over the area of the ARG. When sequencing data are unavailable but an ARG can be estimated from an incomplete set of markers, the ARG-GRM may provide a good estimate for the sequence-based GRM. We briefly describe how ARG-GRMs are derived from the ARG for the special case of *α* = 0. We discuss the more general case and provide further derivations in Supplementary Note 3.

Assuming *α* = 0, equation (1) is equivalent (up to invariances described in Supplementary Note 3) to the matrix whose *ij*-th entry contains the number of genomic sites at which sequences *i* and *j* differ (that is, their Hamming distance). This may be expressed as

$$K_{\mathrm{H}}\left( {i,j} \right) = \frac{1}{M}\mathop {\sum }\limits_{k = 1}^M x_{ik} \oplus x_{jk},$$

where ⊕ refers to the exclusive or (XOR) function. Assume there are *L* base pairs in the genome and a constant mutation rate per base pair of *μ*, and let *t*_*ijk*_ denote the TMRCA of *i* and *j* at base pair *k*. The *ij*-th entry of the ARG-GRM is equivalent to the expected number of mutations carried by only one of the two individuals, which is proportional to the sum of the pairwise TMRCAs across the genome (Extended Data Fig. 7a):

$$\begin{array}{l}K_{\mathrm{ARG}}\left( {i,j} \right) = {\Bbb E}\left[ {K_{\mathrm{H}}\left( {i,j} \right)|\mathrm{ARG}} \right]\\ = \mathop {\sum }\limits_{k = 1}^L P\left( {\mathrm{Poisson}\left( {2\mu t_{ijk}} \right) > 0} \right) = \mathop {\sum }\limits_{k = 1}^L 1 - {{{\mathrm{exp}}}}\left( { - 2\mu t_{ijk}} \right) \approx \mathop {\sum }\limits_{k = 1}^L 2\mu t_{ijk}.\end{array}$$

For increased efficiency, we compute a Monte Carlo ARG-GRM by uniformly sampling new mutations on the ARG with a high mutation rate and apply equation (1) to build the ARG-GRM using these mutations. We used simulations to verify that Monte Carlo ARG-GRMs converge to exactly computed ARG-GRMs for large mutation rates, saturating at *μ* ≈ 1.65 × 10^−7^ (Extended Data Fig. 7b,c), the default value we used for ARG-GRM computations. Stratified Monte Carlo ARG-GRMs may also be computed by partitioning the sampled mutations based on allele frequency, LD or allele age^36,31,67,68^ (Supplementary Note 3).

### ARG-GRM simulation experiments

We simulated polygenic traits from haploid sequencing samples for various values of *h*^2^ and *α*. We varied the number of haploid samples *N* but fixed the ratio *L*/*N* throughout experiments, where *L* is the genetic length of the simulated region. For heritability and polygenic prediction experiments, we adopted *L*/*N* = 5 × 10^−3^ Mb per individual. For association experiments, we simulated a polygenic phenotype from 22 chromosomes, with each chromosome consisting of equal length *L*/22 and *L*/*N* = 5.5 × 10^−3^ Mb per individual. Mixed-model prediction *r*^2^ and association power may be roughly estimated as a function of *h*^2^ and the ratio *N*/*M*, where *M* is the number of markers^39,69,70^. We thus selected values of *M* and *L* such that the *N*/*M* ratio is kept close to that of the UK Biobank (*L* = 3 × 10^3^ Mb, *N* ≈ 6 × 10^5^).

We computed GRMs using ARGs, SNP data, imputed data (IMPUTE4 (ref. ^38^) within-cohort imputation) and sequencing data, and performed complex trait analyses using GCTA^21^. Polygenic prediction used cvBLUP^71^ leave-one-out prediction within GCTA. ARG-GRM association experiments (Fig. 3c and Extended Data Fig. 8c,f) tested array SNPs on each chromosome while using GRMs built on the other 21 chromosomes to increase power, measured as the relative increase of mean −log_10_(*P*) compared with linear regression. We observed that MAF-stratification for ARG-GRMs of true ARGs enabled robust heritability estimation and polygenic prediction if *α* is unknown (Extended Data Fig. 8g). In experiments involving inferred ARGs (Fig. 3b and Supplementary Fig. 8), we applied MAF-stratification for ARG-Needle ARGs and imputed data, but not for SNP data, for which GCTA did not converge.

### ARG-Needle inference in the UK Biobank

Starting from 488,337 samples and 784,256 available autosomal array variants (including SNPs and short indels), we removed 135 samples (129 withdrawn, 6 due to missingness > 10%) and 57,126 variants (missingness > 10%). We performed reference-free phasing of the remaining variants and samples using Beagle 5.1 (ref. ^72^) and extracted the unrelated white British ancestry subset defined in ref. ^38^, yielding 337,464 samples. We built the ARG of these samples using ARG-Needle, using parameters described above. We parallelized the ARG inference by splitting phased genotypes into 749 nonoverlapping ‘chunks’ of approximately equal numbers of variants. We added 50 variants on either side of each chunk to provide additional context for inference and independently applied ARG normalization on each chunk. For our brief comparison of ARG inference methods in real data (Supplementary Fig. 6c,d), we repeatedly sampled independent subsets of *N* = 2,000 and *N* = 16,000 diploid individuals, and inferred the ARG for these individuals using the first chunk in the second half of chromosome 1, which amounted to 7.5 Mb.

### Genealogy-wide association scan in the UK Biobank

To process phenotypes (standing height, alkaline phosphatase, aspartate aminotransferase, low-density lipoprotein (LDL)/high-density lipoprotein (HDL) cholesterol, mean platelet volume and total bilirubin) we first stratified by sex and performed quantile normalization. We then regressed out age, age squared, genotyping array, assessment center and the first 20 genetic principal components computed in ref. ^38^. We built a noninfinitesimal BOLT-LMM mixed model using SNP array variants, then tested HRC + UK10K-imputed data^38,40,41^ and variants inferred using the ARG (ARG-MLMA, described above). For association of imputed data (including SNP array) we restricted to variants with Hardy–Weinberg equilibrium *P* > 10^−12^, missingness < 0.05 and info score > 0.3 (matching the filtering criteria adopted in ref. ^38^). For all analyses we did not test variants with an MAC < 5 and used MAF thresholds detailed below.

### Association analysis of seven traits

Using the filtering criteria above and no additional MAF cutoff, we obtained resampling-based genome-wide significance thresholds of *P* < 4.8 × 10^−11^ (95% CI: 4.06 × 10^−11^, 5.99 × 10^−11^) for ARG and *P* < 1.06 × 10^−9^ (95% CI: 5.13 × 10^−10^, 2.08 × 10^−9^) for imputation (Supplementary Table 1 and Supplementary Note 4). After performing genome-wide MLMA for the seven traits, we selected genomic regions harboring low-frequency (0.1% ≤ MAF < 1%), rare (0.01% ≤ MAF < 0.1%) or ultra-rare (MAF < 0.01%) variant associations. We then formed regions by grouping any associated variants within 1 Mb of each other and adding 0.5 Mb on either side of these groups.

We next performed several further filtering and association analyses using a procedure similar to ref. ^43^ to extract sets of approximately independent signals (‘independent’ for short; Supplementary Tables 2–5 and Supplementary Note 4). Of the seven phenotypes, total bilirubin did not yield any rare or ultra-rare independent signals and height did not yield any independent ultra-rare signals. We leveraged the UK Biobank WES data to validate and localize independent associations. We extracted 138,039 exome-sequenced samples that overlap with the analyzed set of white British ancestry individuals and performed lift-over of exome sequencing positions from genome build hg38 to hg19. We computed pairwise LD between the set of independent associated variants and the set of all WES variants, defining the ‘WES partner’ of an independent variant to be the WES variant with largest *r*^2^ to it. In a few instances, the same WES partner was selected by two ARG variants or two imputation variants (Supplementary Tables 2–5). Additionally, three WES partners were selected by one ultra-rare ARG and one rare imputation variant, and one WES partner was selected by one rare ARG and two ultra-rare imputation variants; these WES partners counted towards the 36 WES partners identified by both methods in rare and ultra-rare analyses, but were not counted as jointly identified when restricting to only rare or ultra-rare categories (as in Fig. 4a). We labeled WES variants by gene and functional status (‘loss-of-function’ and ‘other protein altering’; Supplementary Note 4) based on annotations obtained using the Ensembl Variant Effect Predictor (VEP) tool^73^.

### Association analysis for higher frequency variants and height

For genome-wide association analyses of higher frequency variants and height, we matched filtering criteria used in ref. ^38^, retaining imputed variants that satisfy the basic filters listed above, as well as MAF ≥ 0.1%. Using these criteria, we estimated a resampling-based genome-wide significance threshold of 4.5 × 10^−9^ (95% CI: (2.2 × 10^−9^, 9.6 × 10^−9^); Supplementary Table 1). To facilitate direct comparison, we aimed to select parameters that would result in a comparable significance threshold for the ARG-MLMA analysis. Two sets of parameters satisfied this requirement: 3.4 × 10^−9^ (95% CI: 2.4 × 10^−9^, 5 × 10^−9^), obtained for *μ* = 10^−5^, MAF ≥ 1%; and 4 × 10^−9^ (95% CI: 3.1 × 10^−9^, 5.3 × 10^−9^), obtained for *μ* = 10^−6^, MAF ≥ 0.1%. We selected the former set of parameters, as a low sampling rate of *μ* = 10^−6^ leads to worse signal-to-noise and lower association power. We thus used a significance threshold of *P* < 3 × 10^−9^ for all analyses of higher frequency variants. We used PLINK^74,75^ (v.1.90b6.21 and v.2.00a3LM) and GCTA^21^ (v.1.93.2) to detect approximately independent associations using COJO^47^, retaining results with COJO *P* < 3 × 10^−9^ (Fig. 5e,f, Extended Data Fig. 10e, Supplementary Fig. 11 and Supplementary Note 4).

### Statistics and reproducibility

For real data analysis in the UK Biobank, we included all 337,464 individuals of white British ancestry (as reported in ref. ^38^) who did not have genotype missingness > 10% and had not withdrawn from the UK Biobank at the time of our analysis. To further explore our findings, we selected the 138,039 of these individuals who were exome sequenced in the 200,000 UK Biobank whole-exome sequencing release.

### Ethics

UK Biobank data were analyzed after approval of UK Biobank proposal no. 43206.

### Reporting summary

Further information on research design is available in the Nature Portfolio Reporting Summary linked to this article.

## Online content

Any methods, additional references, Nature Portfolio reporting summaries, source data, extended data, supplementary information, acknowledgements, peer review information; details of author contributions and competing interests; and statements of data and code availability are available at 10.1038/s41588-023-01379-x.

## Supplementary information

Supplementary Information Supplementary Notes 1–4 and Figs. 1–12.

Reporting Summary

Supplementary Tables 1–5 Table 1. Resampling-based tests of subsets of HRC-imputed and ARG-Needle-inferred ARG variants against random phenotypes. Table 2. Approximately independent ARG signals with MAF < 0.1% (*N* = 134) from association of quantitative traits in 337 K UK Biobank individuals, along with the best partner according to LD in whole-exome sequencing (WES) data of 138 K UK Biobank individuals. Table 3. Approximately independent imputation signals with MAF < 0.1% (*N* = 64) from association of quantitative traits in 337 K UK Biobank individuals, along with the best partner according to LD in whole-exome sequencing (WES) data of 138 K UK Biobank individuals. Table 4. Approximately independent ARG signals with 0.1% ≤ MAF < 1% (*N* = 103) from association of quantitative traits in 337 K UK Biobank individuals, along with the best partner according to LD in whole-exome sequencing (WES) data of 138 K UK Biobank individuals. Table 5. Approximately independent imputation signals with 0.1% ≤ MAF < 1% (*N* = 100) from association of quantitative traits in 337 K UK Biobank individuals, along with the best partner according to LD in whole-exome sequencing (WES) data of 138 K UK Biobank individuals.

## Data Availability

COJO association signals for higher frequency ARG variants with height are available at 10.5281/zenodo.7411562. VEP annotations were generated using the Ensembl VEP tool (v.101.0, output produced February 2021), https://www.ensembl.org/info/docs/tools/vep/index.html. UK Biobank data can be accessed by approved researchers through https://www.ukbiobank.ac.uk/. Other datasets were downloaded from the following URLs: summary statistics from whole-exome imputation from 50,000 sequences^43^, https://data.broadinstitute.org/lohlab/UKB_exomeWAS/; likely causal associations from whole-exome imputation from 50,000 sequences^43^, https://www.nature.com/articles/s41588-021-00892-1 Supplementary Table 3; GIANT consortium summary statistics in ~700,000 (ref. ^46^), https://portals.broadinstitute.org/collaboration/giant/index.php/GIANT_consortium_data_files.

## Code availability

The arg-needle and arg-needle-lib software packages, which implement the ARG-Needle and ASMC-clust methods as well as methods for the main analyses in this paper, are available at https://palamaralab.github.io/software/argneedle/. Python packages can be downloaded at https://pypi.org/project/arg-needle/ and https://pypi.org/project/arg-needle-lib/; analysis scripts are available at 10.5281/zenodo.7745745. External softwares used in the current study were obtained from the following URLs: msprime (v.0.7.4), https://pypi.org/project/msprime/; tsinfer (v.0.1.4), https://pypi.org/project/tsinfer/; tsinfer scripts for sparse data (accessed January 2022), https://github.com/mcveanlab/treeseq-inference; Relate (v.1.0.15), https://myersgroup.github.io/relate/; ARGON (v.0.1.160415), https://github.com/pierpal/ARGON/; DASH (v.1.1) and GERMLINE (v.1.5.3), http://www1.cs.columbia.edu/~gusev/dash/; IMPUTE4 (v.4.1.2), https://jmarchini.org/software/#impute-4; Beagle (v.5.1), https://faculty.washington.edu/browning/beagle/b5_1.html; PLINK (v.1.90b6.21), https://www.cog-genomics.org/plink/; PLINK (v.2.00a3LM), https://www.cog-genomics.org/plink/2.0/; GCTA (v.1.93.2), https://cnsgenomics.com/software/gcta/; BOLT-LMM (v.2.3.2), https://alkesgroup.broadinstitute.org/BOLT-LMM/downloads/; LiftOver (used April 2021), https://genome.ucsc.edu/cgi-bin/hgLiftOver.
