# Supplementary material for: Biobank-scale inference of ancestral recombination graphs enables genealogical analysis of complex traits
Source: Nat Genet. 2023 May 1;55(5):768–76. doi: 10.1038/s41588-023-01379-x (PMC10181934; doi:10.1038/s41588-023-01379-x)
Supplement: Supplementary file 1 — Supplementary Notes 1–4 and Figs. 1–12. [file 41588_2023_1379_MOESM1_ESM.pdf]

nature genetics

Article

https://doi.org/10.1038/s41588-023-01379-x

Biobank-scale inference of ancestral recombination graphs enables genealogical analysis of complex traits

In the format provided by the authors and unedited

Supplementary Information Contents

Supplementary Note 1 **3**

ARG definition and representation . . . 3

Memory-efficient ASMC-clust extension . . . 5

ARG-Needle algorithm description . . . 5

ARG-Needle step 1: shortlisting of closest relatives via genotype hashing . . . 6

ARG-Needle step 2: ASMC queries . . . 8

ARG-Needle Step 3: processing the ASMC output and performing threading . . . 9

ARG normalization . . . 10

Theoretical properties . . . 11

Recovery of marginal trees using true coalescence times . . . 11

Relationship between ASMC-clust and ARG-Needle under ultrametric TMRCAs . . . 12

Supplementary Note 2 **14**

Evaluating metrics via stabbing queries . . . 14

ARG total variation distance as a generalization of the Robinson-Foulds distance . . . 14

Pairwise TMRCA RMSE and TMRCA scatter plots . . . 17

KC distance . . . 18

Supplementary Note 3 **22**

Additional experimental details for ARG-GRMs . . . 22

Notation for sequence-based GRMs . . . 23

From sequence-based GRMs to ARG-GRMs . . . 23

Three GRM invariances . . . 25

Exact ARG-GRM, haploid and general α . . . 28

Exact ARG-GRM, diploid and general α . . . 30

Supplementary Note 4 **32**

ARG-MLMA methods . . . 32

Comparison to IBD-based association . . . 33

Computation of resampling-based significance thresholds 33
Association analysis of 7 traits 34
Association analysis for higher frequency variants with height 35
ARG-based genotype imputation 35

References (Supplementary Information) **37**

Supplementary Figures **41**

Supplementary Note 1: Additional details on ARG inference

In this note we clarify our definition of an ancestral recombination graph (ARG [1]), provide detail for the three steps of the ARG-Needle algorithm (see Fig. 1), describe the ARG normalization step, and discuss a memory-efficient extension of the ASMC-clust algorithm.

ARG definition and representation

We briefly clarify the definition and representation of ARG used in this work.

Representing recombination events and individuals

In this work, we refer to an ancestral recombination graph (ARG) as a graph representing ancestral relationships along the genome for a set of samples. The nodes of this graph represent haplotypes, which are assigned properties such as time at which the individual carrying the haplotype lived and start/end positions. Individuals may be represented as a collection of contemporary and non-overlapping haplotypes. Edges in the graph represent ancestor/descendant relationships across haplotypes and individuals, which can be modified by the occurrence of recombination events.

Stochastic process such as the coalescent with recombination [2, 3] (hereafter "the coalescent") produce an ARG. It is convenient to describe the coalescent by means of the simulation algorithm that samples a specific ARG instance. In its simplest form (ignoring e.g., natural selection, migration, gene conversion, and mutation events), backwards-in-time coalescent simulation proceeds by sampling coalescence and recombination events. As simulation proceeds, a graph (the ARG) is built: new nodes are created and connected by new edges as a result of coalescence or recombination. Coalescence events result in nodes with multiple child edges, while recombination events result in nodes with multiple parent edges.

Although an ARG may be used to represent extensive details of a sample's history, ARG-Needle focuses on inferring a compact subset of the ARG that is relevant for downstream analyses (also see e.g., Hudson's ARG or the "small ARG" [3, 4, 5]). In addition, ARG-Needle infers the location of recombination events using a heuristic as part of the threading procedure (see the description of Step 3 below) but does not attempt to precisely infer the time of a recombination event. Instead, recombination events are stored using the closest coalescence or leaf node below the time at which a recombination is inferred to have happened. This removes the need to include ARG nodes that are solely used to represent the timing of recombination events, which do not directly affect downstream analyses such as the sampling of mutations and the generation of sequencing data. Improved estimation of the timing of recombination events may be obtained by performing a post-processing of the ARG, which we do not explore in this work. ARGs inferred by ARG-Needle also do not distinguish between an ancestral haplotype and an ancestor, so that the ancestors in inferred ARGs never include "trapped material" [6].

Tree-based vs graph-based ARG representation

One key property of the ARG is that although it is a potentially cyclic graph (ignoring directionality of the edges; haplotypes may recombine and subsequently coalesce again), it simplifies to a tree if we only consider the set of nodes and edges spanning a specific genomic position. For this reason, an ARG induces a sequence of trees along the genome. This property is leveraged in formulations of the coalescent as a process that generates a series of correlated marginal trees along the genome, and in simulators of this process [7, 8, 9, 10, 11, 12]. However, despite the close connections between tree-based and graph-based encodings, the graph-based representation that more naturally arises in the backwards-in-time view of the process is more memory-efficient than a representation based on a collection of marginal trees, which requires storing of the same ancestral haplotype (ARG node) multiple times in neighboring trees.

Due to these different interpretations and properties, other approaches for genealogical inference sometimes refer to sequences of trees and ARGs with subtle distinctions. [13] and [14] (within the `tskit` library) use a graph-based ARG representation (a set of nodes and edges), but refer to it as a tree sequence, highlighting the sequential interpretation of an ARG. The output of Relate [15] is referred to as a sequence of trees approximating the ARG. Although these are closely related, this places an emphasis on the fact that the Relate algorithm does not strongly enforce correlation across neighboring marginal trees (making the graph-based representation converge towards the tree-based representation). In our work we use a graph-based representation of the ARG, which is an adaptation of the representation used within the ARGON simulator [16]. Both the ARGON graph-based representation, used in this work, and the tree sequence graph-based representation, used in the tsinfer software and the `tskit` framework, lead to substantial computational and storage gains compared to representing individual marginal trees.

Although, due to intrinsic properties of ARGs, the graph inferred by ARG-Needle may also be interpreted as representing a sequence of trees, we solely refer to it as an ARG. Because the ARG-Needle algorithm enforces some degree of node sharing across neighboring marginal trees, the graph-based representation of ARG-Needle inferred ARGs is more parsimonious than a representation based on independent marginal trees. On the other hand, the ASMC-clust algorithm does not encourage substantial correlation across marginal trees, although it may in principle be extended to encourage information sharing across neighboring sites.

ARG-Needle ARG format

ARG-Needle is coded in C++ and Python. In an ARG-Needle ARG, nodes represent ancestral haplotypes, storing metadata such as their age (in generations). Edges connect pairs of nodes implying ancestor/descendant relationships, and store start and end coordinates for the inherited chromosomal region. The set of parents and children of a node may be different for different coordinates along its genomic extent. Access to a node's ancestor or children at a particular position thus requires $O(\log K)$

computation, where K is the total number of ancestors or children proceeding from the node. In the ARG representation used in the ARGON simulator [16], on the other hand, the set of children (and the set of descendant leaves) of each ancestral node remains the same along its genomic extent, while parents may vary. This leads to $O(1)$ computational cost to retrieve a node's children and $O(\log K)$ cost to retrieve parents at a genomic position. Both representations are efficient in terms of memory and computational cost of common ARG queries, with tradeoffs between the cost of some operations, such as retrieving the set of descendants of a node, and the number of nodes/edges needed to represent the ARG. ARG-Needle implements various algorithms for complex trait analysis. It can export inferred ARGs using a serialized graph format and can convert to and from the `tskit` file format.

Memory-efficient ASMC-clust extension

Assuming N samples and M sites, the default ASMC-clust algorithm requires $O(N^2M)$ memory and runtime complexity. We implemented an option for ASMC-clust to instead use $O(N^2 + NM)$ memory, at the cost of more runtime. In the default algorithm, we run ASMC on all $\binom{N}{2}$ pairs of samples across M sites, and store the results in an $\binom{N}{2}$ by M matrix. We then iterate through the TMRCA values at each of the M sites and perform UPGMA hierarchical clustering. The resulting ARG consists of M trees, each over N nodes, which can be represented in $O(NM)$ memory. Therefore, the memory bottleneck comes from the storage of the TMRCA results, which takes up $O(N^2M)$ memory.

To reduce the memory required from storing the TMRCA results, we iterate through groups of M_{max} sites, each time computing and storing the TMRCA results for those sites, performing UPGMA clustering, and saving the clustered trees. This results in $O(N^2M_{max} + NM)$ memory usage. As described in Methods, however, we pad additional sites (2 cM in simulations and 1 cM in real data) on either end of the region to provide the ASMC HMM with context. Using this approach, each site will therefore be processed multiple times by ASMC, leading to a time complexity somewhere between $O(N^2M)$ and $O(N^2M^2)$, depending on the choice of M_{max} and the number of SNPs provided as context in each ASMC run.

In our simulations we fixed $M_{max} = 300$ for SNP data and $M_{max} = 2000$ for sequencing data. This led to memory usage that scaled quadratically as a function of N. Relate faces a similar issue of potentially quadratic memory, which is also solved by only storing distance matrices for a certain number of sites at a time. However, instead of fixing the number of sites, Relate enforces a maximum memory for the storage of distance matrices and uses that to determine the appropriate number of sites. We ran Relate using its default setting, which sets the maximum amount of memory for these calculations to 5 GB.

ARG-Needle algorithm description

ARG-Needle starts with an empty ARG, and iteratively adds one new haploid sample at a time to the ARG. Adding the first sample generates a single ARG node at the present time spanning the whole

genomic region. For each additional sample, three steps are used to generate a threading instruction and add the new sample to the ARG: (1) perform hash table queries; (2) run ASMC [17] to form the threading instruction; (3) thread the new sample to the ARG.

In Methods, we introduced these three steps and their corresponding parameters. Of the three steps, the second step (ASMC) dominates runtime for small N and the first step (hashing) dominates runtime for large N. The parameters K, L, and S (see Methods) each carry an accuracy versus runtime tradeoff, with large K, small L, and small S leading to a more accurate but slower algorithm. We decided on default parameters of $K = 64$ and $L = 0.5$ cM for array data and $K = 64$ and $L = 0.1$ cM for sequencing data. In experiments with simulated genotypes, we fixed the hash word size at $S = 16$. We set $T = 1$ in simulations and real data analyses to enable robustness to a single genotyping error or recent mutation event in otherwise closely related samples. In real data analysis we set $K = 64$ and $L = 0.5$ cM. We also implemented a larger primary hash word size S_1, leveraged for increased speed, and a smaller "backup hash word size" S_2, used for samples where more fine-grained hashing was needed (see below). We set $S_1 = 16$ and $S_2 = 8$ when threading the first 50K individuals and set $S_1 = 64$ and $S_2 = 16$ for the remaining 287K individuals. This is because, as threading proceeds, increasingly close relationships and thus increasingly long shared haplotypes are detected between the sample and other individuals in the ARG. We can therefore increase S as threading proceeds, which reduces computational cost without a significant loss in accuracy.

We now describe each of these three steps in detail.

ARG-Needle step 1: shortlisting of closest relatives via genotype hashing

Let the haploid data consist of N samples and M sites. Let the genetic distances of the sites be $g_1, \ldots, g_M$, and let the genotypes be $x_{ij} \in \{0, 1\}$, for $1 \leq i \leq N$ and $1 \leq j \leq M$. The parameters of the hash table operations are a hash word size S, a hash region size L, a hash tolerance T, and a hashing output size K. The units of the hash region size L are in genetic distance, while all other parameters are integers. S and L are used to partition the sites into words and regions. S simply denotes the number of sites in each word. For $1 \leq k \leq \lceil M/S \rceil$, word k consists of sites $a(k) = (k-1) \times S + 1$ to $b(k) = \max(k \times S, M)$ inclusive, resulting in a total of $W = \lceil M/S \rceil$ words. For $1 \leq i \leq N$ and $1 \leq j \leq W$, let w_{ik} be the integer resulting from interpreting the W bits of the kth word for sample i as a binary number, i.e., $w_{ik} = (x_{ib(k)} \ldots x_{ia(k)})_2$.

The words are further grouped into non-overlapping regions such that each region spans a genetic distance of at least L, with the exception of when $L > g_M - g_1$, in which case all words are placed in the same region. Starting with word 1, we find the first index k such that $g_{b(k)} - g_1 \geq L$. This makes up the first region, spanning words 1 to k inclusive. For the second region, we start at word $k+1$ and find the first index k' such that $g_{b(k')} - g_{a(k)} \geq L$. The second region then spans words $k+1$ to k'. We continue in a similar fashion. When we reach the end, if we perfectly have just formed a last region covering all words, the algorithm terminates. Otherwise, we combine any remaining words on to the previous region so that all regions are of genetic distance approximately or just over L. Let the number

of regions resulting from this procedure be R, and for $1 \leq r \leq R$, let $c(r)$ and $d(r)$ be the start and end words, inclusive, $1 \leq c(r) \leq d(r) \leq W$.

The hashing query takes a new sample in input and outputs the top K candidate closest relatives to this sample out of those already in the ARG, for each of the R regions. The purpose of having the hash region size L be in genetic distance is to control for the expected number of recombination events within each region. Recombination will alter the set of closest relatives, but by keeping L sufficiently low it is possible to obtain closest relatives that remain consistent through the region. For each region, the hashing query selects the top K candidate closest relatives by a score which we call the T-tolerant IBS score (for identical-by-state). Given two samples i and i' and a hash tolerance T, we say that i and i' are T-tolerant IBS over $[f, g]$, $1 \leq f \leq g \leq W$, if

$$|\{f \leq k \leq g | w_{ik} \neq w_{i'k}\}| \leq T.$$

The T-tolerant IBS score of samples i and i' over a region r is the number of words in the longest T-tolerant IBS stretch with at least one word of overlap with region r. In notation, we consider the set

$$\{[f, g] | 1 \leq f \leq g \leq W, f \leq d(r), g \geq c(r), |\{f \leq k \leq g | w_{ik} \neq w_{i'k}\}| \leq T\}$$

and take the maximum value of $g - f + 1$ over this set. For each region r, we then take the top K samples with the largest T-tolerant IBS score over this region. In the case of ties, we select the sample(s) that have been added to the ARG more recently, though it is also possible to break ties randomly. If the number of samples with a nonzero score is less than K, only this many results are returned, or in other words, samples scoring zero are not returned. In summary, the hashing query returns a list of at most K top-scoring sample IDs for each of the R regions. Importantly, the count of hash matches to pick these K samples may include words lying arbitrarily far outside the region in question, as long as at least one matching word overlaps the region r.

Our hashing data structure consists of a vector of W hash tables, one for each of the W words. The kth hash table maps from possible values for the kth word, guaranteed to be a number between 0 and $2^S - 1$ inclusive, to a vector of sample IDs which contain that value for the kth word. Every time a sample i' is added to the ARG, we perform $O(W)$ operations to update this data structure. For each k, $1 \leq k \leq W$, we access the entry in the kth hash table at $w_{i'k}$, initializing an empty vector if no entry exists, and appending i' to this vector. Similarly, when we want to query a new sample i', we can index $w_{i'k}$ into the kth hash table to find the samples which match sample i' at the kth word. In this way, we only need to visit the values (i, k) for which there is a match, $w_{ik} = w_{i'k}$.

Suppose there are N' samples already in the ARG, and we want to query the closest relatives for a new sample i'. Our hashing-based implementation runs in $O\left(\overline{N}_{overlap} W\right)$, where $0 \leq \overline{N}_{overlap} \leq N'$ is the average number of samples already in the ARG which match sample i' per word, i.e., the average size of $\{0 \leq i \leq N' | w_{ik} = w_{i'k}\}$ as k varies from 1 to W. As one increases the word size S, $W = \lceil M/S \rceil$ decreases and $\overline{N}_{overlap}$ also decreases because longer words are less likely to be shared. The pattern of this latter trend as a function of S is data set-specific due to factors such as the demographic history of

the population, but in summary, S can be used as a parameter to increase hashing speed at the cost of coarser hashing-based IBS detection.

In building ARGs on 337K UK Biobank samples, the hashing step dominates runtime. We implemented a form of dynamic hashing that uses a primary hash word size S_1 and falls back to a backup hash word size S_2 in some cases, with $S_1 > S_2$. The criterion for falling back to the backup hash word size is parameterized by a factor F. For each region, we compute the sum of the top K T-tolerant IBS scores in that region, and check that this is greater than F times the number of words in the region (in notation, $F \times (d(r) - c(r) + 1)$). If the check fails for any of the regions, then we fall back to the backup hash word size, repeating the entire hash query with S_2 instead of S_1. This has the effect of performing the initial hashing queries with S_2, then transitioning to more and more queries done with S_1 as the ARG-Needle algorithm proceeds. For our run on 337K diploid individuals, we started with $S_1 = 16, S_2 = 8, F = 4$ for adding the first 50K individuals, then progressed to $S_1 = 64, S_2 = 16, F = 8$ for the remaining individuals. Other parameters were set to $T = 1$, $K = 64$, and $L = 0.5$ cM.

ARG-Needle step 2: ASMC queries

In step 2 of the ARG-Needle algorithm, the candidate closest relatives output by hashing are validated using ASMC to yield a more certain estimate of true closest relatives and their TMRCA to the target sample. Each pairwise ASMC comparison takes $O(MD)$ time to run, where D is the number of discretized time bins used to represent the posterior distribution of pairwise coalescence time (we use $D = 69$, the default for ASMC v1.0). With no hashing, each new sample being threaded would need to be compared against all existing samples in the ARG using ASMC, yielding $O(N^2MD)$ runtime overall. By only selecting K candidate closest relatives for each sample being threaded, the total runtime is instead linear in N. Note that detection of the closest relative along the genome may also be achieved, for instance, using the Li-and-Stephens algorithm [18], for an overall cost quadratic in N. Step 1 of ARG-Needle thus functions as a heuristic pre-selection strategy, similar to the use of genotype hashing or the positional Burrows-Wheeler transform [19] to speed up genotype imputation [20, 21, 22], phasing [23], and identity-by-descent (IBD) detection [24].

For each of the R regions (partitioned based on genetic distance, see above), step 1 of ARG-Needle returns up to K IDs representing the candidate closest relatives to this sample within the region. Using our earlier definitions, region r contains words with indices from $c(r)$ to $d(r)$ inclusive, with $1 \leq c(r) \leq d(r) \leq W$, which means it contains SNPs with indices from $a(c(r))$ to $b(d(r))$ inclusive, $1 \leq a(c(r)) \leq b(d(r)) \leq M$. We use ASMC (run in array or sequencing mode, depending on the experiment) to predict the pairwise coalescence time posterior distribution between the new sample and each of the K samples at each of these SNP positions, resulting in an output of size $K \times (b(d(r)) - a(c(r)) + 1) \times D$. Because the ASMC Hidden Markov Model (HMM) takes into account sequential context when making a prediction, we pad the input region by adding additional sites for burn-in on either side, as specified in each analysis (see Methods). This increases accuracy at the

cost of slightly increased runtime. The ASMC output for each pair and site is a posterior distribution, given as probabilities over the D discretized time bins. We use it to extract the posterior mean and the mode, or maximum a posteriori (MAP) values. This does not affect the runtime complexity, as for each site and pair computing these summaries takes $O(D)$ time. Before proceeding to step 3, we aggregate these summaries so that at each site we have at most K triplets, which represent the ID of the candidate closest relative, the posterior mean of the TMRCA with the target sample, and the MAP.

ARG-Needle Step 3: processing the ASMC output and performing threading

In the final step of the ARG-Needle algorithm, we use the ASMC output to add the new sample to the ARG. This takes place using a "threading" operation, named after the terminology coined by ARGweaver [25]. We first describe the ARG-Needle threading operation, then explain how we perform threading using the ASMC output.

We define a "threading instruction" to contain the sample ID of a closest relative and their TMRCA to the target sample, at each position along the genome. If we assume a genomic extent $[s, t)$ and sample IDs $\{1, \ldots, N'\}$ already in the ARG, a threading instruction may be represented as a function $f : [s, t) \to \{1, \ldots, N'\} \times (0, \infty)$, assigning to each position x a pair $f(x) = (i(x), T(x))$, where $i(x)$ is a sample ID and $T(x) > 0$ is a time.

We now describe the threading operation in terms of how it affects each marginal tree. (Our actual implementation considers threading as an ARG-wide problem and is more efficient; we take this perspective for simplicity.) Consider a position x with a marginal tree over N' samples, and suppose the threading instruction has $f(x) = (i, T)$. First, a new node u for sample $N' + 1$ is created. Next, let v be the oldest ancestor node of sample i with a time less than or equal to T. One of three conditions holds:

1. If v has time less than T and v has a parent node v' (necessarily with time greater than T), create a new node w with time T. Delete the edge between v and v' and add three new edges between pairs (u, w), (v, w), and (w, v').
2. If v has time less than T but does not have a parent node, then v is the root node of the marginal tree at x. Create a new node w with time T and add two new edges between pairs (u, w) and (v, w). (Node w becomes the new root node.)
3. If v has time T, then create an edge going from u to v.

These three operations describe how to perform threading on marginal trees. Note that the third operation creates polytomies: nodes with more than two children at a position. We assume this operation exists in our theoretical results (see below), but in practice, it is extremely unlikely as the time must perfectly match. Our inferred ARGs always choose a different time and never contain polytomies.

Given the output of step 2, we generate a threading instruction as follows. At each site, we select the sample $i(x)$ to be the one with smallest ASMC posterior mean TMRCA among the K candidate

closest relatives (ties are arbitrarily broken). To compute the time $T(x)$, we select regions where both $i(x)$ and the TMRCA MAP with $i(x)$ remain constant and compute the average posterior mean TMRCA to $i(x)$ within each such region. This has several benefits over using the raw value of either the MAP or the posterior mean at each site. Although changes in the MAP better reflect IBD segment breakpoints, the MAP output by ASMC only takes one of D possible values, which would lead to a large number of polytomies in the ARG. The MAP also has a higher RMSE for the TMRCA compared to the posterior mean. The posterior mean, on the other hand, takes different values at each site, which would reduce haplotype sharing across marginal trees and produce a less compact ARG (as in the case of ASMC-clust). By combining the MAP and posterior mean in the threading instruction we thus obtain better estimates for the location of recombination breakpoints without sacrificing accuracy or compactness of the inferred ARG. Note that while the output of ASMC includes a measure of uncertainty by producing a posterior TMRCA distribution at each site, our threading instructions only contain point estimates, which are built by considering different aspects of the posterior output by ASMC. We also note that the operation of taking the minimum of several ASMC posterior means may lead to a bias in addition to any biases already present in the output of ASMC due to, for instance, the use of time discretization and demographic prior information for a pairwise coalescence model (see Supplementary Fig. 3). Our ARG normalization procedure, described next, aims to mitigate these biases.

ARG normalization

Although the posterior mean has a lower RMSE than the MAP in estimating TMRCAs, it tends to be more biased towards the average coalescent time induced by the demographic prior, particularly in sparser array data. For this reason, we observed that ARGs inferred using the above definition of threading instruction tend to overestimate the time of recent coalescence events and underestimate the height of root nodes. We thus developed a procedure, which we call ARG normalization, to further leverage the demographic prior to reduce the bias due to the use of posterior mean estimates, while preserving the inferred ordering of coalescent events (see Supplementary Fig. 3). ARG normalization performs a quantile normalization of the heights of inferred ARG nodes, rescaling them to match the quantiles observed in 1,000 independent trees sampled from the demographic prior (after accounting for the span of inferred ARG nodes).

In more detail, assuming the 1,000 simulations generate Q non-leaf nodes with times that can be ordered increasingly from t_1 to t_Q, we compute quantiles from the simulated trees by assigning quantile $(2i-1)/(2Q)$ to time t_i. We also assign quantile 0 to time 0 and quantile 1 to time $1.05 \times t_Q$ to ensure that the mapping is strictly increasing. Given an inferred ARG, we sought to compute an analogous quantile distribution of node times that was sensitive to differences between nodes spanning short vs. long segments of ancestral material and sensitive to polytomies. For each edge in the inferred ARG, we recorded the time of its parent node and the distance in base pairs it spanned. We aggregated these time-distance pairs to obtain Q' distinct parent node times, ordered increasingly from t_1 to $t_{Q'}$, and

corresponding aggregate distances spanned by edges with that parent node time, d_1 to $d_{Q'}$. We assigned quantile value $(d_1 + d_2 + \ldots + d_{i-1} + d_i/2)/(d_1 + \ldots + d_{Q'})$ to time t_i. We then matched the inferred node time quantile distribution with the target node time quantile distribution using linear interpolation on the quantiles. We used this mapping to rewrite all nodes of time t_i to the new corresponding time.

Theoretical properties

In this section we describe theoretical propeties that motivate the threading instructions used by step 3 of ARG-Needle, as well as a connection between ARG-Needle and ASMC-clust.

Recovery of marginal trees using true coalescence times

We show that using true pairwise coalescence times in the threading instruction recovers correct marginal trees, motivating our choice of step 3 in the algorithm. Consider a position x with a marginal tree over N' samples, and suppose the threading instruction for sample $N' + 1$ has $f(x) = (i, T)$, joining to sample i at time T. Let tmrca(a, b) denote the pairwise TMRCA between samples a and b (position x is implicit). We begin with a few observations (these claims are easily verified, and we omit a proof):

Claim 1. For samples $1 \leq a < b \leq N'$ (samples already in the ARG), tmrca(a, b) is the same before and after the above threading operation.

Claim 2. After the threading operation, tmrca$(N' + 1, i) = T$.

The remaining pairwise TMRCAs after threading can also be determined:

Claim 3. Let j be any sample in the ARG other than i. After the threading operation, we have

$$\text{tmrca}(N' + 1, j) = \max\left(T, \text{tmrca}(i, j)\right).$$

Proof of Claim 3. Let v be the pairwise MRCA node of samples i and j, and w be the pairwise MRCA node of samples $N' + 1$ and i after threading (having time T). There are now three cases:

1. If tmrca$(i, j) < T$, then v lies below w, and there is a path from the node for sample i to w which must pass through v. Therefore w is the MRCA node of j and $N' + 1$, so tmrca$(N' + 1, j) = T = \max(T, \text{tmrca}(i, j))$.

2. If tmrca$(i, j) > T$, then w lies below v. The MRCA of w (a non-leaf node) and sample j is v, and sample $N' + 1$ is a descendant of w, so tmrca$(N' + 1, j) =$ tmrca$(i, j) = \max(T, \text{tmrca}(i, j))$.

3. If tmrca$(i, j) = T$, then when sample $N' + 1$ was being threaded to the sample i, it would have found node v already at time T, leading to the second threading case above and adding a single edge between the node for sample $N' + 1$ and v. Therefore tmrca$(N' + 1, j) = T = \max(T, \text{tmrca}(i, j))$.

□

We leverage these observations to verify that the threading algorithm reconstructs the true ARG when the threading instructions are correct (i.e., when at each position the threading instruction provides a true closest relative and their true TMRCA to the target sample).

Theorem. Let $\mathcal{A}$ be an ARG over N samples, and let $\mathcal{B}$ be an ARG constructed via threading from an initially trivial ARG with one sample. Suppose that for threading sample N', one uses the threading instruction $f(x) = (i(x), T(x))$ consisting of $i(x) = \text{argmin}_{1\leq i'<N'}(\text{tmrca}_{\mathcal{A},x}(N', i'))$ and $T(x) = \min_{1\leq i'<N'}(\text{tmrca}_{\mathcal{A},x}(N', i'))$. Then at each x, the marginal trees of $\mathcal{A}$ and $\mathcal{B}$ will be identical (not considering the possible creation or deletion of unary nodes).

Proof of Theorem. It suffices to consider an arbitrary position x and show that the marginal trees of $\mathcal{A}$ and $\mathcal{B}$ are equivalent. We therefore rely on the fact that the full set of TMRCAs uniquely determines a rooted tree [26], and show that the $\binom{N}{2}$ pairwise TMRCAs between any two samples are the same within $\mathcal{A}$ and $\mathcal{B}$. We proceed by induction, and assume that the pairwise TMRCAs between the first N' samples are the same in $\mathcal{A}$ and $\mathcal{B}$. The pairwise TMRCAs between $N'+1$ and the first N' samples are set when sample $N'+1$ is threaded to $\mathcal{B}$, and unchanged after (by Claim 1). Let i and T denote the chosen sample and time as defined above, then by Claim 2, $\text{tmrca}_{\mathcal{B}}(N'+1, i) = T = \text{tmrca}_{\mathcal{A}}(N'+1, i)$. It remains to show that $\text{tmrca}_{\mathcal{B}}(N'+1, j) = \text{tmrca}_{\mathcal{A}}(N'+1, j)$ for any $j \neq i$ among the first N' samples. We have

$$\begin{aligned}\text{tmrca}_{\mathcal{B}}(N'+1, j) &= \max\left(T, \text{tmrca}_{\mathcal{B}}(i, j)\right)\\ &= \max\left(T, \text{tmrca}_{\mathcal{A}}(i, j)\right),\end{aligned}$$

by Claim 3 and the inductive hypothesis. By the definition of T,

$$\begin{aligned}\text{tmrca}_{\mathcal{B}}(N'+1, j) &= \max\left(T, \text{tmrca}_{\mathcal{A}}(i, j)\right)\\ &\leq T\\ &= \min_{1\leq i'\leq N'}(\text{tmrca}_{\mathcal{A}}(N'+1, i'))\\ &\leq \text{tmrca}_{\mathcal{A}}(N'+1, j).\end{aligned}$$

However, by the ultrametric property,

$$\begin{aligned}\text{tmrca}_{\mathcal{B}}(N'+1, j) &= \max\left(T, \text{tmrca}_{\mathcal{A}}(i, j)\right)\\ &= \max\left(\text{tmrca}_{\mathcal{A}}(N'+1, i), \text{tmrca}_{\mathcal{A}}(i, j)\right)\\ &\geq \text{tmrca}_{\mathcal{A}}(N'+1, j).\end{aligned}$$

Putting these together, $\text{tmrca}_{\mathcal{B}}(N'+1, j) = \text{tmrca}_{\mathcal{A}}(N'+1, j)$. □

Relationship between ASMC-clust and ARG-Needle under ultrametric TMRCAs

We highlight an additional property that provides a connection between the ARG-Needle and ASMC-clust algorithms. Assuming that ASMC returns ultrametric distances for every position (i.e., they are

non-negative, symmetric, and for samples a, b, c they satisfy $d(a,b) \leq \max(d(a,c), d(c,b))$), then, by running UPGMA, ASMC-clust is guaranteed to recover an ARG $\mathcal{A}$ with marginal trees at each position exactly reflecting these distances. By the earlier theorem (and assuming sufficiently accurate hashing), applying ARG-Needle to the same pairwise TMRCA distances will lead to reconstructing an ARG with the same marginal trees as $\mathcal{A}$. Thus, in the specific setting of ultrametric TMRCA estimates, ASMC-clust and ARG-Needle output ARGs with identical marginal trees. Furthermore, this is true regardless of the order in which samples are threaded using ARG-Needle, so the ARG-Needle marginal trees are unaffected by the order of threading.

In practice, TMRCAs output by ASMC will deviate from being ultrametric. In this case, ASMC-clust will build ARGs with marginal trees where the deviation with respect to ASMC distances is jointly minimized for all samples, whereas ARG-Needle will consider the minimum distance between a single sample and the subset of samples present in the ARG at a given iteration. This is in line with the observation that the ARG-Needle algorithm resembles ASMC-clust in terms of inferred marginal trees (see e.g., Fig. 2 and Supplementary Fig. 3), but is computationally more efficient. Future work may provide further understanding of the differences between ARGs inferred using ARG-Needle and ASMC-clust, or using ARG-Needle with different orders of threading (see e.g., Supplementary Fig. 5), in terms of the degree to which utilized TMRCA estimates diverge from being ultrametric.

Supplementary Note 2: Additional details about ARG evaluation metrics

We studied four specific metrics to compare true and inferred ARGs: Robinson-Foulds distance, ARG total variation distance, pairwise TMRCA RMSE, and Kendall-Colijn (KC) distance. We also looked at scatter plots of the predicted vs. true TMRCAs. We first describe our use of stabbing queries to calculate these metrics, then discuss the various evaluation methods in more detail.

Evaluating metrics via stabbing queries

Most of the metrics we considered—Robinson-Foulds distance, pairwise TMRCA RMSE, and KC distance—are originally defined on two trees. (The ARG total variation distance is defined directly between two ARGs and is treated separately.) To generalize these metrics to ARGs, we consider the metrics as comparing two marginal trees at the same position and take a genome-wide average of the metrics over all positions.

Consider two ARGs $\mathcal{A}$ and $\mathcal{B}$, which may for instance be the true and inferred ARGs, with a common genomic extent $[s, t) \subset \mathbb{R}$. (Note that we are modeling the genome as continuous.) Let Tree be a binary operator that takes an ARG and a position and returns the tree at that position, and suppose $d : \mathcal{T}_N \times \mathcal{T}_N \to \mathbb{R}$ is a metric of interest that operates on the space $\mathcal{T}_N$ of rooted trees on N leaves. Then the genome-wide average of d is

$$\frac{1}{t-s}\int_s^t d(\text{Tree}(\mathcal{A}, x), \text{Tree}(\mathcal{B}, x))dx.$$

Although it is possible to compute such integrals exactly, we found that such a method is slowed down by needing to iterate over all marginal trees in both ARGs. In particular, the true ARG often contains many recombination events, each of which generates a new marginal tree. Therefore, we instead approximated the integral using sampling. Given a set of points $x_1, \ldots, x_n$ uniformly distributed among $[s, t)$, we have

$$\frac{1}{t-s}\int_s^t d(\text{Tree}(\mathcal{A}, x), \text{Tree}(\mathcal{B}, x))dx \approx \frac{1}{n}\sum_{i=1}^{n} d(\text{Tree}(\mathcal{A}, x_i), \text{Tree}(\mathcal{B}, x_i)).$$

We call each such $d(\text{Tree}(\mathcal{A}, x_i), \text{Tree}(\mathcal{B}, x_i))$ term a "stabbing query", because we are sampling two trees at a position along the ARG, which entails finding the edges of the ARG that overlap this position. The estimate is then an unweighted average over n stabbing queries. For choosing the points $\{x_i\}_{i=1}^n$, we set $x_i = s + (i \cdot \phi - \lfloor i \cdot \phi \rfloor) \times (t - s)$, where $\phi = (1 + \sqrt{5})/2$ is the golden ratio. For all evaluations, we used $n =$ 5,000 stabbing queries.

ARG total variation distance as a generalization of the Robinson-Foulds distance

In Methods, we defined the scaled Robinson-Foulds distance, which for two binary trees with N samples is obtained by dividing by the maximum possible value of $2N - 4$. We average this metric over $n =$ 5,000 genome-wide stabbing queries to obtain a quantity between 0 (perfect match of present mutations) and 1 (complete mismatch of non-singleton mutations).

The Robinson-Foulds distance weights all possible mutations in a marginal tree equally when considering dissimilarity. However, rare variants in these marginal trees tend to correspond to recent, short branches and are less likely to occur through random mutations. Furthermore, these short and recent branches tend to represent long haplotypes and thus appear in multiple neighboring trees, leading to a disproportionate contribution to the overall distance. The ARG total variation distance generalizes the Robinson-Foulds distance to overcome these limitations when comparing ARGs.

To define the ARG total variation distance, we consider the probability distribution of possible mutations encoded by an ARG, and compute the total variation distance between two such distributions. More formally, given N samples, the possible non-trivial mutational patterns that can occur over these samples are given by the set $\{0,1\}^N \setminus \{(0,\ldots,0),(1,\ldots,1)\}$. We call each element of this set an N-bitset; there are $2^N - 2$ possible N-bitsets, which we denote as $\mathcal{S}_N$. We choose to represent each mutation using its position $x \in [s,t)$ and the N-bitset $b \in \mathcal{S}_N$. We thus consider the probability distribution corresponding to the mutations $(x,b) \in [s,t) \times \mathcal{S}_N$ that can be observed. Assuming a constant mutation rate, each simulated mutation is uniformly distributed over the area of the ARG, which consists of the physical distance times the height of each mutation-generating branch. We can capture such a distribution via a probability density function (PDF) $f_{\mathcal{A}} : [s,t) \times \mathcal{S}_N \to \mathbb{R}$ which is induced by uniform sampling over the ARG. We can use two equivalent expressions for the total variation distance and apply them to compute the total variation distance between two ARGs $\mathcal{A}$ and $\mathcal{B}$ in terms of their PDFs:

$$TV_{ARG}(\mathcal{A},\mathcal{B}) = \frac{1}{2}\int_s^t \sum_{b\in\mathcal{S}_N} |f_{\mathcal{A}}(x,b) - f_{\mathcal{B}}(x,b)|dx \tag{3}$$

$$= 1 - \int_s^t \sum_{b\in\mathcal{S}_N} \min(f_{\mathcal{A}}(x,b), f_{\mathcal{B}}(x,b))dx. \tag{4}$$

The distance is 0 for identical distributions and 1 if the two distributions contain no overlapping support.

To evaluate the ARG total variation distance in our simulations, we used the second expression, where we approximated the integral by sampling $n =$ 5,000 stabbing queries and performed a sum over all present mutations for each stabbing query. We benchmarked the various methods across our simulation conditions with and without ARG normalization. Overall, ARG normalization either kept the total variation distance the same or provided a modest benefit (Extended Data Figs. 2b and 3b for methods without ARG normalization, and Extended Data Figs. 2c and 3c for methods with ARG normalization).

To highlight the connection between the ARG total variation distance and Robinson-Foulds more explicitly, we first describe how we compute the PDF $f_{\mathcal{A}}(x,b)$ of an ARG $\mathcal{A}$. The value $f_{\mathcal{A}}(x,b)$ should be 0 if no branch yielding N-bitset b exists at the tree at position x. Otherwise, $f_{\mathcal{A}}(x,b)$ should be proportional to the length of the branch yielding b. Define a function $l_{\mathcal{A}} : [s,t) \times \mathcal{S}_N \to \mathbb{R}$ which will be an unnormalized version of $f_{\mathcal{A}}(x,b)$:

$$l_{\mathcal{A}}(x,b) = \begin{cases} \text{the length of a branch giving } b \text{ at Tree}(\mathcal{A},x), & \text{if one exists,} \\ 0, & \text{otherwise.} \end{cases} \tag{5}$$

We can then write

$$f_{\mathcal{A}}(x,b) = \frac{1}{Z_{\mathcal{A}}} l_{\mathcal{A}}(x,b) \tag{6}$$

for some scaling constant $Z_{\mathcal{A}}$. We would like all the properties of a PDF to hold, e.g.,

$$\int_s^t \sum_{b \in \mathcal{S}_N} f_{\mathcal{A}}(x,b)dx = 1. \tag{7}$$

Combining (6) and (7), we obtain

$$Z_{\mathcal{A}} = \int_s^t \sum_{b \in \mathcal{S}_N} l_{\mathcal{A}}(x,b)dx. \tag{8}$$

For a given ARG $\mathcal{A}$, equations (5), (6), and (8) provide a way to evaluate its PDF. First, we compute $Z_{\mathcal{A}}$ using stabbing queries. Then, to get the value of $f_{\mathcal{A}}(x,b)$, we look up $l_{\mathcal{A}}(x,b)$ using the marginal tree at x, and divide by $Z_{\mathcal{A}}$.

Consider again the definition of $l_{\mathcal{A}}(x,b)$. We can obtain a topology-only version of the ARG total variation distance by rewriting $l_{\mathcal{A}}(x,b)$ to not use branch lengths:

$$l'_{\mathcal{A}}(x,b) = \begin{cases} 1, & \text{if a branch giving } b \text{ exists at Tree}(\mathcal{A}, x), \\ 0, & \text{otherwise.} \end{cases}$$

If we use the same definitions for TV_{ARG} but replace $l_{\mathcal{A}}(x,b)$ with $l'_{\mathcal{A}}(x,b)$ and assume no polytomies in $\mathcal{A}$ or $\mathcal{B}$, the ARG total variation distance reduces to a scaled version of the Robinson-Foulds metric.

Previous metrics have been proposed that generalize the Robinson-Foulds distance to consider branch lengths [27, 28]. Using our notation, and assuming we average these metrics across the genome, the weighted Robinson-Foulds distance [27] is

$$WRF_{ARG}(\mathcal{A},\mathcal{B}) = \frac{1}{t-s} \int_s^t \sum_{b \in \mathcal{S}_N} |l_{\mathcal{A}}(x,b) - l_{\mathcal{B}}(x,b)| dx \tag{9}$$

and the Kuhner-Felsenstein distance [28] is

$$KF_{ARG}(\mathcal{A},\mathcal{B}) = \frac{1}{t-s} \int_s^t \sum_{b \in \mathcal{S}_N} \left(l_{\mathcal{A}}(x,b) - l_{\mathcal{B}}(x,b)\right)^2 dx. \tag{10}$$

We particularly highlight the similarity between our first expression for the ARG total variation distance (3) and the weighted Robinson-Foulds distance (9). In the weighted Robinson-Foulds distance, branch lengths are compared directly, whereas in the ARG total variation distance, branch lengths are normalized by the area of the entire ARG to obtain probability distributions which are then compared. This normalization facilitated an interpretation in terms of mutations that may be sampled from an ARG, and results in values between 0 and 1.

It is also possible to stratify the ARG total variation distance by allele frequency. In our case, we considered various minor allele count (MAC) ranges and limited the probability distribution of each

ARG to those mutations falling within the MAC range (see Supplementary Fig. 2). This affects the computations by modifying (5) to:

$$l_{\mathcal{A}}(x,b) = \begin{cases} \text{the length of a branch giving } b \text{ at Tree}(\mathcal{A},x) \text{ with desired MAC}, & \text{if one exists,} \\ 0, & \text{otherwise.} \end{cases} \tag{11}$$

We briefly comment on limitations of the ARG total variation distance. Note that in our definition, we considered the space of possible mutations as consisting of a position $x \in [s,t)$ and an N-bitset $b \in \mathcal{S}_N$. Including the position enables us to measure the ability to correctly localize mutations in the genome. It may be worthwhile to also consider the time (height) of the mutations, which would measure the ability to correctly localize the time of mutation events. Additionally, the ARG total variation distance relies on a hard 0-1 loss in its definition: it does not consider whether two mutations are correlated (with small Hamming distance) or occur at close but disjoint positions in two ARGs, a limitation it shares with the Robinson-Foulds distance. It may be possible to instead use the Wasserstein distance, which generalizes the total variation distance with the aid of a metric on the probability space, thus allowing "margin for error" with a smooth loss.

Pairwise TMRCA RMSE and TMRCA scatter plots

For pairwise TMRCA comparisons, at each position we compute the TMRCA of each pair of samples in the true ARG and the inferred ARG, resulting in two vectors of length $N(N-1)/2$. We compute the average mean squared error between these vectors over all positions, then take the square root to yield a pairwise TMRCA root mean squared error (RMSE). When averaging over varying orders of threading (Supplementary Fig. 5d), we average the length $N(N-1)/2$ vectors.

We provide additional details on the interpretation of TMRCA RMSE. Recall that we introduced ARG normalization as a way to adjust the node times of an inferred ARG to be more consistent with a demographic prior. We observed that ARG normalization improves the pairwise TMRCA RMSE of Relate in array data, though not in sequencing data (compare Extended Data Fig. 2d,f), and tends to improve performance for tsinfer as well. This suggests that ARG normalization provides a reasonable branch length estimation heuristic when branch lengths are not modeled or inferred under model misspecification, as in the case of Relate on array data. For ARG-Needle and ASMC-clust, ARG normalization improves the overall calibration of TMRCAs by making the range of predicted coalescence times closer to that expected from the demographic prior (see Extended Data Fig. 2a-d). Interestingly, however, ARG normalization decreases the TMRCA RMSE performance of ARG-Needle and ASMC-clust in both array and sequencing data (compare Extended Data Fig. 2d-e), such that ARG-Needle and ASMC-clust without ARG normalization consistently achieve the best TMRCA RMSE across methods (Extended Data Fig. 2e). This is likely linked to our use of ASMC's posterior mean TMRCA estimator, which is biased towards the average prior TMRCA but leads to good RMSE performance due to the weight placed by an L2 norm on outliers. ARG normalization is also likely to reduce the accuracy in the root node height for ARGs built using ARG-Needle and ASMC-clust. This

may have a large impact on TMRCA RMSE as a large fraction of pairwise coalescence events involve the root.

It may be worthwhile to develop additional metrics that incorporate pairwise TMRCA values without being as influenced by the root event or large values, for instance using an L1 norm instead of an L2 norm, or by measuring the RMSE of log TMRCA or of the square root of TMRCA. We note, however, that accuracy on the pairwise TMRCA RMSE is connected to several analyses discussed in this work in the context of ARG-GRMs. In particular, increased performance for pairwise TMRCA RMSE reflects increased similarity (under the Frobenius norm) between true and inferred ARG-GRMs (when we assume $\alpha = 0$, see Methods).

KC distance

In their work on the KC distance [29], Kendall and Colijn introduced both topology-only and branch-length-aware versions of the KC distance. We primarily focused on the topology-only ($\lambda = 0$) version of the KC distance. However, we briefly note that we also performed some simulations with $\lambda = 1$ and $\lambda = 0.02$ (Supplementary Fig. 1). The $\lambda = 1$ KC distance compares aggregate branch lengths between pairwise MRCA events and root times, while values of λ between 0 and 1 weight both topology and branch length measurements. The value of $\lambda = 0.02$ was chosen to balance the contribution of branch lengths and topologies, based on a median branch length of 49 generations in CEU coalescent simulations of $N = 4{,}000$ individuals (see [29] Supplementary Information 1.2).

We now focus on discussing the $\lambda = 0$ Kendall-Colijn distance. The Kendall-Colijn topology-only distance [29] (henceforth "KC distance" for short) has been used to evaluate inferred ARGs (e.g., in [14]). As a topology-only metric, it does not consider branch length or coalescence time information. Instead, it counts the number of nodes that occur on the path from a desired MRCA node to the root node, and compares these counts for all possible MRCA pairs. Given two marginal trees T_1 and T_2, each with the same set of samples labeled 1 to N, the KC distance computation first calculates a vector of length $N(N-1)/2$ for each tree as follows. For one of the trees T, let $\text{root}(T)$ denote the root node of the tree, and for any two nodes a and b in the tree, let $\text{mrca}_T(a, b)$ denote the most recent common ancestor of a and b and let $\text{dist}_T(a, b)$ denote the tree-path distance between nodes a and b in the tree, defined as the number of edges in the tree that need to be traversed to travel between a and b. For any two distinct samples $1 \leq i < j \leq N$, let $\text{leaf}_T(i)$ and $\text{leaf}_T(j)$ denote the corresponding leaf nodes. We consider the MRCA of the two leaf nodes and count the distance from the root of the tree, computing

$$m_T(i, j) = \text{dist}_T\left(\text{root}(T), \text{mrca}_T(\text{leaf}_T(i), \text{leaf}_T(j))\right).$$

We then form vectors

$$\mathbf{m}_{T_1} = \left(m_{T_1}(1,2), m_{T_1}(1,3), \ldots, m_{T_1}(N-1, N)\right),$$
$$\mathbf{m}_{T_2} = \left(m_{T_2}(1,2), m_{T_2}(1,3), \ldots, m_{T_2}(N-1, N)\right).$$

(Note that in [29] the $\mathbf{m}_T$ vectors are augmented with additional entries, which are however irrelevant in our case.) Finally, we define the KC distance as the L2 norm between these two length $N(N-1)/2$ vectors:

$$\text{KC}(T_1, T_2) = ||\mathbf{m}_{T_1} - \mathbf{m}_{T_2}||_2 = \left(\sum_{1 \leq i < j \leq N} (m_{T_1}(i,j) - m_{T_2}(i,j))^2 \right)^{1/2} .$$

One option to combine tree-wise KC distances to get a comparison between ARGs is to weight the KC metric by the genomic distance spanned by each tree, as done in [14]. For efficiency, this can be approximated by taking an unweighted average of the KC metric for several stabbing queries. In this work we opted to instead average the squared KC distance over the stabbing queries, then perform the square root (similar to our TMRCA RMSE calculations). A benefit of this approach is that it preserves the interpretation of the (ARG) KC distance as an L2 norm. (Suppose $\mathbf{m}_{1x}$ and $\mathbf{m}_{1y}$ are vectors from the first ARG at two locations x and y, and $\mathbf{m}_{2x}$ and $\mathbf{m}_{2y}$ are vectors from the second ARG at two locations. Assume both locations receive a weight of $1/2$. Then our metric is equivalent to computing $||((\mathbf{m}_{1x}, \mathbf{m}_{1y}) - (\mathbf{m}_{2x}, \mathbf{m}_{2y}))/2||_2$, whereas the other approach results in $(||\mathbf{m}_{1x} - \mathbf{m}_{2x}||_2 + ||\mathbf{m}_{1y} - \mathbf{m}_{2y})||_2)/2$.)

The KC distance is affected whenever two arbitrarily close coalescence events are joined to form a polytomy, or when polytomies are broken to form strictly bifurcating trees, since these operations create or remove nodes and thus alter the distances from internal nodes to the root. We performed experiments in which we resolved polytomies in marginal trees produced by tsinfer and tsinfer-sparse, replicating the approach used in [14]. At each polytomy with k child edges coalescing, a random binary tree with k leaves was generated and was substituted in place of the polytomy. In Fig. 2d, Extended Data Figs. 1b-c and 3f-g, and Supplementary Fig. 4f-g, we show results for tsinfer and tsinfer-sparse with polytomies as well as with polytomies broken, where we sample the breaking procedure 10 times and average the KC results. As in [14], we observed that randomly resolving polytomies in tsinfer leads to worse performance on the KC distance.

We compared all methods on the KC distance with any polytomies randomly resolved (Extended Data Figs. 1d and 3h and Supplementary Fig. 4h), but also sought to compare against tsinfer and tsinfer-sparse while allowing polytomies. In [14], the better performance of ARGs containing polytomies has been attributed to improved modeling of uncertainty, as polytomies are produced by tsinfer when insufficient information is available to resolve the nodes. ARG-Needle, ASMC-clust, and Relate do not output trees with polytomies, but the estimated branch lengths in these trees may provide some information as to the uncertainty on the existence of a branch. For instance, if the height spanned by a branch is shorter than the uncertainty of parent and child node times, the branch may be instead represented as a polytomy. We therefore developed a simple heuristic that aims to approximately quantify the uncertainty associated with inferred marginal tree branches. This heuristic orders the non-leaf branches in each inferred binary tree by computing the ratio of the branch length divided by the height of the parent node (to take into account that coalescent events in distant time are estimated

with more uncertainty). We select the fraction f of the $N-2$ non-leaf branches with smallest such ratio and collapse these to form polytomies. We also implemented an approach to randomly merge nodes to form polytomies, where we collapse each non-leaf branch with independent probability f. Thus, both merging operations take a real parameter f between 0 and 1 corresponding to the fraction of non-leaf branches that are collapsed in each marginal tree of the inferred ARG. For both methods of merging, we use a single merged tree per site and evaluate the KC distance against the marginal tree in the true ARG.

We tested values $f \in \{0, 0.05, 0.1, 0.2, 0.3, 0.4, 0.5, 0.75\}$, and measured the KC distance after applying merging for ARGs inferred by Relate, ASMC-clust, and ARG-Needle (Extended Data Figs. 1b-c and 3f-g and Supplementary Fig. 4f-g). In both sequencing and array data, Relate, ASMC-clust, and ARG-Needle achieved lower KC distance when nodes were merged to form polytomies, including when the random merging strategy was used, suggesting that the KC distance is systematically lower for inferred trees that contain polytomies. Using heuristic merging in array data led to Relate, ASMC-clust, and ARG-Needle performing better than tsinfer and tsinfer-sparse at the optimal merging fraction (Extended Data Fig. 1b), while in sequencing data these methods matched tsinfer at the optimal merging fraction (Extended Data Fig. 3f). We did not observe the ordering of methods to change when we allowed tsinfer and tsinfer-sparse to form additional polytomies with random merging, and we did not apply our heuristic merging strategy to tsinfer and tsinfer-sparse due to a lack of branch lengths.

Although this heuristic provides a computationally efficient approach to translate length-based uncertainty into polytomy-based uncertainty, it is likely to be suboptimal, implying that there may exist other strategies that improve the performance of ARG-Needle, ASMC-clust, and Relate in these experiments. We also note that the heuristic was applied to ARG-Needle and ASMC-clust before the ARG normalization step, though we expect similar results if ARG normalization is included. Finally, note that we did not use this strategy in downstream applications, where we instead directly relied on branch lengths, e.g., by sampling mutations proportionally to branch areas.

We briefly speculate on why collapsing branches to form polytomies, including at random, may improve performance on the KC distance. The KC distance compares trees by computing the L2 norm between the two vectors $\mathbf{m}_{T_1}$ and $\mathbf{m}_{T_2}$, which will penalize large differences. Take T_1 to be the true tree and T_2 to be the inferred tree. In our coalescent simulations, T_1 is a relatively well-balanced binary tree, so the values of $\mathbf{m}_{T_1}$ will range from 0 (a pair of samples that coalesces at the root) to $O(\log N)$, with a mode and mean of $O(1)$ (as a large fraction of pairs coalesce at the root). The introduction of polytomies may result in shrinkage of the values of $\mathbf{m}_{T_2}$, because the paths from MRCA nodes to the root contain fewer intermediate nodes, introducing bias but reducing variance. This may reduce the overall MSE, as seen in other shrinkage estimators such as ridge regression. Furthermore, while all tree comparison metrics are affected by the balance and shape of the underlying trees, the KC distance has been shown to be particularly sensitive [30].

Overall, these experiments provide several insights into the tested algorithms and the use of the topology-only KC distance for the comparison of ARGs. Because the KC distance may take systematically lower values for trees containing polytomies, including when these are formed at random (Extended Data Figs. 1c and 3g), care should be adopted when comparing ARGs that contain polytomies with ARGs where polytomies are fully resolved. Furthermore, forming polytomies to account for uncertainty, including through strategies that rely on estimated branch lengths (Extended Data Figs. 1b and 3f), allows methods to substantially improve on the KC distance. Finally, although fully resolving polytomies is likely to lead to suboptimal performance, this type of analysis may provide additional insights (Extended Data Figs. 1b-d and 3f-h). For instance, although ARG-Needle and ASMC-clust achieve a similar optimal performance when polytomies are formed, ARG-Needle's performance decreases more rapidly as the amount of polytomies is restricted (Extended Data Fig. 1b,d). Additional work may highlight further connections between the KC distance and properties of inferred ARGs that perform well under this metric, including their performance in downstream analyses.

Supplementary Note 3: Additional details on ARG-GRMs

In Methods, we introduced ARG-GRMs for the case of haploid samples and $\alpha = 0$, as well as Monte Carlo ARG-GRMs, which sample new mutations on the ARG and use those markers to construct GRMs. Monte Carlo ARG-GRMs enable easily taking into account diploid samples, modeling varying values of α, and working with stratified ARG-GRMs. In this Note, we start by providing additional details of our simulation analyses. We then elaborate on the derivations behind ARG-GRMs, deriving an exact ARG-GRM for the cases of diploid samples, general α, and stratification. We also discuss various invariances of the GRM used in mixed-model analysis and show how all methods compute an expected version of the sequence-based GRM up to invariance.

Additional experimental details for ARG-GRMs

We compared the use of Monte Carlo ARG-GRMs to exactly computed ARG-GRMs for the case $\alpha = 0$, verifying that the Monte Carlo ARG-GRM results saturated and converged to those of the exact ARG-GRM for large mutation rates (Extended Data Fig. 7b-c). We therefore used a value of $\mu \approx 1.65 \times 10^{-7}$ for all other experiments.

For simulations of complex trait analysis, we computed GRMs using ARGs, SNP data, imputed data, and sequencing data and provided them in input to GCTA with the simulated phenotype. For heritability estimation, we ran GCTA with flags `--reml-no-constrain` and `--reml-no-lrt`, and utilized both the estimated heritability and estimated standard errors within a meta-analysis over 5 independent simulations. For polygenic prediction, we ran leave-one-out prediction using cvBLUP [31] within GCTA, then computed r^2 between the resulting predictions and the phenotype. For ARG-GRM association experiments (Fig. 3c and Supplementary Fig. 8c,f), we performed MLMA of array data SNPs, testing each chromosome while using a LOCO GRM built on the other 21 chromosomes. We measured power improvement as the relative increase of mean $-\log_{10}(p)$ for MLMA compared to linear regression of array data SNPs and compared ARG-GRMs to GRMs of array and sequencing data.

In most experiments involving ARG-GRMs of true ARGs (Fig. 3c, Extended Data Figs. 7b-c, 8a-f), we used the true value of α leveraged in trait simulation to build GRMs (see (12) below). For simulations adopting MAF-stratification, as a proof-of-concept (Extended Data Fig. 8g) and when dealing with inferred ARGs (Fig. 3b and Supplementary Fig. 8), we used MAF boundaries given by $\{0, 0.01, 0.05, 0.5\}$, normalized genotypes using $\alpha = -1$, and then provided the MAF-stratified ARG-GRMs (see (13) below) in input to GCTA [32]. In Fig. 3b and Supplementary Fig. 8, we applied the same MAF-stratification to imputed data, but not to SNP data, for which GCTA did not converge. For the experiments of Supplementary Fig. 5e-f, we compared $\alpha = 0$ Monte Carlo ARG-GRMs in terms of Frobenius norm for true ARGs, ASMC-clust, ARG-Needle, and an average of ARG-GRMs for ARG-Needle with varying threading orders. As the $\alpha = 0$ GRM can be constructed from pairwise

TMRCAs (Extended Data Fig. 7a), this quantity is closely related to the pairwise TMRCA RMSE between ARGs.

Notation for sequence-based GRMs to ARG-GRMs

To introduce the theory of ARG-GRMs, we first provide notation for the various sequence-based GRMs which we seek to approximate using ARGs. In all cases we have M markers and N individuals. While we have focused on haploid individuals with genotypes $x_{ik} \in \{0, 1\}$, $1 \leq i \leq N$ and $1 \leq i \leq M$, we also describe the case of diploid individuals with genotypes $x_{ik} \in \{0, 1, 2\}$, $1 \leq i \leq N$ and $1 \leq i \leq M$.

We begin by discussing the sequence-based GRM for haploid genotypes and general α, with allele frequencies $p_k = \frac{1}{N}\sum_{i=1}^{N} x_{ik}$:

$$K_{\alpha,hap}(i,j) = \frac{1}{M}\sum_{k=1}^{M} \frac{(x_{ik} - p_k)(x_{jk} - p_k)}{[p_k(1-p_k)]^{-\alpha}}. \tag{12}$$

We then describe stratified GRMs, which are obtained by partitioning the markers into various bins via MAF, LD, time intervals (to capture allele age), or other annotations. The haploid stratified GRM for a bin containing SNPs $B \subseteq \{1, \ldots, M\}$ using value α is given by:

$$K_{\alpha,hap,B}(i,j) = \frac{1}{|B|}\sum_{k=1}^{M} \mathbb{1}_{k\in B}\frac{(x_{ik} - p_k)(x_{jk} - p_k)}{[p_k(1-p_k)]^{-\alpha}}. \tag{13}$$

(We use $\mathbb{1}_A$ to represent the indicator function of event A, taking value 1 if A holds and 0 otherwise.)

Lastly, we consider the general α sequence-based GRM for diploid genotypes, with allele frequencies $p_k = \frac{1}{2N}\sum_{i=1}^{N} x_{ik}$:

$$K_{\alpha,dip}(i,j) = \frac{1}{M}\sum_{k=1}^{M} \frac{(x_{ik} - 2p_k)(x_{jk} - 2p_k)}{[2p_k(1-p_k)]^{-\alpha}}. \tag{14}$$

(We omit a discussion on diploid stratified GRMs, which are easily derived as a generalization of the above two cases.)

From sequence-based GRMs to ARG-GRMs

The above GRM expressions assume we have access to a set of markers. Under the infinite sites assumption, each marker corresponds to an event that occurred at some time in the past. For simplicity, we refer to these events as mutations, though they may also consist of other variant types, such as short indels. Each such mutation occurs somewhere on the ARG, with position x and time t, and affects a set of descendants that will carry the derived allele. In the haploid case, if we let the descendants of a mutation m be $d(m) \subset \{1, \ldots, N\}$, we can also define the allele frequency $p(m)$ of a mutation as $p(m) = |d(m)|/N$.

Assuming a uniform mutation rate and the infinite-sites model, mutations arise uniformly over the area of the ARG as a Poisson process characterized by the mutation rate μ. The area of the ARG consists of all its edges, but an ARG edge does not always have a constant set of descendants, if a recombination event occurs on a path between the edge and one of its descendants. Therefore, we may further partition the edges of the ARG into "branches", such that each branch b is valid for only part of the genomic extent of an edge and carries a constant set of descendants. We let $A(b)$ denote the area of branch b, obtained by multiplying its extent in time (e.g., generations) by its extent along the genome, and assume that the set B enumerates all branches. Each branch b then also inherits a set of descendants $d(b) \subset \{1, \ldots, N\}$ and an allele frequency $p(b)$, just as for mutations. The area of the ARG is then the sum over all these branches, or $\sum_{b \in B} A(b)$.

In the case where we do not have access to all underlying markers needed to compute a GRM but have access to the ground truth ARG or an inferred ARG, we may compute the expectation for the GRM entries. For the haploid single-component ARG-GRM, we compute

$$K_{\alpha,hap}(i,j) = \mathbb{E}_{m \sim Uniform(\{m_1,\ldots,m_M\})} \frac{\left(\mathbb{1}_{i \in d(m)} - p(m)\right)\left(\mathbb{1}_{j \in d(m)} - p(m)\right)}{\left[p(m)\left(1 - p(m)\right)\right]^{-\alpha}}. \tag{15}$$

Since mutations are uniformly distributed over the area of an ARG, we replace the set of known M mutations with the distribution over all possible mutations induced by uniform sampling over the ARG:

$$K_{\alpha,hap,ARG}(i,j) = \mathbb{E}_{m \sim Uniform(ARG)} \frac{\left(\mathbb{1}_{i \in d(m)} - p(m)\right)\left(\mathbb{1}_{j \in d(m)} - p(m)\right)}{\left[p(m)\left(1 - p(m)\right)\right]^{-\alpha}}. \tag{16}$$

(16) reflects the expected value of (15) given the genealogical relationships of the ARG, without observing any markers.

$$K_{\alpha,hap,ARG}(i,j) = \mathbb{E}\left[K_{\alpha,hap}(i,j) | ARG\right]. \tag{17}$$

We can compute the ARG-wide expectation given by (16) using Monte Carlo, adopting a mutation rate μ to uniformly sample M' variants on the ARG. We then use these M' sampled variants to evaluate (16).

We can also take this expectation analytically, by weighting all possible branches of the ARG by their area. Using our earlier notation,

$$\begin{aligned} K_{\alpha,hap,ARG}(i,j) &= \mathbb{E}_{m \sim Uniform(ARG)} \frac{\left(\mathbb{1}_{i \in d(m)} - p(m)\right)\left(\mathbb{1}_{j \in d(m)} - p(m)\right)}{\left[p(m)\left(1 - p(m)\right)\right]^{-\alpha}} \\ &= \frac{1}{\sum_{b \in B} A(b)} \sum_{b \in B} \left[A(b) \cdot \frac{\left(\mathbb{1}_{i \in d(b)} - p(b)\right)\left(\mathbb{1}_{j \in d(b)} - p(b)\right)}{\left[p(b)\left(1 - p(b)\right)\right]^{-\alpha}}\right] \end{aligned} \tag{18}$$

This formulation, however, requires traversing all branches of the ARG and updating N^2 values for each branch, which is less efficient than using the Monte Carlo method with a sufficiently high mutation rate. The Monte Carlo method is also $O(N^2)$ per mutation, but fewer mutations need to be considered compared to the exact ARG-GRM approach of visiting all branches, thus reducing computation by a large constant factor.

The case of stratified GRMs is an extension of the above, where we assign each mutation to different GRMs according to the stratification criteria. In our experiments, this corresponded to selecting the appropriate GRM based on the allele frequency ($p(m)$ or $p(b)$) of each sampled mutation.

For diploid GRMs, each individual genotype x_{ik} is modeled as the sum of two haplotypes. We introduce notation where we consider an ARG of $2N$ haploid samples, numbered 1 to $2N$. Without loss of generality, each individual i consists of haplotypes $2i-1$ and $2i$, for $1 \leq i \leq N$. For shorthand, we define $i_1 = 2i-1$ and $i_2 = 2i$. Then we can rewrite (14) as

$$K_{\alpha,dip}(i,j) = \frac{1}{M}\sum_{k=1}^{M} \frac{\left(x_{i_1k} + x_{i_2k} - 2p_k\right)\left(x_{j_1k} + x_{j_2k} - 2p_k\right)}{\left[2p_k\left(1-p_k\right)\right]^{-\alpha}}. \tag{19}$$

If we denote descendants of a mutation in the ARG as $d(m) \subset \{1,\ldots,2N\}$, and $p(m) = |d(m)|/2N$, the diploid ARG-GRM can be computed using the ARG as

$$\begin{aligned} &K_{\alpha,dip,ARG}(i,j) \\ &\quad= \mathbb{E}_{m\sim Uniform(ARG)} \frac{\left(\mathbb{1}_{i_1\in d(m)} + \mathbb{1}_{i_2\in d(m)} - 2p(m)\right)\left(\mathbb{1}_{j_1\in d(m)} + \mathbb{1}_{j_1\in d(m)} - 2p(m)\right)}{\left[2p(m)\left(1-p(m)\right)\right]^{-\alpha}} \\ &\quad= \frac{1}{\sum_{b\in B} A(b)} \sum_{b\in B}\left[A(b)\cdot\frac{\left(\mathbb{1}_{i_1\in d(b)} + \mathbb{1}_{i_2\in d(b)} - 2p(b)\right)\left(\mathbb{1}_{j_1\in d(b)} + \mathbb{1}_{j_2\in d(b)} - 2p(b)\right)}{\left[2p(b)\left(1-p(b)\right)\right]^{-\alpha}}\right] \end{aligned}$$

The exact ARG-GRM is obtained by iterating over all branches, and a Monte Carlo ARG-GRM is obtained by sampling, as previously described.

In the remainder we derive simpler expressions for ARG-GRMs. We begin by describing three useful invariances of GRMs in mixed-model analysis.

Three GRM invariances

We discuss three invariances under which a GRM may be altered without changing downstream results, which we use to facilitate mixed model analysis using ARG-GRMs and in some of the proofs below. We refer to these invariances as scale invariance, data shift invariance, and constant shift invariance.

The two aspects of the ARG-LMM pipeline that are linked to these invariances are Gower centering and the inclusion of a centering covariate. We first describe Gower centering. Given an N by N GRM K, define the N by N identity matrix I_N, the size N column vector 1_N, and the projection matrix P_N (which is symmetric and idempotent and corresponds to projection onto the subspace orthogonal to 1_N) to be

$$P_N = I_N - \frac{1}{N} 1_N 1_N^T.$$

Then, the Gower centered version of K is defined as

$$C_{Gower}(K) = \frac{N-1}{\mathrm{Tr}(P_N K P_N)} K.$$

To obtain correct heritability estimates for sequence-based GRMs with general α (e.g., (12) above), we Gower centered ARG-GRMs provided in input to GCTA. (In the special case of $\alpha = 0$, the

sequence-based GRM in (12) is approximately Gower centered already, and Gower centering merely multiplies by the factor $(N-1)/N$.) Consider the multiplication of K by any nonzero scalar constant γ:

$$C_{Gower}(\gamma K) = \frac{N-1}{\text{Tr}(P_N(\gamma K)P_N)}\gamma K = \frac{N-1}{\gamma\text{Tr}(P_N K P_N)}\gamma K = C_{Gower}(K).$$

Thus, multiplying a GRM by any nonzero scalar and then applying Gower centering will lead to identical downstream results, which we refer to as *scale invariance* of GRMs.

We next consider the inclusion of a centering covariate. Even when no covariates are specified in an analysis, most software packages for complex trait analysis (e.g., PLINK [33], GCTA [32], BOLT-LMM [34, 35], and BOLT-REML [36]) implicitly or explicitly include a centering covariate. This can be thought of as a length N covariate vector consisting of all 1s. Including this covariate is equivalent to mean-centering the phenotype as well as the genotype vector for each marker. It can also be implemented by applying the projection operator P_N, defined above, to project out the component parallel to 1_N in the data.

In our analyses involving GCTA, we provided the GRM and a phenotype in input, rather than providing markers from which to compute the GRM. In this case, the phenotype is mean-centered, and although one does not have access to the underlying genotypes, the centering covariate is still applied implicitly during the relevant mixed-model calculations.

We also describe the inclusion of a centering covariate as a transformation on the GRM itself. In our analyses we performed this operation, which we call data centering, prior to Gower centering, and after both steps were completed, we provided the transformed GRM in input to GCTA. The data centered GRM is given by

$$C_{Data}(K) = P_N K P_N.$$

Right-multiplying by P_N corresponds to subtracting the mean column of a matrix from each column, and left-multiplying by P_N corresponds to subtracting the mean row of a matrix from each row. The transformations C_{Gower} and C_{Data} commute, so that the order in which they are applied does not matter, and that they are both idempotent, so that data centering outside of GCTA does not interfere with the application of the centering covariate inside.

We highlight the second invariance of GRMs we leveraged in these analyses using an example of the effects of data centering. Consider the GRM that would arise if we used raw genotypes instead of centered genotypes in (12):

$$\widetilde{K}_{\alpha,hap}(i,j) = \frac{1}{M}\sum_{k=1}^{M}\frac{(x_{ik})\,(x_{jk})}{[p_k\,(1-p_k)]^{-\alpha}}.$$

We verify that data centering gives the expression (12) with centered genotypes. Notice that the GRM $\widetilde{K}_{\alpha,hap}$ can be written as a product of matrices:

$$\widetilde{K}_{\alpha,hap} = XDX^T$$

where X is of size N by M with entries x_{ij}, and D is a diagonal matrix of size M by M with diagonal entries

$$d_{kk} = [p_k (1 - p_k)]^{\alpha} / M.$$

The data centered version of $\widetilde{K}_{\alpha,hap}$ is then

$$C_{Data}\left(\widetilde{K}_{\alpha,hap}\right) = P_N \widetilde{K}_{\alpha,hap} P_N = P_N(XDX^T)P_N = (P_N X)D(P_N X)^T.$$

Also notice that

$$P_N X = \left(I_N - \frac{1}{N} 1_N 1_N^T\right) X = X - 1_N \left(\frac{1}{N} 1_N^T X\right) = X - 1_N \mu^T$$

where $\mu^T = (p_1, p_2, \ldots, p_M)$ is a row vector consisting of the average row of X, or the collection of allele frequencies. (So that left-multiplying by P_N corresponds to subtracting the mean row of a matrix from each row.) Hence,

$$C_{Data}\left(\widetilde{K}_{\alpha,hap}\right) = (X - 1_N \mu^T) D (X - 1_N \mu^T)^T.$$

This is equivalent to forming a GRM using the centered data, and hence $C_{Data}(\widetilde{K}_{\alpha,hap}) = K_{\alpha,hap}$.

The invariance highlighted in this example, where centered data is replaced by raw genotypes, is more general. Rather than using raw genotypes, we can add a marker-specific constant to the genotypes for each marker before multiplying:

$$\widehat{K}_{\alpha,hap}(i,j) = \frac{1}{M} \sum_{k=1}^{M} \frac{(x_{ik} + c_k)(x_{jk} + c_k)}{[p_k (1 - p_k)]^{-\alpha}},$$

where the c_k are any real scalars for $1 \leq k \leq M$. If we let $\rho^T = (c_1, \ldots, c_M)$, we see that

$$\widehat{K}_{\alpha,hap} = (X + 1_N \rho^T) D (X + 1_N \rho^T)^T.$$

We have

$$\begin{aligned}
P_N(X + 1_N \rho^T) &= P_N X + P_N 1_N \rho^T \\
&= (X - 1_N \mu^T) + \left(I_N - \frac{1}{N} 1_N 1_N^T\right) 1_N \rho^T \\
&= (X - 1_N \mu^T) + \left(1_N - \frac{1}{N} 1_N N\right) \rho^T \\
&= X - 1_N \mu^T.
\end{aligned}$$

Hence

$$C_{Data}\left(\widehat{K}_{\alpha,hap}\right) = P_N \widehat{K}_{\alpha,hap} P_N = (X - 1_N \mu^T) D (X - 1_N \mu^T)^T = K_{\alpha,hap}.$$

We refer to this invariance as *data shift invariance*: by using a centering covariate, the markers used to construct a GRM can have a constant shift per marker applied to the genotypes before multiplying, without affecting downstream results. Although we have only described a detailed derivation in the

case of haploid GRMs, the same considerations apply to other cases, starting from (13) and (14). Note that this form of invariance was also discussed in [37], Appendix B.

Finally, *constant shift invariance* allows adding or subtracting a constant scalar to each entry of a GRM without affecting downstream results. This invariance also follows from the inclusion of a centering covariate. Assume data centering and consider adding a constant c to each entry of a GRM K:

$$\begin{aligned}
C_{Data}(K + c1_N 1_N^T) &= P_N (K + c1_N 1_N^T) P_N \\
&= C_{Data}(K) + \left(I_N - \frac{1}{N} 1_N 1_N^T\right) (c1_N 1_N^T) \left(I_N - \frac{1}{N} 1_N 1_N^T\right) \\
&= C_{Data}(K) + c\left(1_N 1_N^T - 1_N 1_N^T - 1_N 1_N^T + 1_N 1_N^T\right) \\
&= C_{Data}(K).
\end{aligned}$$

In our experiments, we applied data centering directly on the GRM before passing into GCTA, which guaranteed constant shift invariance. We observed that if we omitted the data centering transformation, only relying on GCTA's implementation of a centering covariate, constant shift invariance still held for a range of shift values. However, some experiments involved adding a large negative shift to a GRM such that it is no longer positive semidefinite, and we observed that passing such a GRM directly into GCTA led to errors. On the other hand, we found that GCTA was robust to the earlier described data shift invariance, including when we omitted the data centering transformation, possibly because data shift transformations preserve positive definiteness.

In summary, by always applying Gower centering and what we referred to as data centering to our GRMs, before passing into GCTA, allowed us to guarantee three invariances in the GRM: scale invariance, where the GRM is multiplied by a non-zero scalar; data shift invariance, where a marker-specific shift is applied to each marker used to compute the GRM; and constant shift invariance, where a constant scalar is added to each entry of the GRM.

Exact ARG-GRM, haploid and general α

We use these invariances to describe simplified expressions for computing exact ARG-GRMs. Due to data shift invariance, we may replace the p_k terms in the numerator of (12) with the value $1/2$. Let $\equiv$ denote equivalence under invariance. (Note that with this notation we are referring to equivalence under invariance for the entire GRM, not for an individual entry.) We can write

$$K_{\alpha,hap}(i,j) \equiv \frac{1}{M} \sum_{k=1}^{M} \frac{(x_{ik} - 1/2)(x_{jk} - 1/2)}{[p_k(1-p_k)]^{-\alpha}}. \tag{20}$$

(Note that with this notation we are referring to equivalence under invariance for the entire GRM, not for an individual entry.) Since $x_{ik}, x_{jk} \in \{0, 1\}$,

$$\begin{aligned}
(x_{ik} - 1/2)(x_{jk} - 1/2) &= \frac{1}{4}(2x_{ik} - 1)(2x_{jk} - 1) \\
&= \frac{1}{4}(1 - 2(x_{ik} \oplus x_{jk})),
\end{aligned}$$

where $\oplus$ refers to the XOR of two binary values. Substituting into (20) and leveraging scale invariance and constant shift invariance,

$$\begin{aligned}K_{\alpha,hap}(i,j) &\equiv \frac{1}{4M}\sum_{k=1}^{M}\frac{(1-2(x_{ik}\oplus x_{jk}))}{[p_k(1-p_k)]^{-\alpha}} \\ &= \frac{1}{4M}\sum_{k=1}^{M}\frac{1}{[p_k(1-p_k)]^{-\alpha}} - \frac{1}{2M}\sum_{k=1}^{M}\frac{x_{ik}\oplus x_{jk}}{[p_k(1-p_k)]^{-\alpha}} \\ &\equiv \sum_{k=1}^{M}\frac{x_{ik}\oplus x_{jk}}{[p_k(1-p_k)]^{-\alpha}}. \end{aligned} \tag{21}$$

In the case of $\alpha = 0$, this simplifies to

$$K_{\alpha=0,hap}(i,j) \equiv \sum_{k=1}^{M} x_{ik}\oplus x_{jk}.$$

Note that this is the Hamming distance matrix between i and j. Under the infinite-sites model, mutations for which samples i and j differ occur on the area of the 2-sample ARG from samples i and j to their TMRCA along the genome. The number of such mutations is a Poisson random variable with rate μ (the per-site per-generation mutation rate) times the area (sites times generations) of the 2-sample ARG. We express the areas as $2 \times L \times \bar{t}_{ij}$, where $\bar{t}_{ij}$ is the genome-wide average TMRCA of i and j and L is the physical extent of the ARG. Then,

$$\begin{aligned}K_{\alpha=0,hap,ARG}(i,j) &= \mathbb{E}\left[K_{\alpha=0,hap}(i,j)|ARG\right] \\ &\equiv \mathbb{E}\left[\sum_{k=1}^{M} x_{ik}\oplus x_{jk}\middle| ARG\right] \\ &= \mathbb{E}\left[\text{Poisson}(2\times L\times \bar{t}_{ij})\right] \\ &= 2\times L\times \bar{t}_{ij} \\ &\equiv \bar{t}_{ij}.\end{aligned}$$

Assuming Gower centering and data centering are used, we may therefore use the average TMRCA matrix between samples to compute the $\alpha = 0$ exact ARG-GRM. (Note that in the case of $\alpha = 0$ allele frequencies are not involved, so that the ARG-GRM may be estimated using pairwise TMRCA estimates alone [38], e.g., using ASMC.)

For the case of general α, consider (21), then

$$\begin{aligned}K_{\alpha,hap,ARG}(i,j) &\equiv \mathbb{E}\left[\frac{1}{M}\sum_{k=1}^{M}\frac{x_{ik}\oplus x_{jk}}{[p_k(1-p_k)]^{-\alpha}}\middle| ARG\right] \\ &= \mathbb{E}_{m\sim Uniform(ARG)}\left[\frac{\mathbb{1}_{i\in d(m)}\oplus \mathbb{1}_{j\in d(m)}}{[p(m)(1-p(m))]^{-\alpha}}\right] \\ &= \sum_{b\in B}\left[A(b)\cdot\frac{\mathbb{1}_{i\in d(b)}\oplus \mathbb{1}_{j\in d(b)}}{[p(b)(1-p(b))]^{-\alpha}}\right]\end{aligned}$$

The expression $\mathbb{1}_{i \in d(b)} \oplus \mathbb{1}_{j \in d(b)}$ is 1 if exactly one of i and j is a descendant of branch b, and 0 otherwise. Therefore we may rewrite it as $\mathbb{1}_{|d(b) \cap \{i,j\}|=1}$, where we test whether the intersection of $d(b)$ and $\{i, j\}$ contains exactly one element. Thus:

$$\begin{aligned} K_{\alpha,hap,ARG}(i,j) &\equiv \sum_{b \in B} \left[A(b) \cdot \frac{\mathbb{1}_{|d(b) \cap \{i,j\}|=1}}{\left[p(b)\left(1 - p(b)\right)\right]^{-\alpha}} \right] \\ &= \sum_{b \in B, |d(b) \cap \{i,j\}|=1} \left[\frac{A(b)}{\left[p(b)\left(1 - p(b)\right)\right]^{-\alpha}} \right] \end{aligned} \tag{22}$$

The branches $b \in B$ with $|d(b) \cap \{i, j\}| = 1$ are those that lie in the 2-sample ARG containing samples i and j. Computing (22) is achieved by iterating over all these branches and summing their areas, weighted by a term involving $p(b)$ and α. When $\alpha = 0$, this reduces to summing the areas of all branches in the 2-sample ARG:

$$\begin{aligned} K_{\alpha=0,hap,ARG}(i,j) &\equiv \sum_{b \in B, |d(b) \cap \{i,j\}|=1} A(b) \\ &= 2 \times L \times \bar{t}_{ij}, \end{aligned}$$

which coincides with the earlier derivation for $\alpha = 0$.

The MAF-stratified version of the haploid α GRM is similarly derived, except iteration is restricted to branches b with allele frequency $p(b)$ within a specified range. This generalizes to other stratification criteria.

Exact ARG-GRM, diploid and general α

Finally, we describe the case of diploid genotypes and general α. We separate (19) into four terms:

$$\begin{aligned} K_{\alpha,dip}(i,j) &= \frac{1}{M} \sum_{k=1}^{M} \frac{\left(x_{i_1 k} + x_{i_2 k} - 2p_k\right)\left(x_{j_1 k} + x_{j_2 k} - 2p_k\right)}{\left[2p_k\left(1 - p_k\right)\right]^{-\alpha}} \\ &= \frac{1}{M} \sum_{k=1}^{M} \frac{\left[\left(x_{i_1 k} - p_k\right) + \left(x_{i_2 k} - p_k\right)\right]\left[\left(x_{j_1 k} - p_k\right) + \left(x_{j_2 k} - p_k\right)\right]}{\left[2p_k\left(1 - p_k\right)\right]^{-\alpha}} \\ &= \frac{2^\alpha}{M} \left[\sum_{k=1}^{M} \frac{\left(x_{i_1 k} - p_k\right)\left(x_{j_1 k} - p_k\right)}{\left[p_k\left(1 - p_k\right)\right]^{-\alpha}} + \sum_{k=1}^{M} \frac{\left(x_{i_1 k} - p_k\right)\left(x_{j_2 k} - p_k\right)}{\left[p_k\left(1 - p_k\right)\right]^{-\alpha}} + \right. \\ &\qquad \left. \sum_{k=1}^{M} \frac{\left(x_{i_2 k} - p_k\right)\left(x_{j_1 k} - p_k\right)}{\left[p_k\left(1 - p_k\right)\right]^{-\alpha}} + \sum_{k=1}^{M} \frac{\left(x_{i_2 k} - p_k\right)\left(x_{j_2 k} - p_k\right)}{\left[p_k\left(1 - p_k\right)\right]^{-\alpha}} \right] \\ &= 2^\alpha \left[K_{\alpha,hap}(i_1, j_1) + K_{\alpha,hap}(i_1, j_2) + K_{\alpha,hap}(i_2, j_1) + K_{\alpha,hap}(i_2, j_2) \right], \end{aligned}$$

where terms such as $K_{\alpha,hap}(i_1, j_1)$ correspond to the haploid GRM over $2N$ samples given by (12). Using scale invariance, the factor of 2^α can be removed to write

$$K_{\alpha,dip}(i,j) \equiv K_{\alpha,hap}(i_1, j_1) + K_{\alpha,hap}(i_1, j_2) + K_{\alpha,hap}(i_2, j_1) + K_{\alpha,hap}(i_2, j_2).$$

A similar expression may be obtained for the ARG-GRMs:

$$
\begin{aligned}
K_{\alpha,dip,ARG}(i,j) &= \mathbb{E}\left[K_{\alpha,dip}(i,j)|ARG\right] \\
&\equiv \mathbb{E}\left[K_{\alpha,hap}(i_1,j_1)|ARG\right] + \mathbb{E}\left[K_{\alpha,hap}(i_1,j_2)|ARG\right] + \\
&\quad\ \mathbb{E}\left[K_{\alpha,hap}(i_2,j_1)|ARG\right] + \mathbb{E}\left[K_{\alpha,hap}(i_2,j_2)|ARG\right] \\
&= K_{\alpha,hap,ARG}(i_1,j_1) + K_{\alpha,hap,ARG}(i_1,j_2) + K_{\alpha,hap,ARG}(i_2,j_1) + K_{\alpha,hap,ARG}(i_2,j_2).
\end{aligned}
$$

The diploid ARG-GRM is therefore obtained by first computing the exact ARG-GRM of size $2N$ by $2N$ over the haploid samples and then using terms involving (i_1, j_1), (i_1, j_2), (i_2, j_1), and (i_2, j_2) to compute the entry for pair (i, j).

Supplementary Note 4: Additional details on ARG-based analyses

ARG-MLMA methods

In our ARG-based mixed linear model association (ARG-MLMA) approach, we sample mutations from a given ARG using a specified rate μ and apply a mixed model association test to these variants. In the case of an inferred ARG, testing these sampled mutations may reveal putatively associated variants. The choice of μ may be used to decrease the number of tests performed, while also accounting for uncertainty of the inferred variants within the ARG. Meanwhile, the mixed model controls for population stratification and relatedness while increasing association power compared to linear regression testing of variants [39]. When testing the ARG of a particular chromosome, we include array variants from all other chromosomes as random effects in the mixed model, a design known as LOCO (leave one chromosome out) [40, 41, 39]. Instead of array variants, the ARG-MLMA approach can also be extended to use sequencing variants or ARG-GRMs for the random effects component, which we expect to further improve power (see Fig. 3c and Supplementary Fig. 8c,f, where the testing is of array variants rather than ARG variants).

For simulation experiments (Fig. 3a and Extended Data Fig. 6) we traversed the ARG and wrote out all possible mutations to disk, which is equivalent to adopting a large value of μ, and used BOLT-LMM-inf to perform linear regression and LMM testing of these mutations. We used sequencing variants from chromosomes 2-22 to form a polygenic background with narrow-sense heritability $h^2 = 0.8$ and negative selection parameter [42] $\alpha = -0.25$. In detail, we drew effects $\beta_i \sim \mathcal{N}(0, [p_i(1 - p_i)]^\alpha)$, computed $y_g = \sum_i \beta_i x_i$ using unnormalized haploid genotypes x_i, and scaled y_g to have variance h^2. We then added a single causal sequencing variant on chromosome 1 (chosen at random from those with allele frequency $p \in \{0.01, 0.005, 0.0025\}$) with effect size β and added independent normally-distributed noise for the remaining environmental variance. We varied the value of β and measured association power for each method as the fraction of runs (out of 100) detecting a significant association on chromosome 1. Significance thresholds for each method were calibrated to yield a family-wise error rate of 0.05 under the null condition $\beta = 0$. We compared between association of array data, imputed data (within-cohort imputation using IMPUTE4 [43]), the true ARG, and an ARG inferred by ARG-Needle using array data (Fig. 3a and Extended Data Fig. 6). Compared to testing with linear regression, we observed that MLMA with a random component consisting of array markers on chromosomes 2-22 improved association power (Extended Data Fig. 6d).

We also extended the above ARG-MLMA experiment to a setting with diploid individuals and phasing error (Extended Data Fig. 6e). For association with N individuals, we simulated an ARG with $2N$ haploid samples, and merged pairs to obtain N sets of diploid genotypes. To incorporate realistic switch errors, we phased these diploid genotypes with Beagle 5.1 [20] using reference-free phasing. We used ARG-Needle to infer ARGs on either the $2N$ phased samples or on the original $2N$ haploid samples where the true phase is known. Trait simulation and association used the same parameters

as above but with diploid genotypes. For comparisons to imputation, we assumed that true phase is known for both the $2N$ haploid samples and the reference samples.

For ARG-MLMA analyses in the UK Biobank we adopted $\mu = 10^{-5}$, also adding variants sampled with $\mu = 10^{-3}$ to locus-specific Manhattan plots to gain further insights in the association regions. To achieve greater scalability, we developed a pipeline to test ARG variants without writing out to disk. We first regressed out covariates from the phenotype, then ran BOLT-LMM on SNPs from all chromosomes to extract BOLT-LMM's calculated calibration factor. We additionally ran BOLT-LMM 22 times, once with each chromosome excluded, and extracted estimated prediction effects (`--predBetasFile` flag) to form LOCO polygenic predictors. We then obtained LOCO residuals by subtracting these LOCO predictions from the processed phenotype. We finally used ARG-Needle to test clades of the ARG for association against these residuals, traversing the ARG and calculating BOLT-LMM's non-infinitesimal test statistics for each clade of interest. Our methods include runtime optimizations for sparse clades, as well as options to sample clades based on MAF or a mutation rate.

Comparison to IBD-based association

We performed simulations comparing ARG-based association analysis to a related approach that relies on the sharing of identical-by-descent (IBD) haplotypes between pairs of individuals [33, 44, 45]. We used the ARGON simulator [16] to perform 100 independent simulations involving 1,000 synthetic haploid samples for a 10 Mb-long genomic region, assuming a constant population size of 30,000 individuals and a recombination rate of 1 Mb/cM. We extracted ground truth IBD data from the simulated ARG using ARGON's `-ibd` flag, as well as 1,000 randomly selected SNPs among variants with MAF > 0.05. We ran the DASH software [44] (CC mode, v1.1) using either ground truth IBD data or segments inferred from simulated SNPs using GERMLINE [46] (v1.5.3) with parameters recommended in the included DASH scripts (`-bits 32 -err_hom 1 -err_het 1`). We also used ARG-Needle to infer the ARG from SNP data, using the same parameters as in previous analyses and a 1.0 cM burn-in for ASMC. We then considered 10 evenly spaced sites along the simulated regions and used data from either ground truth or inferred IBD and marginal trees at these sites to compare between IBD-based and ARG-based analyses, as further described in Supplementary Fig. 7.

Computation of resampling-based significance thresholds

We used a resampling-based approach to establish genome-wide significance thresholds corresponding to a family-wise error rate of 0.05 (Supplementary Table 1). In detail, we ran 1,000 null simulations using random phenotypes drawn from a standard normal distribution, performed univariate linear regression against imputed or ARG data [47, 48, 49], and computed the minimum p-value. We then estimated the genome-wide significance threshold using the most significant 5% quantile of these minimum p-values. We verified that performing this analysis using either the entire genome, chromosome 1, or the first chunk of the genome produced compatible results in a limited number of settings. To reduce computational costs, we thus performed these analyses using a subset of the genome

and extrapolated to the whole genome. When an MAF cutoff was applied, significance thresholds remained compatible across several choices of sample size. We thus used $50{,}000$ haploid samples to estimate significance thresholds when MAF filtering was applied. Significance thresholds for several choices of filtering parameters are reported in Supplementary Table 1; specific thresholds used in individual analyses are detailed in Methods.

Association analysis of 7 traits

Using the filtering criteria described in Methods and no additional MAF cutoff, we performed genome-wide ARG and imputation MLMA for 7 traits, with resampling-based significance thresholds of $p < 4.8 \times 10^{-11}$ (ARG) and $p < 1.06 \times 10^{-9}$ (imputation). We then selected genomic regions harboring low-frequency ($0.1\% \leq$ MAF $< 1\%$), rare ($0.01\% \leq$ MAF $< 0.1\%$), or ultra-rare (MAF $< 0.01\%$) variant associations.

We next performed several further filtering and association analyses to extract sets of approximately independent signals, using PLINK (v1.90b6.21) and a procedure similar to that of [50]. For each region, we extracted hard-called raw genotypes for all genome-wide significant signals with MAF $< 1\%$ from either ARG or imputed data, tested for association (`--assoc` flag), and performed two-stage LD-clumping of the variants. The first clumping step used parameters `--clump-p1 0.0001 --clump-p2 0.0001 --clump-r2 0.5 --clump-kb 10`; the second used same parameters except for `--clump-kb 100000`. For each variant i, we considered each other variant j and computed the approximate chi-squared statistic that would be obtained by including j as covariate [51, 50]:

$$\chi^2_{i|j} = \chi^2_i \left(1 - \text{sign}(\beta_i\beta_j) r_{ij} \sqrt{\chi^2_j/\chi^2_i}\right)^2, \tag{23}$$

where χ^2_i and χ^2_j denote the respective chi-square statistics and sign$(\beta_i\beta_j)$ is 1 if the effect sizes for the two variants have the same sign, -1 otherwise. LD was computed using the `--r` flag. We only retained variants i such that $\chi^2_{i|j}$ remained significant for all choices of j. For these variants, we reported the conditional p-value as the maximum p-value for $\chi^2_{i|j}$ across all choices of j. We refer to the set of variants remaining after these filtering steps as approximately independent ("independent" for short, reported in Supplementary Tables 2-5).

For computing pairwise LD between the set of independent associated variants and WES variants to determine "WES partners" (see Methods), we used the `--r` flag. For each pair of independent ARG or imputation signals with their WES partners, we computed the distance to the WES partner, the LD with the WES partner, the values of association $\hat{\beta}$ (with standard error), and the confusion matrix of genotype overlap between the independent signal and the WES partner, which we used to determine precision and recall of predicting the carriers in the WES variant (reported in Supplementary Tables 2-5). Because the BOLT-LMM non-infinitesimal model does not produce $\hat{\beta}$ estimates, we instead obtained them using PLINK (`--assoc` flag).

Variant annotations for the WES partners used in Fig. 4c were obtained using the Ensembl Variant Effect Predictor (VEP) tool [52]; we refer to variants labeled by VEP as

[frameshift_variant, splice_acceptor_variant, splice_donor_variant, stop_gained] as "loss-of-function" (LoF) and to variants with other protein altering labels [stop_lost, start_lost, missense_variant, inframe_deletion, inframe_insertion] as "other protein altering". We also used the annotations for all variants in the whole exome sequencing data to compute background fractions of these two functional classes (see Fig. 4c); note that we did not restrict to only exons, as likewise our WES partners were not constrained to only exons. Gene annotations for each WES partner variant were also obtained from VEP, where we allow for each WES partner to be annotated with multiple genes if they overlap the WES variant position. We used the WES partner positions to check against the results of [50] to assess whether the variants we detected were present in the summary statistics, marginally significant ($p < 5 \times 10^{-8}$), or reported as likely causal (defined in [50]).

Association analysis for higher frequency variants with height

To detect approximately independent associations using COJO (Fig. 5e-f, Extended Data Fig. 10e, Supplementary Fig. 11) we first performed LD clumping of associated variants using PLINK (v1.90b6.21) with flags `--clump-p1 0.0001 --clump-p2 0.0001 --clump-r2 0.5 --clump-kb 1000` for all data types. For ARG data we also pre-processed each of the 749 non-overlapping chunks by running LD-clumping with the same parameters but with a reduced `--clump-kb 100`. The GCTA software implementing the COJO procedure requires effect size estimates, which are not produced by the BOLT-LMM non-infinitesimal model. We therefore extracted genotype variants with MLMA $p < 5 \times 10^{-7}$ for array data and $p < 5 \times 10^{-8}$ for imputed and ARG data and performed further association analysis using PLINK (v2.00a3LM, `--glm` flag). To create merged data sets (e.g., ARG + SNP array data), we started from the variants that were selected from the LD clumping step and merged them using PLINK. Because imputed data contains genotype array markers, we first removed any overlapping markers from the set of LD-clumped imputed variants in cases where both imputed and array data were merged and separately considered. COJO analyses were performed using GCTA (v1.93.2) using the flags `--cojo-slct --cojo-p 5e-8`, retaining results with COJO $p < 3 \times 10^{-9}$. Independent variants within merged data sets were attributed to different data types (SNP/imputation/ARG) based on unique variant identifiers.

ARG-based genotype imputation

Given a collection of sequencing and array samples, we use ARG-Needle in sequencing mode to first thread the sequencing samples, then thread the array samples using array mode, using a 1.0 cM ASMC burn-in and otherwise standard parameters. For each site we wish to impute, we consider the corresponding marginal tree in the inferred ARG, and select the branches for which an unseen mutation best explains the observed sequencing data in terms of Hamming distance (see Extended Data Fig. 5b). Each of these branches implies genotypes of 0 or 1 for the array samples. We output a weighted average of the implied genotypes, weighting branches by their length in the marginal tree. We applied this framework to perform genotype imputation using ground-truth ARGs and ARG-Needle inferred ARGs

in 10 Mb of simulated data and compared to IMPUTE4 [43] imputation using the binned aggregate r^2 metric [53] (Supplementary Fig. 12).

References (Supplementary Information)

[1] Robert C Griffiths and Paul Marjoram. An ancestral recombination graph. *Institute for Mathematics and its Applications*, 87:257, 1997.

[2] John Frank Charles Kingman. The coalescent. *Stochastic Processes and their Applications*, 13(3):235–248, 1982.

[3] Richard R Hudson. Properties of a neutral allele model with intragenic recombination. *Theoretical Population Biology*, 23(2):183–201, 1983.

[4] Richard R Hudson et al. Gene genealogies and the coalescent process. *Oxford Surveys in Evolutionary Biology*, 7(1):44, 1990.

[5] Jotun Hein, Mikkel Schierup, and Carsten Wiuf. *Gene genealogies, variation and evolution: a primer in coalescent theory*. Oxford University Press, 2004.

[6] Carsten Wiuf and Jotun Hein. The ancestry of a sample of sequences subject to recombination. *Genetics*, 151(3):1217–1228, 1999.

[7] Carsten Wiuf and Jotun Hein. Recombination as a point process along sequences. *Theoretical Population Biology*, 55(3):248–259, 1999.

[8] Gilean AT McVean and Niall J Cardin. Approximating the coalescent with recombination. *Philosophical Transactions of the Royal Society B: Biological Sciences*, 360(1459):1387–1393, 2005.

[9] Paul Marjoram and Jeff D Wall. Fast "coalescent" simulation. *BMC Genetics*, 7(1):1–9, 2006.

[10] Gary K Chen, Paul Marjoram, and Jeffrey D Wall. Fast and flexible simulation of DNA sequence data. *Genome Research*, 19(1):136–142, 2009.

[11] Laurent Excoffier and Matthieu Foll. Fastsimcoal: a continuous-time coalescent simulator of genomic diversity under arbitrarily complex evolutionary scenarios. *Bioinformatics*, 27(9):1332–1334, 2011.

[12] Paul R Staab, Sha Zhu, Dirk Metzler, and Gerton Lunter. scrm: Efficiently simulating long sequences using the approximated coalescent with recombination. *Bioinformatics*, 31(10):1680–1682, 2015.

[13] Jerome Kelleher, Alison M Etheridge, and Gilean McVean. Efficient coalescent simulation and genealogical analysis for large sample sizes. *PLoS Computational Biology*, 12(5):e1004842, 2016.

[14] Jerome Kelleher, Yan Wong, Anthony W Wohns, Chaimaa Fadil, Patrick K Albers, and Gil McVean. Inferring whole-genome histories in large population datasets. *Nature Genetics*, 51(9):1330–1338, 2019.

[15] Leo Speidel, Marie Forest, Sinan Shi, and Simon R Myers. A method for genome-wide genealogy estimation for thousands of samples. *Nature Genetics*, 51(9):1321–1329, 2019.

[16] Pier Francesco Palamara. ARGON: fast, whole-genome simulation of the discrete time Wright-Fisher process. *Bioinformatics*, 32(19):3032–3034, 2016.

[17] Pier Francesco Palamara, Jonathan Terhorst, Yun S Song, and Alkes L Price. High-throughput inference of pairwise coalescence times identifies signals of selection and enriched disease heritability. *Nature Genetics*, 50(9):1311–1317, 2018.

[18] Na Li and Matthew Stephens. Modeling linkage disequilibrium and identifying recombination hotspots using single-nucleotide polymorphism data. *Genetics*, 165(4):2213–2233, 2003.

[19] Richard Durbin. Efficient haplotype matching and storage using the positional Burrows–Wheeler transform (PBWT). *Bioinformatics*, 30(9):1266–1272, 2014.

[20] Brian L Browning, Ying Zhou, and Sharon R Browning. A one-penny imputed genome from next-generation reference panels. *The American Journal of Human Genetics*, 103(3):338–348, 2018.

[21] Olivier Delaneau, Jean-François Zagury, Matthew R Robinson, Jonathan L Marchini, and Emmanouil T Dermitzakis. Accurate, scalable and integrative haplotype estimation. *Nature Communications*, 10(1):1–10, 2019.

[22] Simone Rubinacci, Olivier Delaneau, and Jonathan Marchini. Genotype imputation using the Positional Burrows Wheeler Transform. *PLoS Genetics*, 16(11):e1009049, 2020.

[23] Po-Ru Loh, Petr Danecek, Pier Francesco Palamara, Christian Fuchsberger, Yakir A Reshef, Hilary K Finucane, Sebastian Schoenherr, Lukas Forer, Shane McCarthy, Goncalo R Abecasis, et al. Reference-based phasing using the Haplotype Reference Consortium panel. *Nature Genetics*, 48(11):1443–1448, 2016.

[24] Juba Nait Saada, Georgios Kalantzis, Derek Shyr, Fergus Cooper, Martin Robinson, Alexander Gusev, and Pier Francesco Palamara. Identity-by-descent detection across 487,409 British samples reveals fine scale population structure and ultra-rare variant associations. *Nature Communications*, 11(1):1–15, 2020.

[25] Matthew D Rasmussen, Melissa J Hubisz, Ilan Gronau, and Adam Siepel. Genome-wide inference of ancestral recombination graphs. *PLoS Genetics*, 10(5):e1004342, 2014.

[26] Sebastian Böcker and Andreas WM Dress. Recovering symbolically dated, rooted trees from symbolic ultrametrics. *Advances in Mathematics*, 138(1):105–125, 1998.

[27] David F Robinson and Leslie R Foulds. Comparison of weighted labelled trees. In *Combinatorial Mathematics VI*, pages 119–126. Springer, 1979.

[28] Mary K Kuhner and Joseph Felsenstein. A simulation comparison of phylogeny algorithms under equal and unequal evolutionary rates. *Molecular Biology and Evolution*, 11(3):459–468, 1994.

[29] Michelle Kendall and Caroline Colijn. Mapping phylogenetic trees to reveal distinct patterns of evolution. *Molecular Biology and Evolution*, 33(10):2735–2743, 2016.

[30] MR Smith. Robust analysis of phylogenetic tree space. *Systematic Biology*, 2021.

[31] Joel Mefford, Danny Park, Zhili Zheng, Arthur Ko, Mika Ala-Korpela, Markku Laakso, Päivi Pajukanta, Jian Yang, John Witte, and Noah Zaitlen. Efficient estimation and applications of cross-validated genetic predictions to polygenic risk scores and linear mixed models. *Journal of Computational Biology*, 27(4):599–612, 2020.

[32] Jian Yang, S Hong Lee, Michael E Goddard, and Peter M Visscher. GCTA: a tool for genome-wide complex trait analysis. *The American Journal of Human Genetics*, 88(1):76–82, 2011.

[33] Shaun Purcell, Benjamin Neale, Kathe Todd-Brown, Lori Thomas, Manuel AR Ferreira, David Bender, Julian Maller, Pamela Sklar, Paul IW De Bakker, Mark J Daly, et al. PLINK: a tool set for whole-genome association and population-based linkage analyses. *The American Journal of Human Genetics*, 81(3):559–575, 2007.

[34] Po-Ru Loh, George Tucker, Brendan K Bulik-Sullivan, Bjarni J Vilhjalmsson, Hilary K Finucane, Rany M Salem, Daniel I Chasman, Paul M Ridker, Benjamin M Neale, Bonnie Berger, et al. Efficient Bayesian mixed-model analysis increases association power in large cohorts. *Nature Genetics*, 47(3):284–290, 2015.

[35] Po-Ru Loh, Gleb Kichaev, Steven Gazal, Armin P Schoech, and Alkes L Price. Mixed-model association for biobank-scale datasets. *Nature Genetics*, 50(7):906–908, 2018.

[36] Po-Ru Loh, Gaurav Bhatia, Alexander Gusev, Hilary K Finucane, Brendan K Bulik-Sullivan, Samuela J Pollack, Teresa R de Candia, Sang Hong Lee, Naomi R Wray, Kenneth S Kendler, et al. Contrasting genetic architectures of schizophrenia and other complex diseases using fast variance-components analysis. *Nature Genetics*, 47(12):1385, 2015.

[37] Andy Dahl, Khiem Nguyen, Na Cai, Michael J Gandal, Jonathan Flint, and Noah Zaitlen. A robust method uncovers significant context-specific heritability in diverse complex traits. *The American Journal of Human Genetics*, 106(1):71–91, 2020.

[38] Pier Francesco Palamara, Jonathan Terhorst, Yun Song, and Alkes Price. Leveraging deep genealogical structure to estimate the phenotypic contribution of rare variants. Presented at the 66th Annual Meeting of The American Society of Human Genetics, Vancouver, 2016. Abstract 1931T.

[39] Jian Yang, Noah A Zaitlen, Michael E Goddard, Peter M Visscher, and Alkes L Price. Advantages and pitfalls in the application of mixed-model association methods. *Nature Genetics*, 46(2):100–106, 2014.

[40] Christoph Lippert, Jennifer Listgarten, Ying Liu, Carl M Kadie, Robert I Davidson, and David Heckerman. FaST linear mixed models for genome-wide association studies. *Nature Methods*, 8(10):833–835, 2011.

[41] Jennifer Listgarten, Christoph Lippert, Carl M Kadie, Robert I Davidson, Eleazar Eskin, and David Heckerman. Improved linear mixed models for genome-wide association studies. *Nature Methods*, 9(6):525–526, 2012.

[42] Doug Speed, Gibran Hemani, Michael R Johnson, and David J Balding. Improved heritability estimation from genome-wide SNPs. *The American Journal of Human Genetics*, 91(6):1011–1021, 2012.

[43] Clare Bycroft, Colin Freeman, Desislava Petkova, Gavin Band, Lloyd T Elliott, Kevin Sharp, Allan Motyer, Damjan Vukcevic, Olivier Delaneau, Jared O'Connell, et al. The UK Biobank resource with deep phenotyping and genomic data. *Nature*, 562(7726):203–209, 2018.

[44] Alexander Gusev, Eimear E Kenny, Jennifer K Lowe, Jaqueline Salit, Richa Saxena, Sekar Kathiresan, David M Altshuler, Jeffrey M Friedman, Jan L Breslow, and Itsik Pe'er. DASH: a method for identical-by-descent haplotype mapping uncovers association with recent variation. *The American Journal of Human Genetics*, 88(6):706–717, 2011.

[45] Sharon R Browning and Elizabeth A Thompson. Detecting rare variant associations by identity-by-descent mapping in case-control studies. *Genetics*, 190(4):1521–1531, 2012.

[46] Alexander Gusev, Jennifer K Lowe, Markus Stoffel, Mark J Daly, David Altshuler, Jan L Breslow, Jeffrey M Friedman, and Itsik Pe'er. Whole population, genome-wide mapping of hidden relatedness. *Genome Research*, 19(2):318–326, 2009.

[47] Gary A Churchill and Rebecca W Doerge. Empirical threshold values for quantitative trait mapping. *Genetics*, 138(3):963–971, 1994.

[48] Mark J Minichiello and Richard Durbin. Mapping trait loci by use of inferred ancestral recombination graphs. *The American Journal of Human Genetics*, 79(5):910–922, 2006.

[49] Masahiro Kanai, Toshihiro Tanaka, and Yukinori Okada. Empirical estimation of genome-wide significance thresholds based on the 1000 Genomes Project data set. *Journal of Human Genetics*, 61(10):861–866, 2016.

[50] Alison R Barton, Maxwell A Sherman, Ronen E Mukamel, and Po-Ru Loh. Whole-exome imputation within UK Biobank powers rare coding variant association and fine-mapping analyses. *Nature Genetics*, 53(8):1260–1269, 2021.

[51] Jian Yang, Teresa Ferreira, Andrew P Morris, Sarah E Medland, Pamela AF Madden, Andrew C Heath, Nicholas G Martin, Grant W Montgomery, Michael N Weedon, Ruth J Loos, et al. Conditional and joint multiple-SNP analysis of GWAS summary statistics identifies additional variants influencing complex traits. *Nature Genetics*, 44(4):369–375, 2012.

[52] William McLaren, Laurent Gil, Sarah E Hunt, Harpreet Singh Riat, Graham RS Ritchie, Anja Thormann, Paul Flicek, and Fiona Cunningham. The Ensembl Variant Effect Predictor. *Genome Biology*, 17(1):1–14, 2016.

[53] Shane McCarthy, Sayantan Das, Warren Kretzschmar, Olivier Delaneau, Andrew R Wood, Alexander Teumer, Hyun Min Kang, Christian Fuchsberger, Petr Danecek, Kevin Sharp, et al. A reference panel of 64,976 haplotypes for genotype imputation. *Nature Genetics*, 48(10):1279–1283, 2016.

Supplementary Figures

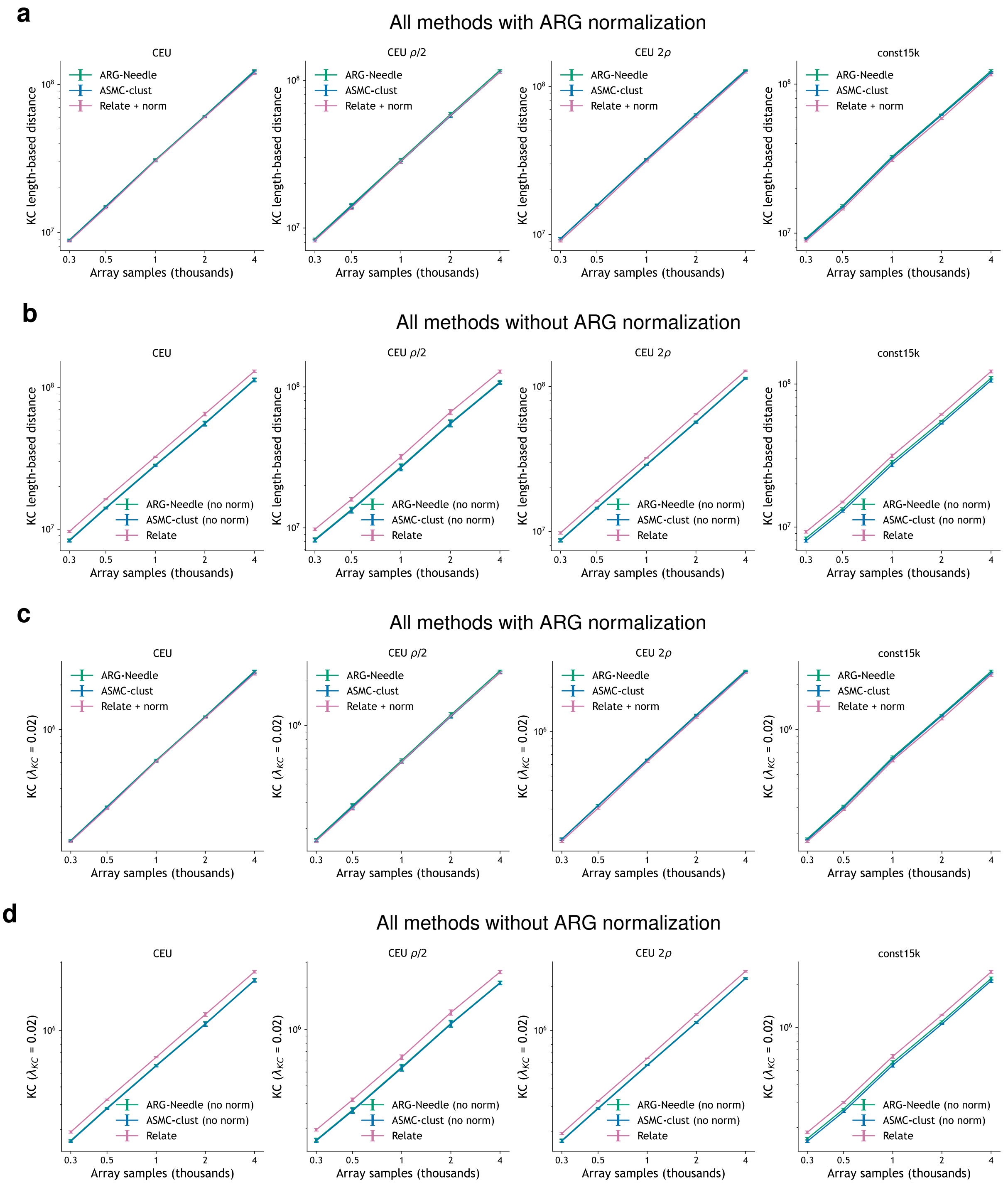

Supplementary Fig. 1: Additional comparison of ARG inference methods with array data and branch length-aware KC metrics. We compare methods as in Fig. 2, but with additional simulation conditions and branch length-aware KC metrics (see Methods). All columns are for 5 Mb of CEU demography array data, and individual columns represent standard parameters (see Methods), a factor of 2 smaller recombination rate ($\rho = 6 \times 10^{-9}$), a factor of 2 larger recombination rate ($\rho = 2.4 \times 10^{-8}$), and a constant population size demography of 15,000 individuals. We show results for the KC metric with $\lambda = 1$ (**a**-**b**) and $\lambda = 0.02$ (**c**-**d**), with (**a**,**c**) and without (**b**,**d**) ARG normalization, as these metrics are sensitive to branch length. All panels use 5 random seeds. Data are presented as means $\pm$ 2 s.e.

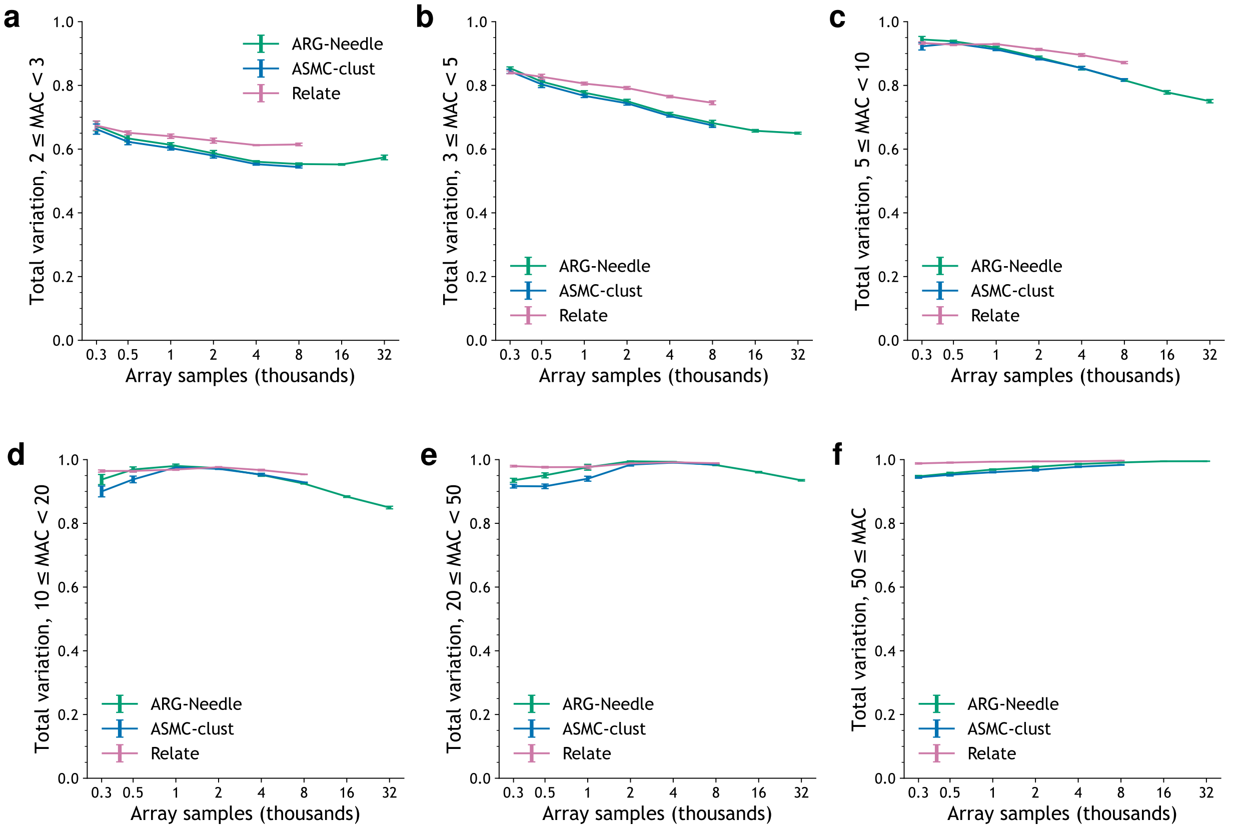

Supplementary Fig. 2: Additional comparison of ARG inference methods with array data and ARG total variation distance stratified by minor allele count (MAC). We compare methods as in Fig. 2b, stratifying the ARG total variation distance over MAC ranges (see Methods and Supplementary Note 2). All panels are for 5 Mb of CEU demography array data simulated with standard parameters (see Methods) and use 5 random seeds. **a**. $2 \leq \text{MAC} < 3$. **b**. $3 \leq \text{MAC} < 5$. **c**. $5 \leq \text{MAC} < 10$. **d**. $10 \leq \text{MAC} < 20$. **e**. $20 \leq \text{MAC} < 50$. **f**. $50 \leq \text{MAC}$. ARG-Needle and ASMC-clust include ARG normalization and Relate does not include ARG normalization, as is the default. Data are presented as means $\pm$ 2 s.e.

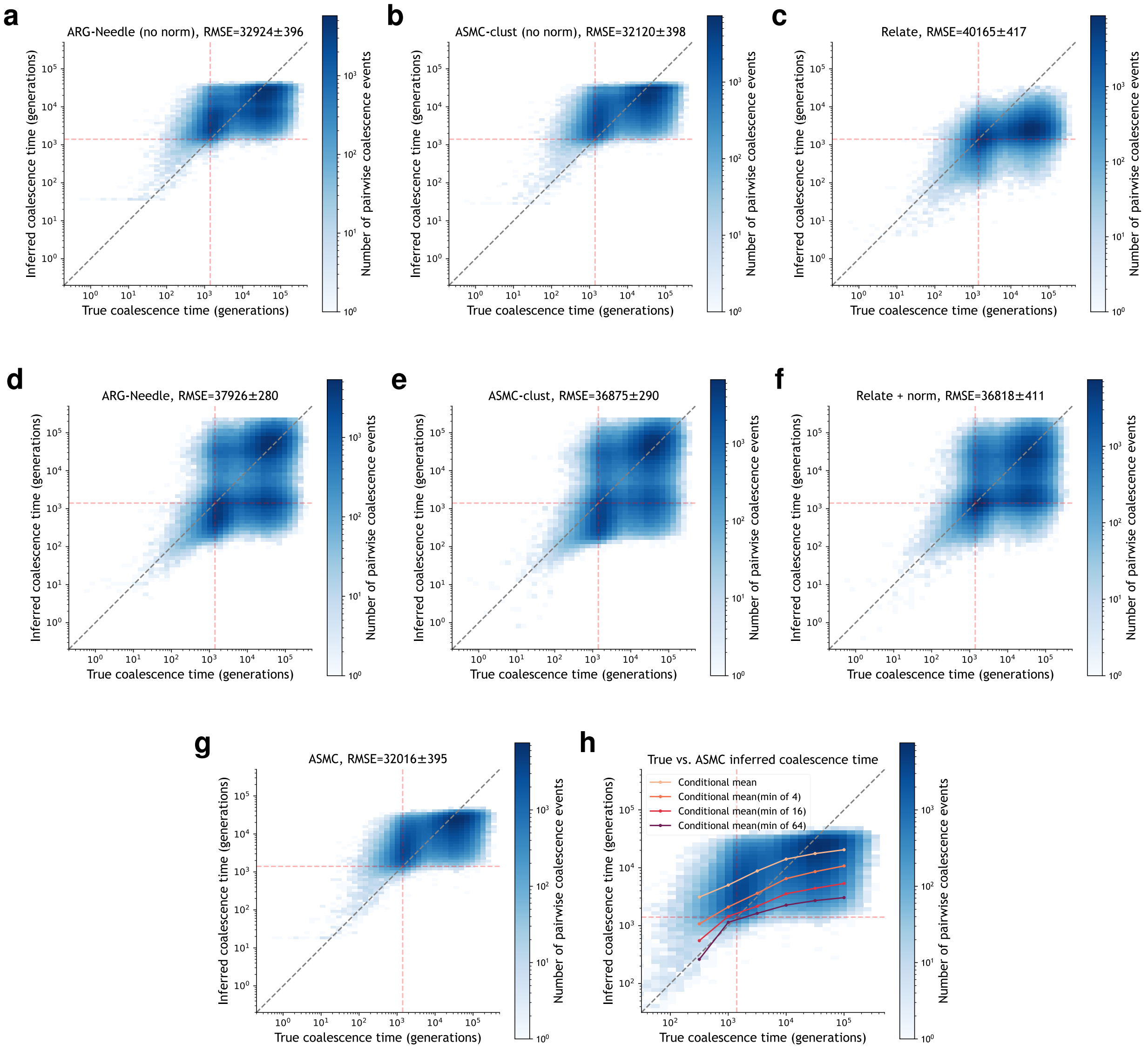

Supplementary Fig. 3: Scatter plots of true versus inferred pairwise TMRCAs. a-g. We show scatter plots for ARG-Needle (**a**,**d**), ASMC-clust (**b**,**e**), Relate (**c**,**f**), and ASMC posterior mean (**g**), for $N = 4{,}000$ array samples, showing an aggregate over 20,000 randomly sampled pairs for each of 25 simulations. The first row (**a**-**c**) corresponds to no ARG normalization and the second row (**d**-**f**) corresponds to including ARG normalization. Removing ARG normalization decreases the pairwise TMRCA RMSE for ARG-Needle and ASMC-clust, but skews the distribution of pairwise TMRCAs towards the center. Dotted red lines show the time of the CEU demography population bottleneck, when many pairs of lineages are expected to coalesce. Titles display TMRCA RMSE with 2 s.e. from bootstrap sampling of the 25 simulations used. **h**. Zoomed in view of **g**, additionally showing the conditional mean of values and conditional mean of the minimum over 4, 16, and 256 sampled values. The conditional distribution is defined by taking all data points with true pairwise TMRCA within 5% of a central value and comparing the inferred pairwise TMRCA.

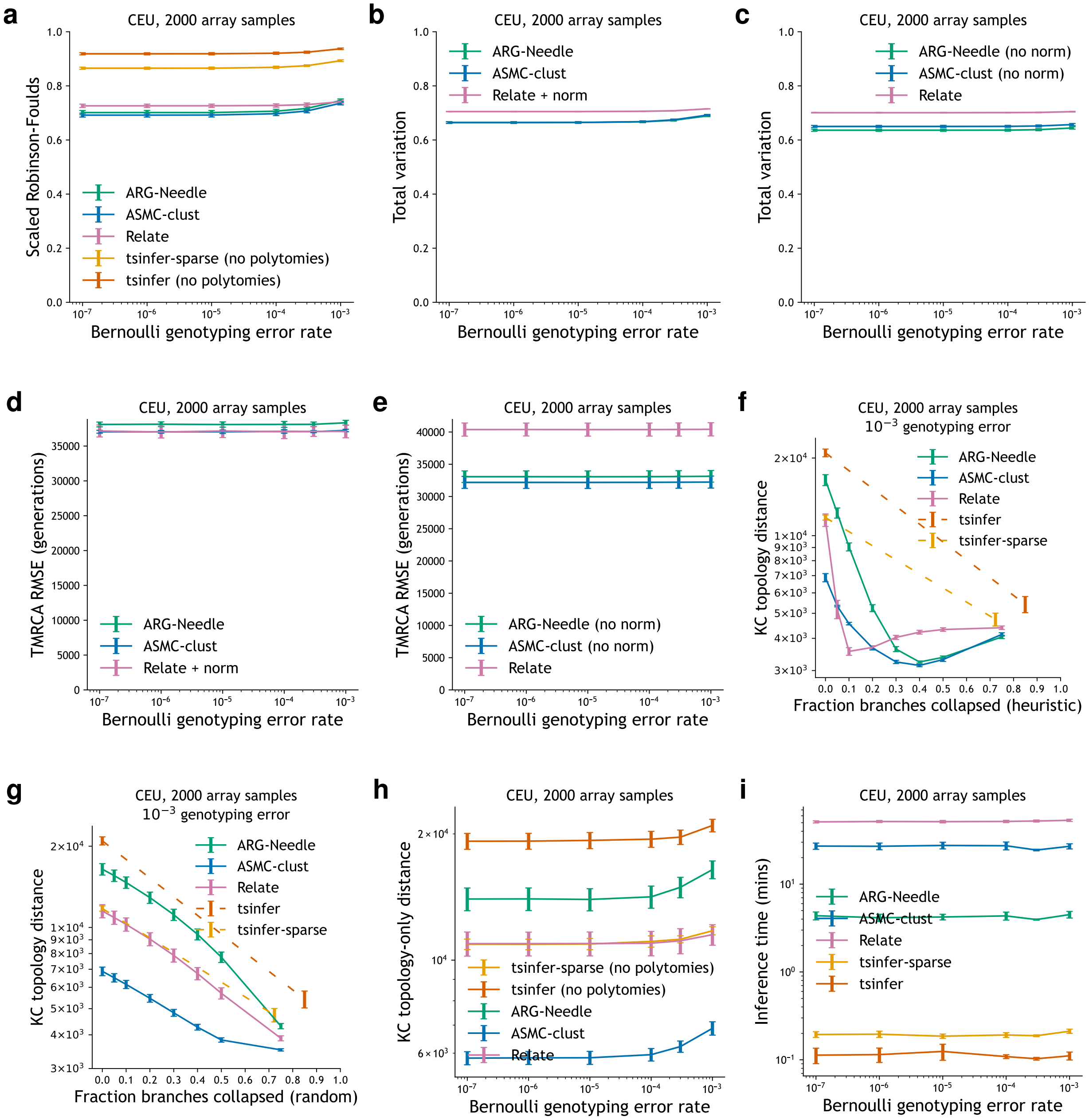

Supplementary Fig. 4: Comparison of ARG inference methods for array data with genotyping error. Simulations fix $N = 2{,}000$ haploid array samples, vary the genotyping error rate, and use otherwise standard parameters (see Methods). Individual panels correspond to rows of Extended Data Figs. 1a, 2a-d, and 1b-e, in that order, with the same metrics used, namely **a**. scaled Robinson-Foulds distance (polytomies are randomly resolved), **b-c**. ARG total variation distance with (**b**) and without (**c**) ARG normalization, **d-e**. pairwise TMRCA RMSE with (**d**) and without (**e**) ARG normalization, **f-g**. KC topology-only distance with heuristic (**f**) and random (**g**) collapsing of branches, **h**. KC topology-only distance with polytomies randomly resolved, and **i**. inference time. Because the KC merging experiments contain an additional parameter f of fraction of branches collapsed, we chose to display these results with the genotyping error rate fixed at 10^{-3} (**f-g**). All panels use 5 random seeds. Data are presented as means $\pm$ 2 s.e.

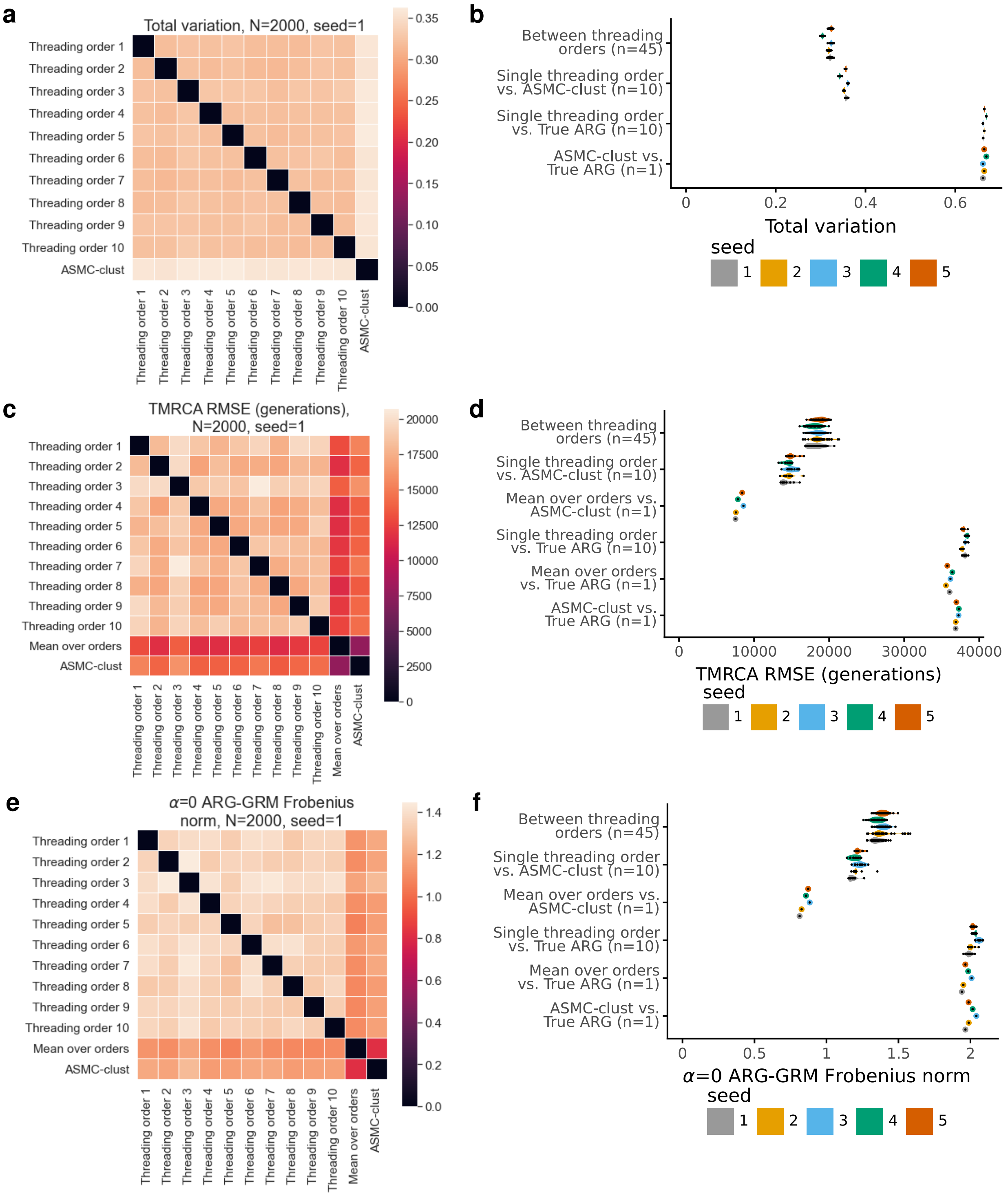

Supplementary Fig. 5: Effect of different threading orders on ARG-Needle algorithm. **a**. For a single simulation (random seed = 1) of array data for $N = 2000$ CEU demography samples, we infer 10 ARGs using ARG-Needle by processing the samples in 10 independent random threading orders. We compare the inferred ARGs against each other and against an ASMC-clust inferred ARG using the ARG total variation distance. **b**. We repeat the process for 5 total simulations (seeds) and additionally compare against the true ARG, grouping comparisons by type and seed and displaying the data distribution as violin plots with individual points overlaid. **c**-**d**. Like **a**-**b**, except using TMRCA RMSE to compare, and adding a condition where we predict pairwise TMRCAs to be the mean across the 10 threading orders. **e**-**f**. Like **a**-**b**, except using the Frobenius norm between the $\alpha = 0$ ARG-GRMs to compare (see Methods and Extended Data Fig. 7), and adding a condition where we predict a GRM by taking the mean across the 10 threading orders.

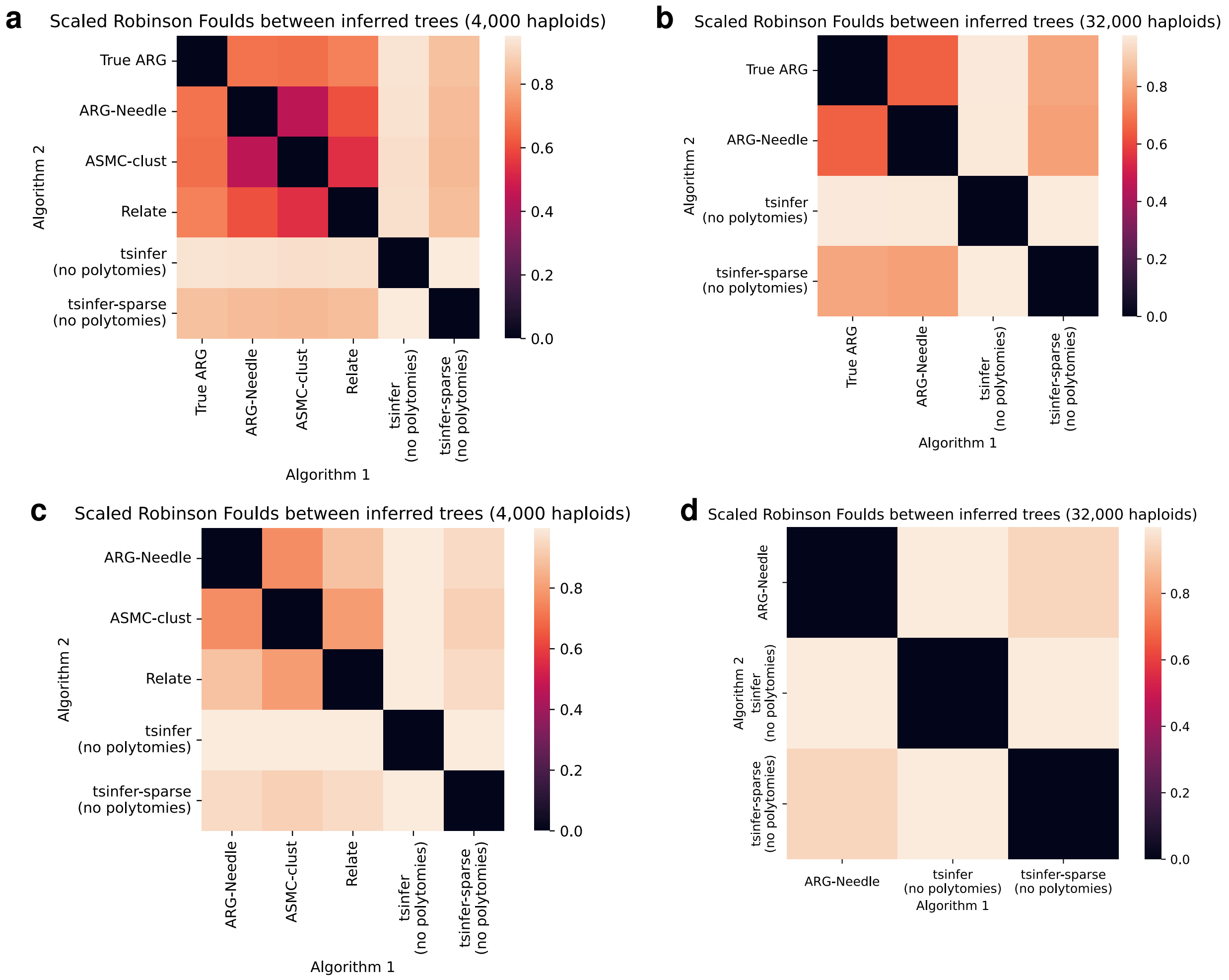

Supplementary Fig. 6: Comparison of inferred ARGs in simulated and real data using Robinson-Foulds metric. **a**. We compute the scaled Robinson-Foulds distance between the true ARG in simulations and ARGs inferred by ARG-Needle, ASMC-clust, Relate, tsinfer, and tsinfer-sparse ($N = 4{,}000$ haploid samples, 5 Mb of CEU array data, and otherwise standard parameters, see Methods). Each cell shows the mean value across 5 independent simulations. **b**. Same as **a**, but for $N = 32{,}000$ haploid samples and omitting Relate and ASMC-clust, which are less scalable. **c**. We infer ARGs using ARG-Needle, ASMC-clust, Relate, tsinfer, and tsinfer-sparse in a real data region consisting of 7.5 Mb of phased UK Biobank chromosome 1 array variants (see Methods). We subsample the data 5 times independently to create datasets of 2,000 diploid individuals (4,000 haploids). Each square shows the mean across the 5 subsamples of computing the scaled Robinson-Foulds distance between inferred ARGs. **d**. Same as **c**, except using subsampled datasets of 16,000 diploid individuals (32,000 haploids), and omitting Relate and ASMC-clust, which are less scalable. In all panels, *no polytomies* refers to randomly resolving polytomies of tsinfer and tsinfer-sparse (see Methods).

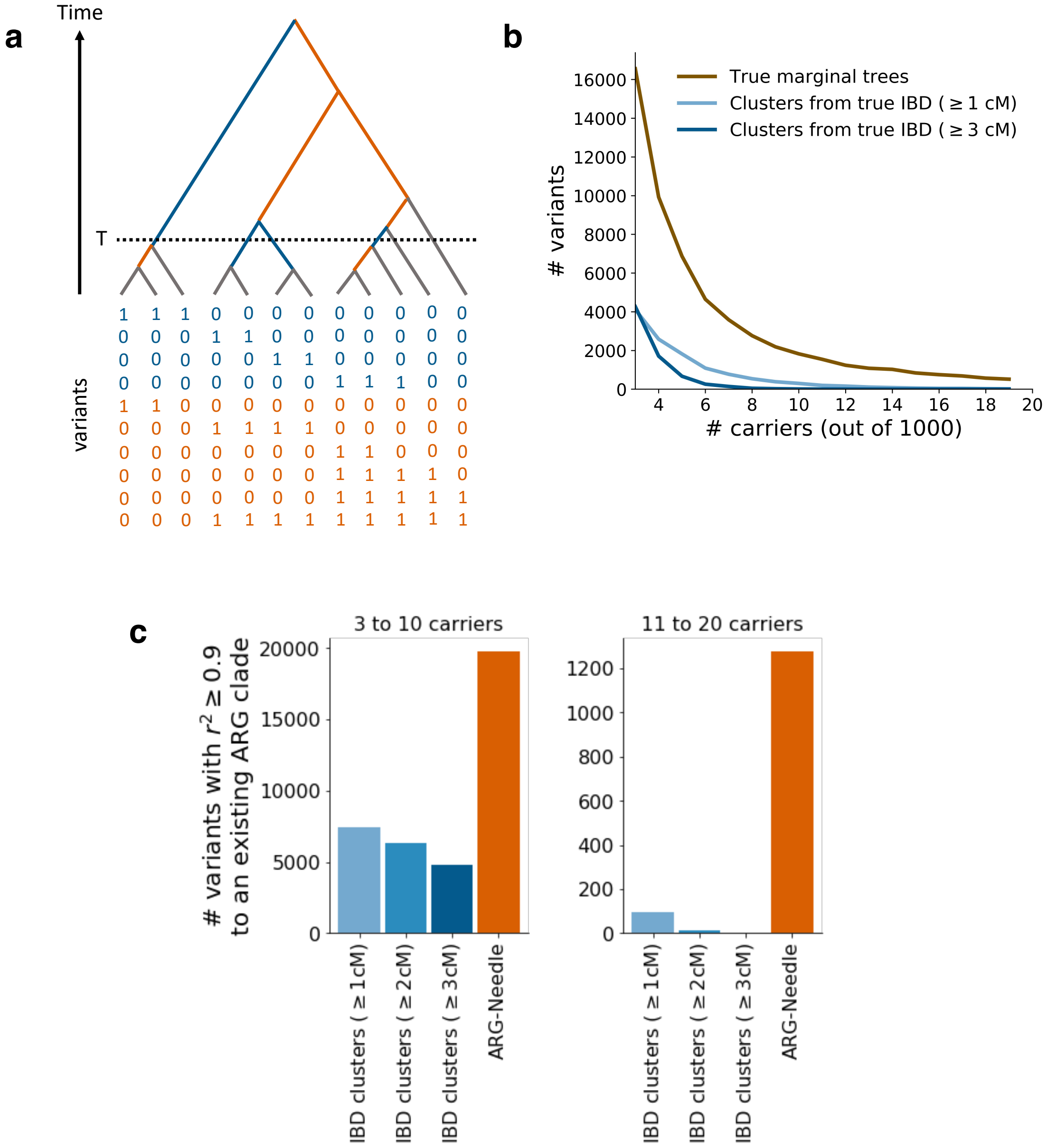

Supplementary Fig. 7: Comparison of IBD-based and ARG-based association analysis. **a**. Idealized IBD-based analysis example. Given a time threshold T, individuals are defined as identical-by-descent (IBD) at a site if they coalesce before T. In the example, individuals subtended by each blue (non-singleton) branch are IBD. The set of possible variants that may occur on these branches (hereafter "variants") is shown in blue and may be tested for association. Non-singleton variants that may exist on this marginal tree but are not implied by the threshold T and cannot be tested are shown in red. Note that in lack of TMRCA estimates, the length of IBD segments is often used as a proxy for their age and individuals sharing segments longer than a given threshold are considered IBD. **b**. Spectrum of variants (IBD clusters) inferred by the DASH algorithm using ground truth IBD data simulated for $1{,}000$ haploid samples using the ARGON simulator (see Methods). We used a length-based definition of IBD, for different length thresholds, and compared the number of inferred variants to the number of true underlying clades present in marginal trees at 10 evenly spaced sites along each region. IBD clusters are a subset of the clades in the marginal trees. **c**. For the same simulations, we extracted $1{,}000$ SNPs with MAF > 0.05. We used these phased SNPs to infer IBD using GERMLINE and to infer the ARG using ARG-Needle. We examined 10 evenly spaced sites along each simulated region and compared the number of inferred IBD clusters and ARG clades ("variants") that have high correlation ($r^2 > 0.9$) to clades in the true underlying marginal trees, focusing on variants carried by 3-10 (left) or 11-20 (right) samples.

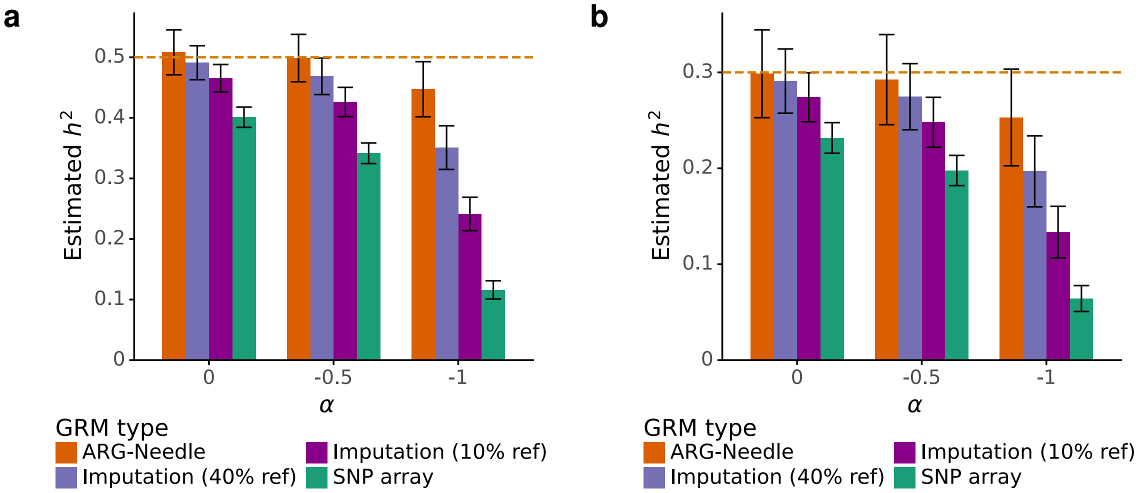

Supplementary Fig. 8: Heritability estimation using ARG-Needle and ARG-GRMs. Heritability estimation using ARG-GRMs with ARGs inferred from SNP data, compared to using GRMs of imputed data or array SNPs. As in Fig. 3b but with $h^2 = 0.5$ (**a**) and $h^2 = 0.3$ (**b**). Results are for 5 independent simulations with $N = 5{,}000$ haploid samples, 25 Mb, and $\alpha \in \{0, -0.5, -1\}$. Data are presented as estimates $\pm$ 2 s.e. from meta-analysis. *% ref* indicates the size of the reference panel used for imputation as a percentage of the number of haploid samples $N = 5{,}000$.

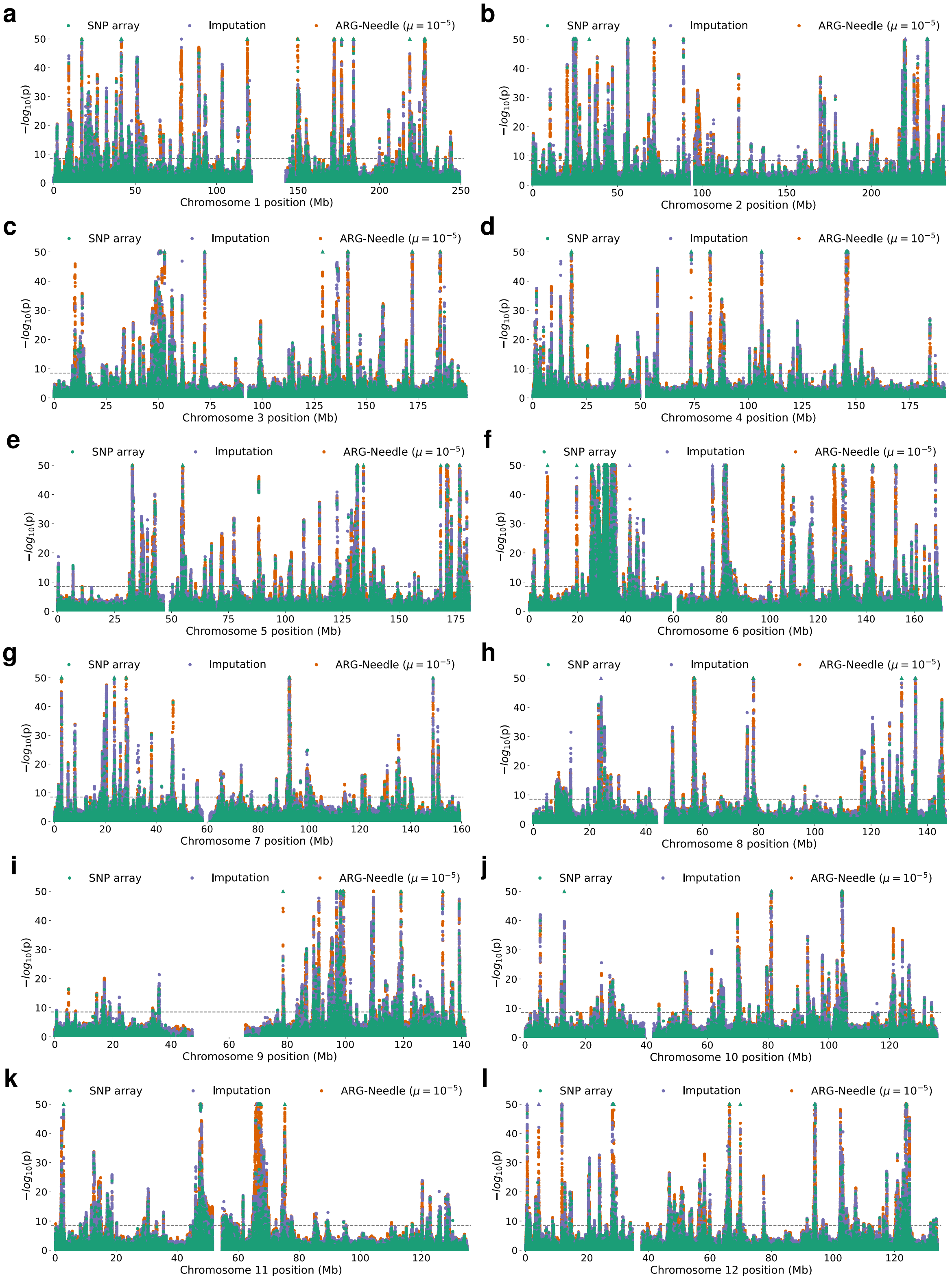
a
SNP array
Imputation
ARG-Needle ($\mu = 10^{-5}$)
$-log_{10}(p)$
Chromosome 1 position (Mb)
b
Chromosome 2 position (Mb)
c
Chromosome 3 position (Mb)
d
Chromosome 4 position (Mb)
e
Chromosome 5 position (Mb)
f
Chromosome 6 position (Mb)
g
Chromosome 7 position (Mb)
h
Chromosome 8 position (Mb)
i
Chromosome 9 position (Mb)
j
Chromosome 10 position (Mb)
k
Chromosome 11 position (Mb)
l
Chromosome 12 position (Mb)

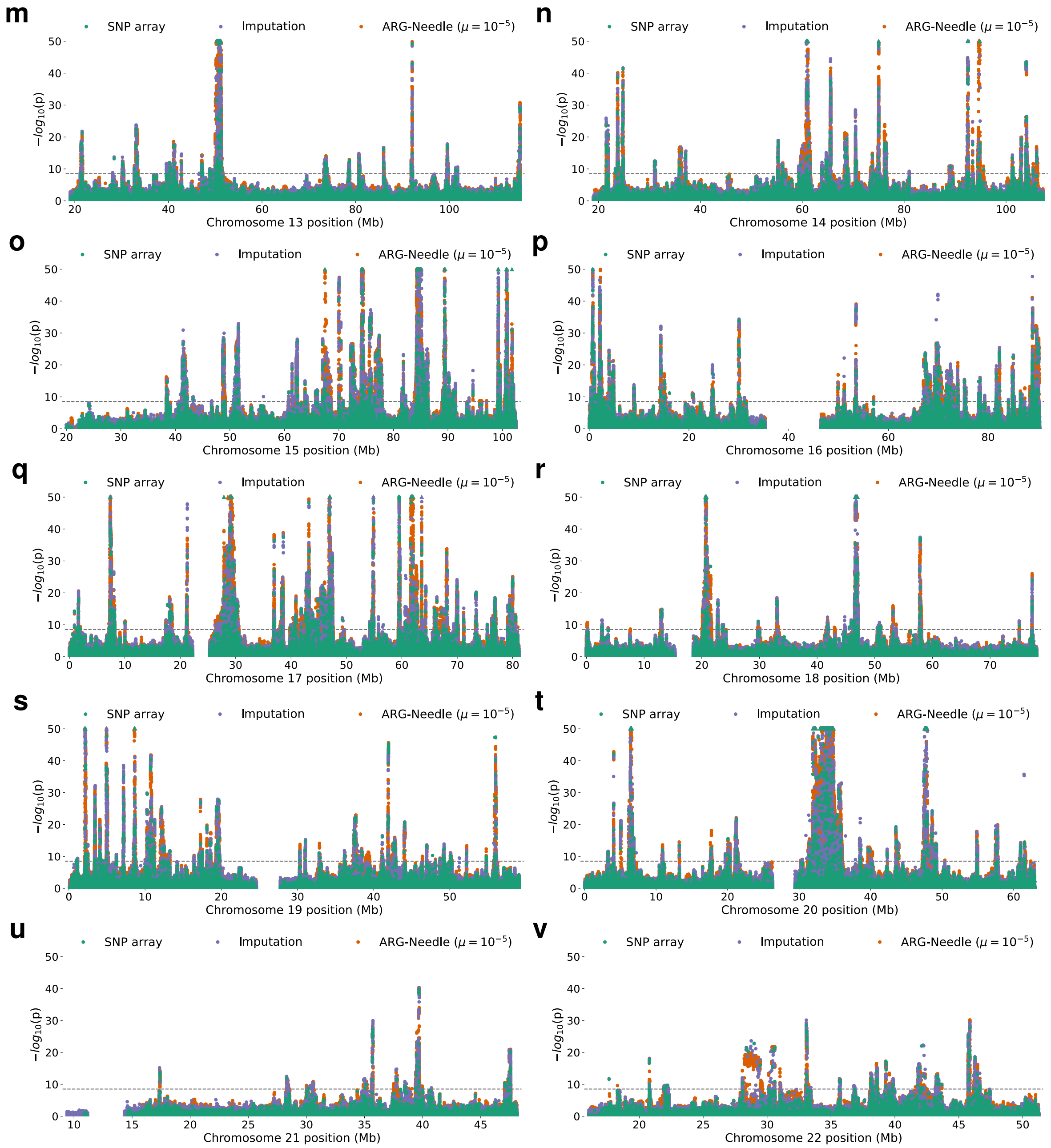

Supplementary Fig. 9: (Continued from previous page.) **Additional chromosome-wide Manhattan plots of mixed-model association of higher frequency variants with height.** Manhattan plots showing ARG-Needle, HRC+UK10K imputed variants, and SNP array association, as in Fig. 5a-b but with all methods on one plot and for all 22 chromosomes. Dotted lines correspond to $p = 3 \times 10^{-9}$ (see Methods). Triangles indicate associations with $p < 10^{-50}$. The order of plotting is ARG-Needle with $\mu = 10^{-5}$, then imputation, then SNP array variants on top.

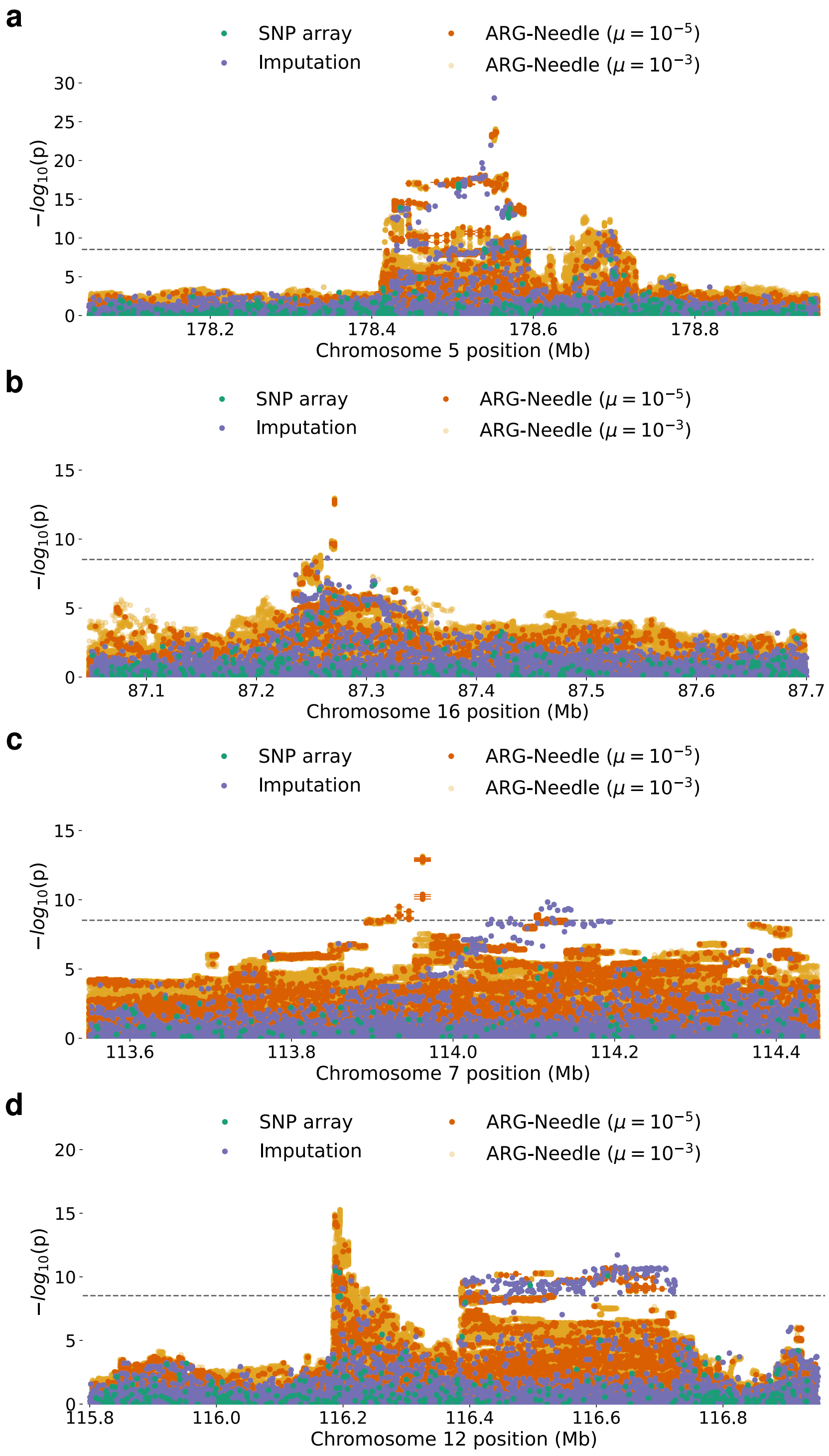
a
SNP array
Imputation
ARG-Needle ($\mu = 10^{-5}$)
ARG-Needle ($\mu = 10^{-3}$)
$-log_{10}(p)$
0
5
10
15
20
25
30
178.2
178.4
178.6
178.8
Chromosome 5 position (Mb)
b
SNP array
Imputation
ARG-Needle ($\mu = 10^{-5}$)
ARG-Needle ($\mu = 10^{-3}$)
$-log_{10}(p)$
0
5
10
15
87.1
87.2
87.3
87.4
87.5
87.6
87.7
Chromosome 16 position (Mb)
c
SNP array
Imputation
ARG-Needle ($\mu = 10^{-5}$)
ARG-Needle ($\mu = 10^{-3}$)
$-log_{10}(p)$
0
5
10
15
113.6
113.8
114.0
114.2
114.4
Chromosome 7 position (Mb)
d
SNP array
Imputation
ARG-Needle ($\mu = 10^{-5}$)
ARG-Needle ($\mu = 10^{-3}$)
$-log_{10}(p)$
0
5
10
15
20
115.8
116.0
116.2
116.4
116.6
116.8
Chromosome 12 position (Mb)

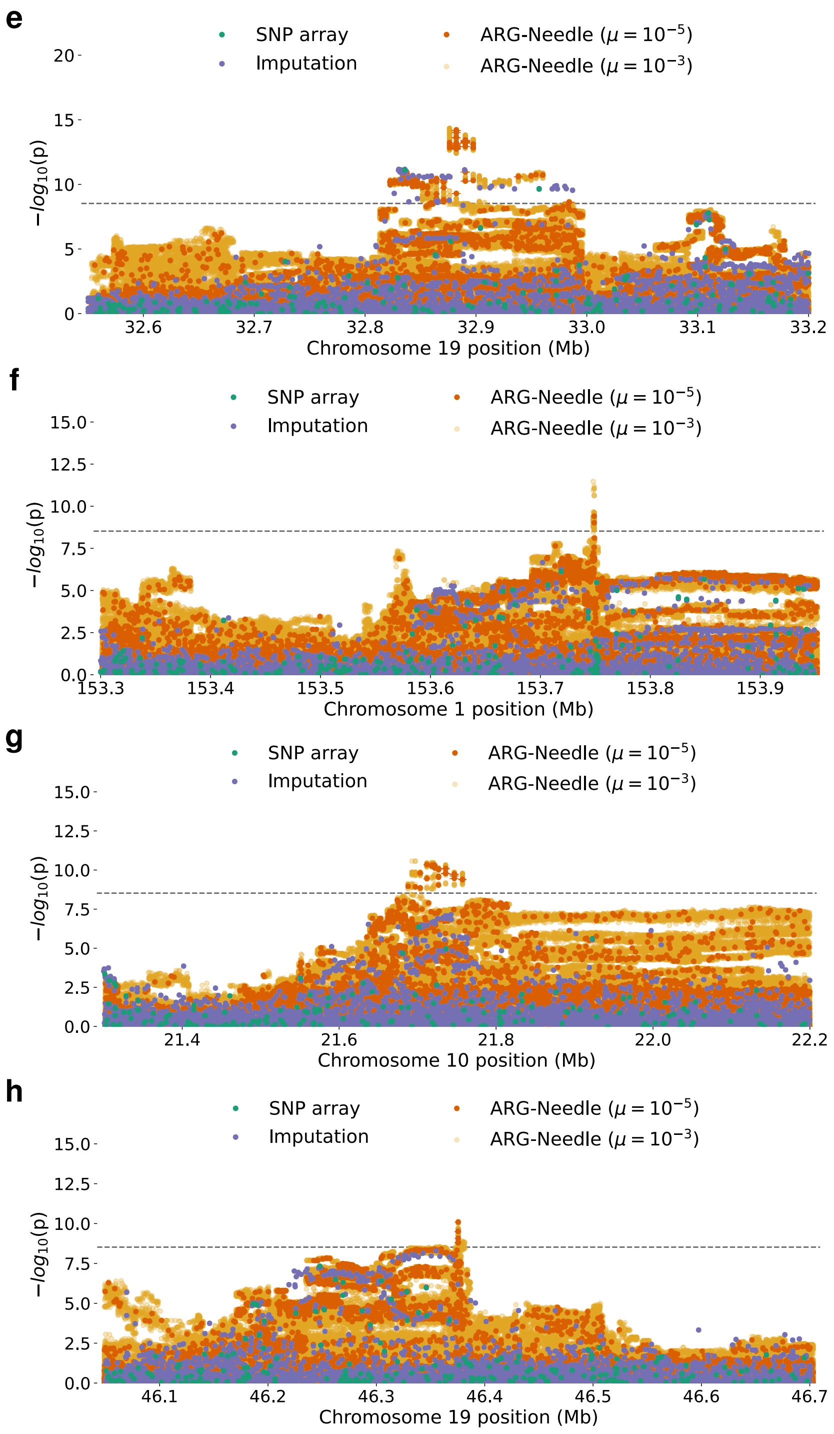

Supplementary Fig. 10: (Continued from previous page.) **Manhattan plots of higher frequency loci associated with height.** As in Fig. 5c-d but with eight additional loci of interest. **a**-**b**. Two loci where ARG-Needle detects an association peak within 10 kb as HRC+UK10K imputation from ∼65K haploid references, despite only using SNP array data. **c**-**e**. Three loci where ARG-Needle detects a different primary association peak than SNP array or imputed data association. **f**-**h**. Three regions where ARG-Needle ($\mu = 10^{-5}$) alone detects associations passing $p < 3 \times 10^{-9}$ significance. Dotted lines correspond to $p = 3 \times 10^{-9}$ (see Methods). The order of plotting is ARG-Needle with $\mu = 10^{-3}$, then ARG-Needle with $\mu = 10^{-5}$, then imputation, then SNP array variants on top. For the $\mu = 10^{-5}$ ARG associations crossing significance (here and in Fig. 5c-d), we additionally plotted a horizontal line showing the extent of the corresponding ARG clade.

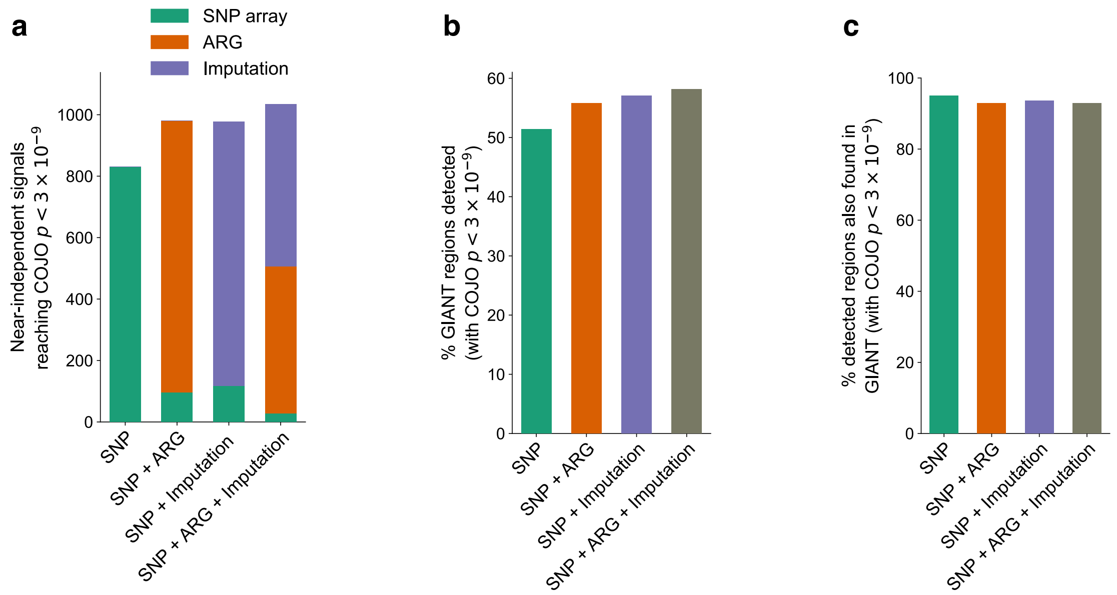

Supplementary Fig. 11: Genome-wide association of MAF $> 10\%$ variants with height in UK Biobank. a. Total number of independent MAF $> 10\%$ associations (defined as having COJO $p < 3 \times 10^{-9}$, see Methods) found and attribution based on data type. **b**. Percent of 1 Mb regions containing COJO associations in a GIANT consortium meta-analysis of $\sim$700K samples that are detected using COJO of MAF $> 10\%$ variants. **c**. Percent of 1 Mb regions containing independent MAF $> 10\%$ associations that were also present in the GIANT meta-analysis. These analyses used a genome-wide significance threshold of $p < 3 \times 10^{-9}$ as in Fig. 5; we found genome-wide resampling-based thresholds for MAF $> 10\%$ to be 3.4×10^{-8} (95% CI: [2.5×10^{-8}, 4.6×10^{-8}]) for ARG data and 4.7×10^{-8} (95% CI: [2×10^{-8}, 1.1×10^{-7}]) for imputed data (see Supplementary Table 1).

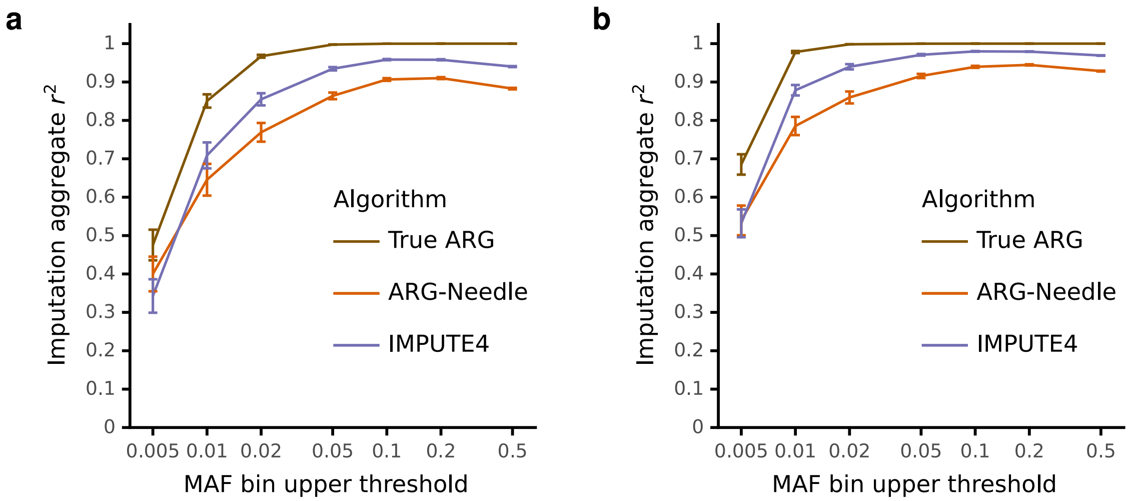

Supplementary Fig. 12: Joint array and sequencing ARG inference for genotype imputation. Exploratory ARG-based imputation analysis using the true ARG and an ARG-Needle inferred ARG compared to IMPUTE4 (see Supplementary Note 4 and Extended Data Fig. 5b). Simulations use a 10 Mb region, 300 (**a**) or 1000 (**b**) haploid sequencing samples, and 1000 haploid array samples. For IMPUTE4, we input a genetic map corresponding to the recombination rate used for simulation, and otherwise used default parameters. Variants were binned by MAF in the sequencing samples, and we report the aggregate r^2 within each bin (mean over 25 independent simulations), with each bin represented by its maximal MAF. Error bars represent 2 s.e. Imputation using the true ARG achieves highest accuracy, with performance that depends on MAF and reference sample size; imputation using ARG-Needle underperforms for high frequency variants but improves for the most rare variants.
