# Supplementary material for: Biobank-scale inference of ancestral recombination graphs enables genealogical analysis of complex traits
Source: Nat Genet. 2023 May 1;55(5):768–76. doi: 10.1038/s41588-023-01379-x (PMC10181934; doi:10.1038/s41588-023-01379-x)
Supplement: Supplementary file 2 — Reporting Summary [file 41588_2023_1379_MOESM2_ESM.pdf]

nature portfolio

Corresponding author(s): Pier Palamara
Brian Zhang

Last updated by author(s): Mar 19, 2023

Reporting Summary

Nature Portfolio wishes to improve the reproducibility of the work that we publish. This form provides structure for consistency and transparency in reporting. For further information on Nature Portfolio policies, see our Editorial Policies and the Editorial Policy Checklist.

Statistics

For all statistical analyses, confirm that the following items are present in the figure legend, table legend, main text, or Methods section.

n/a	Confirmed	
☐	☒	The exact sample size (*n*) for each experimental group/condition, given as a discrete number and unit of measurement
☒	☐	A statement on whether measurements were taken from distinct samples or whether the same sample was measured repeatedly
☐	☒	The statistical test(s) used AND whether they are one- or two-sided<br>*Only common tests should be described solely by name; describe more complex techniques in the Methods section.*
☐	☒	A description of all covariates tested
☐	☒	A description of any assumptions or corrections, such as tests of normality and adjustment for multiple comparisons
☐	☒	A full description of the statistical parameters including central tendency (e.g. means) or other basic estimates (e.g. regression coefficient) AND variation (e.g. standard deviation) or associated estimates of uncertainty (e.g. confidence intervals)
☐	☒	For null hypothesis testing, the test statistic (e.g. *F*, *t*, *r*) with confidence intervals, effect sizes, degrees of freedom and *P* value noted<br>*Give P values as exact values whenever suitable.*
☐	☒	For Bayesian analysis, information on the choice of priors and Markov chain Monte Carlo settings
☒	☐	For hierarchical and complex designs, identification of the appropriate level for tests and full reporting of outcomes
☐	☒	Estimates of effect sizes (e.g. Cohen's *d*, Pearson's *r*), indicating how they were calculated

Our web collection on statistics for biologists contains articles on many of the points above.

Software and code

Policy information about availability of computer code

Data collection	No software was used in data collection.
Data analysis	The arg-needle and arg-needle-lib software packages, which implement the ARG-Needle and ASMC-clust methods as well as methods for the main analyses in this paper, are available at https://palamaralab.github.io/software/argneedle/. Python packages can be downloaded at https://pypi.org/project/arg-needle/ and https://pypi.org/project/arg-needle-lib/; analysis scripts are available at https://doi.org/10.5281/zenodo.7745745. External software used in the current study were obtained from the following URLs: msprime (v0.7.4), https://pypi.org/project/msprime/; tsinfer (v0.1.4), https://pypi.org/project/tsinfer/; tsinfer scripts for sparse data (accessed Jan 2022), https://github.com/mcveanlab/treeseq-inference; Relate (v1.0.15), https://myersgroup.github.io/relate/; ARGON (v0.1.160415), https://github.com/pierpal/ARGON/; DASH (v1.1) and GERMLINE (v1.5.3), http://www1.cs.columbia.edu/~gusev/dash/; IMPUTE4 (v4.1.2), https://jmarchini.org/software/#impute-4; Beagle (v5.1), https://faculty.washington.edu/browning/beagle/b5_1.html; PLINK (v1.90b6.21), https://www.cog-genomics.org/plink/; PLINK (v2.00a3LM), https://www.cog-genomics.org/ plink/2.0/; GCTA (v1.93.2), https://cnsgenomics.com/software/gcta/; BOLT-LMM (v2.3.2), https://alkesgroup.broadinstitute.org/BOLT-LMM/downloads/; LiftOver (used April 2021), https://genome.ucsc.edu/cgi-bin/hgLiftOver.

For manuscripts utilizing custom algorithms or software that are central to the research but not yet described in published literature, software must be made available to editors and reviewers. We strongly encourage code deposition in a community repository (e.g. GitHub). See the Nature Portfolio guidelines for submitting code & software for further information.

Data

Policy information about availability of data

All manuscripts must include a data availability statement. This statement should provide the following information, where applicable:

- Accession codes, unique identifiers, or web links for publicly available datasets
- A description of any restrictions on data availability
- For clinical datasets or third party data, please ensure that the statement adheres to our policy

COJO association signals for higher frequency ARG variants with height are available at https://doi.org/10.5281/zenodo.7411562. VEP annotations were generated using the Ensembl VEP tool (v101.0, output produced February 2021), https://www.ensembl.org/info/docs/tools/vep/index.html. UK Biobank data can be accessed by approved researchers through https://www.ukbiobank.ac.uk/. Other datasets were downloaded from the following URLs: summary statistics from whole exome imputation from 50K sequences, https://data.broadinstitute.org/lohlab/UKB_exomeWAS/; likely causal associations from whole exome imputation from 50K sequences, https://www.nature.com/articles/s41588-021-00892-1 Supplementary Table 3; GIANT consortium summary statistics in ~700K, https://portals.broadinstitute.org/collaboration/giant/index.php/GIANT_consortium_data_files.

Field-specific reporting

Please select the one below that is the best fit for your research. If you are not sure, read the appropriate sections before making your selection.

☒ Life sciences ☐ Behavioural & social sciences ☐ Ecological, evolutionary & environmental sciences

For a reference copy of the document with all sections, see nature.com/documents/nr-reporting-summary-flat.pdf

Life sciences study design

All studies must disclose on these points even when the disclosure is negative.

Sample size	For real data analysis in the UK Biobank, we included all 337,464 individuals of White British ancestry (as reported in Bycroft et al. Nature 2018) whom did not have genotype missingness > 10% and had not withdrawn from the UK Biobank at the time of our analysis. To further explore our findings using exome sequencing data, we selected the 138,039 of these individuals who were exome sequenced in the 200K UK Biobank whole exome sequencing release.
Data exclusions	We excluded individuals who had withdrawn from the UK Biobank at the time of our analysis and individuals with genotype missingness > 10%.
Replication	The best tagged whole exome sequencing (WES) variants uniquely identified by our rare and ultra-rare ARG associations were validated using the likely-causal WES variants reported in Barton et al. Nature Genetics 2021. We found that 14/30 rare and 28/54 ultra-rare tagged WES variants were also detected as likely-causal associations (at p<5e-8) in Barton et al. Higher-frequency ARG associations with height were validated using GIANT consortium meta-analysis of 700K individuals comprising the UK Biobank and additional cohorts. A significant fraction (54/92, permutation p<0.0001) of regions identified uniquely using the ARG contained significant associations (p<3e-9) in this meta-analysis.
Randomization	There was no allocation into experimental groups in this study. When performing our phenotypic association scans, we controlled for covariates by first stratifying by sex and performing quantile normalization. We then regressed out age, age squared, genotyping array, assessment center, and the first 20 genetic principal components computed in Bycroft et al. Nature 2018.
Blinding	Blinding was not applicable to the analyses we performed within the UK Biobank, which analyzed all samples jointly. Data collection was performed previously by the UK Biobank.

Reporting for specific materials, systems and methods

We require information from authors about some types of materials, experimental systems and methods used in many studies. Here, indicate whether each material, system or method listed is relevant to your study. If you are not sure if a list item applies to your research, read the appropriate section before selecting a response.

Materials & experimental systems

n/a	Involved in the study	
☒	☐	Antibodies
☒	☐	Eukaryotic cell lines
☒	☐	Palaeontology and archaeology
☒	☐	Animals and other organisms
☒	☐	Human research participants
☒	☐	Clinical data
☒	☐	Dual use research of concern

Methods

n/a	Involved in the study	
☒	☐	ChIP-seq
☒	☐	Flow cytometry
☒	☐	MRI-based neuroimaging
